# Interplay between Mitochondrial Protein Import and Respiratory Complexes Assembly in Neuronal Health and Degeneration

**DOI:** 10.3390/life11050432

**Published:** 2021-05-11

**Authors:** Hope I. Needs, Margherita Protasoni, Jeremy M. Henley, Julien Prudent, Ian Collinson, Gonçalo C. Pereira

**Affiliations:** 1 School of Biochemistry, University of Bristol, Bristol BS8 1TD, UK; hope-isobel.needs@bristol.ac.uk (H.I.N.); j.m.henley@bristol.ac.uk (J.M.H.); 2 Medical Research Council-Mitochondrial Biology Unit, University of Cambridge, Cambridge CB2 0XY, UK; mp802@mrc-mbu.cam.ac.uk (M.P.); julien.prudent@mrc-mbu.cam.ac.uk (J.P.); 3 Centre for Neuroscience and Regenerative Medicine, Faculty of Science, University of Technology Sydney, Ultimo, NSW 2007, Australia

**Keywords:** protein import, mitochondrial dysfunction, respiratory complex assembly, supercomplexes, neurodegeneration, mitochondrial proteostasis

## Abstract

The fact that >99% of mitochondrial proteins are encoded by the nuclear genome and synthesised in the cytosol renders the process of mitochondrial protein import fundamental for normal organelle physiology. In addition to this, the nuclear genome comprises most of the proteins required for respiratory complex assembly and function. This means that without fully functional protein import, mitochondrial respiration will be defective, and the major cellular ATP source depleted. When mitochondrial protein import is impaired, a number of stress response pathways are activated in order to overcome the dysfunction and restore mitochondrial and cellular proteostasis. However, prolonged impaired mitochondrial protein import and subsequent defective respiratory chain function contributes to a number of diseases including primary mitochondrial diseases and neurodegeneration. This review focuses on how the processes of mitochondrial protein translocation and respiratory complex assembly and function are interlinked, how they are regulated, and their importance in health and disease.

## 1. Introduction

Mitochondria provide the main source of cellular energy in the form of ATP. This is particularly important in high energy consuming cells such as cardiac and muscle cells as well as in neurons. On top of this vital role in ATP synthesis, mitochondria have a plethora of other roles, including regulation of cellular metabolism, calcium storage and signalling, reactive oxygen species (ROS) signalling, damage-associated molecular patterns (DAMPs) production in inflammation and immunity, and programmed cell death [1]. The presence of key enzymes and proteins in different submitochondrial compartments is indispensable for these roles. Consequently, protein translocation becomes a fundamental process for efficient mitochondrial physiology. Due to their diverse proteome, mitochondria have distinct import pathways, which must be fully operational to maintain a healthy organelle [2]. The first section of this review will cover how cytoplasmic translated proteins are imported into mitochondria, as well as how mitochondrial-encoded proteins are translocated from the matrix to the inner mitochondrial membrane (IMM). We then explore the special case of respiratory complexes, which are multimeric proteins assembled from subunits encoded by both the nuclear and mitochondrial genomes to highlight the importance of the import machineries.

Due to the fundamental importance of mitochondrial homeostasis for the regulation of multiple central processes and pathways, it is not surprising that mitochondrial defects, and more specifically mitochondrial import defects, have been implicated in several diseases [3]. These include most neurodegenerative diseases [4], as well as mitochondrial diseases associated with deficiencies of the respiratory complexes due to mutations affecting the import machineries [5]. These will be discussed later, followed by a discussion of the recent advances therein and with respect to the most common mitochondrial stress response and proteostatic pathways thought to counteract import dysfunction.

## 2. Protein Translocation

**Figure 1:**
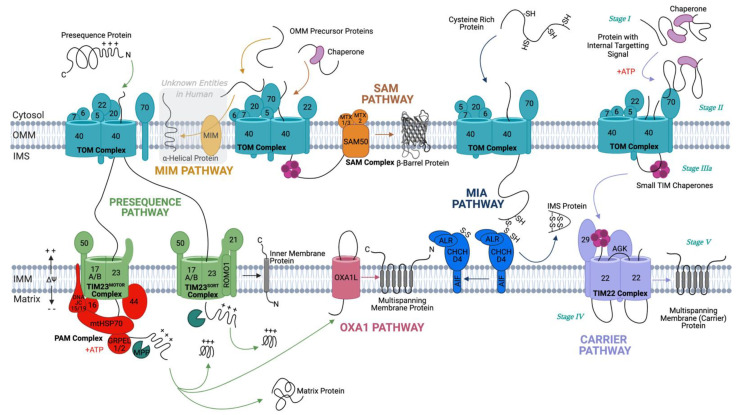
Overview of human mitochondrial protein import pathways. The TOM complex acts as the central entry gate for precursor proteins to enter the IMS, where they are diverted into one of five pathways, depending on their structure, function, and target destination. The MIM pathway (only currently understood in yeast) is an exception in that proteins usually do not cross the Tom40 channel. Instead, OMM α-helical proteins are recognised by Tom70 and transferred through MIM to be inserted into the OMM. The five major pathways proteins take after crossing the TOM channel are the following. The presequence pathway: Presequences containing precursor proteins are transported via the presequence pathway. Of these proteins, proteins with a hydrophobic sorting sequence are inserted into the IMM by the TIM23^SORT^ complex, whereas hydrophilic matrix proteins are pulled through the TIM23^MOTOR^ complex, with the help of the PAM complex and ATP hydrolysis cycles. The presequences of both these groups of proteins are cleaved by MPP on the matrix side. The OXA1 pathway: N-terminally inserted multispanning membrane proteins, once passed through TIM23^MOTOR^ and cleaved by MPP, are passed to OXA1L, which inserts them into the IMM in the N-terminal formation. OXA1L is also responsible for the insertion of mtDNA encoded proteins into the IMM. The SAM pathway: β-barrel proteins are transported to the TOM complex by cytoplasmic chaperones. They are then passed through the TOM complex and received by small TIM chaperones on the other side for insertion into the OMM by the SAM complex. The MIA pathway: Cysteine-rich proteins in an unfolded, reduced state are passed via the TOM complex to the MIA complex, which inserts disulphide bonds in them, allowing them to reside in a folded, oxidised state in the IMS. Carrier pathway: Proteins with internal targeting signals are protected in the cytosol by cytosolic chaperones (Stage I), which pass them to the TOM complex (Stage II). They are received on the IMS side by small TIM chaperones (Stage III), which transfer them through the IMS to the TIM22 complex (Stage IV) for insertion into the IMM (Stage IV).

All but 13 of the estimated >1000 human mitochondrial proteins [6] required to perform key mitochondrial functions are encoded by the nucleus and synthesised on cytoplasmic ribosomes and thus must be imported into mitochondria through highly conserved protein translocation pathways (Figure 1). Owing to the double membrane bound structure of mitochondria, these multistep protein translocation pathways involve numerous protein complexes (Figure 1 and Table 1). Moreover, their proteome consists of soluble, membrane-bound, and transmembrane proteins with different mitochondrial sub-localisations. Therefore, specialised import machinery has evolved to successfully import all classes of proteins.

### 2.1. Crossing the Outer Membrane

All proteins destined to the mitochondria must first cross the outer mitochondrial membrane (OMM), which they gain access to via the translocase of the outer membrane (TOM) complex (Figure 1). The TOM core complex (TOM-CC) consists of five components: TOM40, TOM22, TOM7, TOM6, and TOM5. The TOM holo-complex is formed following weak association of the TOM-CC with an additional two subunits: TOM20 and TOM70 [2,7]. These subunits are highly conserved between humans and yeast (Table 1); however, we refer to the yeast translocases in the following section, since this was the first organism it was discovered in. Precursor proteins are recognised by the receptor proteins Tom20, which recognises proteins with a mitochondrial targeting sequence (MTS), i.e., presequence proteins [8,9], and Tom70, which specifically recognises precursors with internal targeting signals, such as those belonging to the solute carrier family (SLC25) [10,11]. Proteins are then passed to the Tom40 pore via another receptor component, Tom22, which has also been shown to assist in the assembly of the TOM complex [12,13,14]. Tom22 physically interacts with Tom40 via its transmembrane segment, whilst its cytosolic domain has been suggested to act as a docking site for the other receptor proteins, Tom70 and Tom20. Recently, the OMM porin metabolite channel (also known as the voltage-dependent anion channel) has been reported to regulate Tom22 integration into the TOM complex in yeast, thus regulating the assembly and stability of the TOM complex [15,16]. Por1, the major yeast isoform of Porin, binds newly imported Tom22 and integrates it into the TOM complex, promoting formation of the mature trimeric form of the TOM complex required for import of precursor proteins [15]. Por1 also sequesters dissociated Tom22, stabilising the dimeric TOM complex under situations where this is preferable, i.e., for the import of proteins destined for the mitochondrial intermembrane space (IMS) assembly (MIA) pathway [15]. Porin is also thought to cooperate with Tom6 in regulating trimeric TOM assembly and stability and thereby modulating protein import during the cell cycle [15,17].

The different oligomeric states of the TOM complex and the nature of these different states remains unclear. Whilst it had generally been accepted that the mature form of TOM complex exists as a trimer [18,19,20,21], a cryogenic electron microscopy (cryo-EM) study in *Neurosposa crassa* showed the TOM complex in a dimeric form [22]. More recently, high resolution cryo-EM studies in *Saccharomyces cerevisiae* showed that the TOM-CC exists as dimers and tetramers. The latter is essentially a dimer of the dimeric form of TOM-CC, achieved by lateral stacking of the dimeric TOM complex [7]. Due to the dynamic properties of the TOM complex, it may be proposed that the trimeric complex is formed by dissociation of a monomer from the tetrameric form.

Of note, the only protein of the TOM complex with a significant IMS domain is Tom22, which is important for its role in directing emerging precursor proteins to the Tim50 receptor of the translocase of the inner membrane 23 (TIM23) complex for further translocation [23]. Structural analysis of the interactions between these differing structural subunits showed that association is mainly mediated by hydrophobic interactions, along with high surface complementarity between the transmembrane domains [7].

### 2.2. Biogenesis of OMM Proteins

Evidence has also shown that, in yeast, Tom40 may simultaneously act as an insertase, assisting in the lateral release and insertion of proteins destined for the OMM. However, this is highly dependent on specific determining factors within the precursor sequence and is not yet fully understood [24]. Although this initial observation was monitored using an artificial import substrate, it has since been suggested that a similar process might be responsible for the accumulation of high-molecular weight PINK1 in the OMM in a TOM7-dependent manner in human cells [25,26].

Recently, it has been proposed that in addition to their role in quality control, PINK1/Parkin also regulate protein import under physiological conditions where mitochondrial function remains normal [27]. It is proposed that ‘local dysfunction’, as in mitochondrial membrane potential (Δψ) depolarisation or import efficiency, is sensed by the PINK1/Parkin pair, which phosphorylates several subunits of the TOM complex, namely Tom20, Tom70 and Tom22, facilitating the import of presequence precursors. Importantly, the ubiquitylation pattern under this condition is significantly different from the PINK1/Parkin activation experienced from global mitochondrial dysfunction. Conversely, the mitochondrial ubiquitylase USP30 antagonises these effects [27,28,29]. Additionally, USP30 was shown to work in a reciprocal manner to MARCH5, a E3 ubiquitin-protein ligase of the OMM, under basal conditions, for deubiquitinating presequence substrates during translocation, facilitating their import. For other regulatory mechanisms of protein import, please see [30].

#### 2.2.1. Insertion of β-Barrel Proteins in the OMM

Precursors of β-barrel proteins destined to be inserted into the OMM are passed via small TIM chaperone proteins to the sorting and assembly machinery (SAM) complex, for insertion into the OMM [31,32]. The SAM pathway has been described in detail in another review [33]. The human SAM complex consists of accessory subunits MTX2 (yeast Sam35), MTX1, and MTX3 (yeast Sam37) and OMM associated β-barrel core subunit SAM50 (yeast Sam50; Table 1) [34]. In yeast, β-barrel precursor proteins are translocated through the TOM complex, where they are bound by small TIM chaperones and transferred through the IMS to the SAM complex (Figure 1). Substrate proteins are recognised by Sam35, which interacts with the β-signal located in the last strand of the substrate protein. This initiates insertion into Sam50, which is responsible for folding and inserting substrates into the OMM [32]. Sam37 is required for substrate release and has also recently been proposed to assist in the formation of a SAM-TOM supercomplex, mediated by physical interaction of Sam37 and Tom22 on the cytosolic side of the OMM [35]. This SAM–TOM interaction has been shown to be essential for coupling of the two OMM complexes and promoting efficient precursor transfer [35]. Though not a part of the core SAM complex, Mdm10 is thought to associate with the SAM complex and have an important role in Tom40 assembly into the TOM complex [36]. This pathway is very similar to that observed for β-barrel proteins of the outer membrane in bacteria, which are folded and inserted into the outer membrane by the bacterial assembly machinery (BAM) complex, the *E.Coli* homolog of SAM [34].

#### 2.2.2. Incorporation of α-Helical Anchors in the OMM

Over 90% of integral OMM proteins contain α-helical membrane anchors, yet the import pathway undertaken by these proteins is still relatively poorly understood, particularly in humans [37]. In yeast, the majority of these proteins are recognised by the Tom70 receptor of the TOM complex and passed on to the insertase of the outer mitochondrial membrane (MIM) complex, which aids in their insertion into the OMM (Figure 1 and Table 1) [38,39]. Multiple copies of Mim1 arrange themselves in such a way that, when reconstituted into the lipid bilayer, a channel is formed, and along with a couple of copies of Mim2, this establishes the MIM complex [40,41].

There are, however, known exceptions to this rule, whereby these α-helical proteins are passed through the Tom40 channel into the IMS prior to insertion into the OMM, aided by the MIM complex [42,43]. Interestingly, one of these proteins, yeast Om45, has been shown to require the TOM, TIM23, and MIM complexes for insertion into the OMM, where it is anchored by its N-terminal signal sequence with the bulk of the protein exposed to the IMS [42]. The final topology of Om45 is thus opposite to the N_in_-C_out_ topology typical of MIM pathway proteins. The other known exception, yeast Mcp3, is also directed via TOM and TIM23, but is then processed by the inner mitochondrial membrane protease (IMP) before being transferred via MIM and inserted into the OMM with a final topology of N_out_-C_out_ [43]. Notably, whilst both proteins interact with components of the TIM23 complex and are dependent on Δψ, they do not cross or interact with the IMM [42,43].

### 2.3. Co- and Post-Translational Translocation

Importantly, preproteins must be unfolded in the cytosol and subsequently stabilised, in an ATP dependent process, by molecular chaperones of the heat shock protein (hsp) families Hsp70 and Hsp90, to then be efficiently imported [44,45]. Conversely, the subsequent translocation of these unfolded preproteins through the TOM channel occurs independently of ATP and Δψ, and instead relies on an indirect driving force. That is the increased affinity of the presequences for the *trans* over *cis* side of the TOM channel, allowing transport of the preproteins across the channel where the presequence is bound by TIM50 [46]. This transport is also thought to rely on the sequential binding of the presequence to acidic domains of receptor proteins in what is known as the ‘acid chain’ hypothesis [47].

Interestingly, whilst the majority of preproteins are synthesised in the cytosol and must be unfolded prior to insertion into the TOM complex, others are unable to be imported into mitochondria post-translationally, and instead must undergo co-translational translocation whereby cytosolic ribosomes associate with mitochondria [48]. For this subgroup of proteins, it is thought that signals within the 3′-untranslated region (UTR) and coding regions of their mRNAs mediate their targeting to the cytosolic side of the OMM [49,50,51], where cytosolic ribosomes have also been observed [52,53].

### 2.4. Staying in the Intermembrane Space—The Disulfide Relay System

Proteins destined for the IMS take the route of the MIA pathway (Figure 1 and Table 1), which has been reviewed in great detail previously [54]. This class of proteins lack an MTS, are generally small, and share a conserved coiled coil-helix1-coiled coil-helix 2 domain (CHCHD). These cysteine-rich proteins contain two pairs of cysteines separated by three or nine amino acid residues (Cx_3_C or Cx_9_C) in the helices [54]. The small TIM chaperones of the IMS, important for translocase of the inner mitochondrial membrane 22 (TIM22)-dependent translocation described below, and assembly factors of IMM proteins, such as the respiratory complexes (see below and Table S1), are some examples of MIA substrates. The substrates are also relatively unstable and prone to degradation prior to their reduction by the relay system [55]. These cysteine-rich proteins undergo oxidation driven import whereby, upon passing through the TOM complex in an unfolded, reduced state, they form transient disulphide bonds with Mia40 [56,57]. CHCHD4 is the human ortholog of yeast Mia40 and shows high conservation despite the smaller size (16 vs. 40 kDa, respectively), lack of MTS, and no transmembrane anchor domain [58]. Instead, the human CHCHD4 interacts with the apoptosis inducing factor (AIF) and its cofactor NADH for association with the IMM [58].

The second player in the MIA pathway is ALR (Erv1 in yeast), a FAD-linked sulfhydryl oxidase that enables new rounds of precursor import and oxidation by re-oxidising reduced CHCHD4 after it has carried out its role as an oxidoreductase, thus allowing the cycle to continue [59]. Similarly, reduced ALR can relay its electrons to cytochrome *c* and, afterwards, to CIV of the respiratory chain [60]. Therefore, despite not requiring ATP or Δψ to operate, the MIA pathway still depends on a functional electron transport chain (ETC) to successfully oxidise its substrates.

### 2.5. Crossing or Insertion in the Inner Membrane

Proteins that are destined elsewhere within the mitochondria, namely the matrix or its membrane, must subsequently pass through or into the IMM (Figure 1). This membrane crossing (or insertion) import event is facilitated by one of two translocase complexes, the translocase of the inner mitochondrial membrane 23 (TIM23) complex, or the TIM22 complex.

#### 2.5.1. TIM23 Complex (Presequence Pathway)

Precursor proteins destined for the mitochondrial matrix, along with some IMM sorted proteins, containing an N-terminal presequence (i.e., MTS), are passed directly from the TOM complex to the TIM23 complex [2,30]. The MTS is a cleavable region of 15 to 50 amino acids that precedes the mature protein and which is rich in hydrophobic, hydroxylated, and basic residues, with an overrepresentation of arginine residues and a near absence of acidic residues, forming a positively charged, amphipathic α-helix [61]. Interestingly, it has recently been suggested that preproteins may also contain additional internal MTS-like signal sequences (iMTS), located in the mature region of the preprotein, which act similarly to presequences and mediate the binding of the preprotein to Tom70, increasing the efficiency of protein import via the presequence pathway [62].

The TIM23 complex is anchored to the IMM and exists as a hetero-oligomeric complex, composed of various subunits (Table 1). It consists of an integral membrane embedded core complex as well as an import motor [63]. The core complex contains three essential subunits: TIM17A/B, TIM23, and TIM50 (Tim17, Tim23, and Tim50 in yeast) [46,64,65,66]. Additionally, the membrane-embedded part has two non-essential subunits: TIM21 and ROMO1 (Tim21 and Mgr2 in yeast) [12,67]. The import motor, also known as the presequence translocase-associated motor (PAM) complex, drives translocation across the IMM, aided by ATP hydrolysis, and consists of TIM44, mtHSP70, DNAJC15/19, TIM16, and GRPEL1/2. In yeast, the homologs are Tim44, SSC1 (also known as mtHsp70), Tim16 (also known as Pam16), Tim14 (also known as Pam18), and Mge1, as well as Pam17, which is not known to have a human homolog [68,69,70,71,72,73].

In yeast, precursor proteins released from the TOM complex and destined for the presequence pathway are recognised by Tim50 and the IMS region of Tim23, which act as receptor proteins for the incoming precursors [63]. This is achieved by binding of the hydrophilic, IMS-exposed part of the Tim23 subunit and the IMS-extending part of the Tim50 subunit in the IMS [46,65,66,74]. The Tim23 pore acts as a voltage gated channel and is ~13 Å wide, thus wide enough for only one α-helix to pass through at a time [75,76]. The pore is formed by the hydrophobic, C-terminal membrane domain of Tim23, and Tim17, which has been shown in the yeast model to be important for formation of the twin-pore structure, since it is unable to form in Tim17-depleted mitochondria [77]. In the handover of proteins from the TOM complex to the TIM23 complex, Tim50 also interacts with various partner proteins, including Tom22 and Tom21, which are necessary for the correct recognition and direction of precursor proteins across the IMS to the TIM23 channel [74,78,79,80]. Notably, it has recently been shown that phosphorylation/dephosphorylation of mammalian TIM50 is required for regulation of import activity, that is, phosphorylation of TIM50 reduces mitochondrial import, whilst its dephosphorylation by human phosphatase PPTC7 enhances it [81]. TIM50/Tim50 is phosphorylated on its matrix-facing segment in both mouse and yeast (T33 and S103, respectively) [81], but the identity of the kinase(s) responsible for this effect is still unknown. Furthermore, various matrix proteins were found to have phosphorylation sites around their MTS, the dephosphorylation of which is also thought to be important for enhancing their import and processing within the matrix [81]. This study highlights the importance of further work to dissect the currently unclear mechanisms regulating translocation.

The crossing of precursor proteins across the import channel of the IMM is driven by a number of forces: the proton motive force, i.e., Δψ and ΔpH, the affinity of the presequence for the *cis* side over the *trans* side of the membrane, and ATP hydrolysis [63,82]. As mentioned above, the higher affinity of presequences towards Tim50 initiates the handover from TOM to TIM23 complex. Additionally, the positively charged MTS means that the Δψ across the IMM exerts an electrophoretic effect on the proteins, facilitating the threading through TIM23.

As soon as the precursor emerges from the channel, it immediately interacts with Tim44. Importantly, it was shown that the affinity of presequences is higher for Tim44 compared to Tim50 [78], strengthening the directionality of presequence movement across the IMM. Additionally, Tim44 is known to act as a scaffold and to recruit the PAM complex (Table 1) [83]. In this model, one arm of Tim44 is anchored to Tim23 while another arm is dynamic and interacts with mtHsp70, Tim16 and, indirectly, Tim14, controlling the active:inactive state of the motor [76]. A typical cycle would involve the recruitment of ATP-bound mtHsp70 followed by a loose binding to the emerging precursor. Then, Tim14 would stimulate the ATPase activity of mtHsp70, trapping the bound polypeptide and consequently releasing the chaperone from Tim44, allowing the sliding of the precursor:chaperone complex into the matrix. The binding of Mge1 to this complex in the matrix allows the release of ADP and subsequent binding of a new ATP molecule coupled with the release of bound precursor [84]. The presequences are cleaved off by mitochondrial processing peptidase (MPP), leading to protein folding and maturation [12].

Nonetheless, not all precursors that are passed to the TIM23 complex are destined for the matrix. In fact, TIM23 is also responsible for the sorting and lateral insertion of membrane proteins into the IMM. These proteins contain a stop transfer signal, a region adjacent to the presequence of ~20 amino acids, which is rich in hydrophobic residues flanked by charged resides, also known as a sorting signal sequence, which targets them for this pathway of insertion [85]. The assembled TIM23 complex responsible for protein insertion into the IMM differs from the motor associated TIM23 in that it contains TIM21 (Tim21) and ROMO1 (Mgr2) and lacks the PAM complex [12], since it does not require the motor activity, but is instead driven supposedly solely by Δψ [85,86]. For these reasons, the motor-associated TIM23 complex is known as TIM23^MOTOR^ complex, whilst the lateral release TIM23 complex is known as the TIM23^SORT^ complex. Tim21 is important in regulating the lateral release of IMM proteins [87,88]. Furthermore, Mgr2 is important in aiding the binding of Tim21 to the TIM23^SORT^ complex, as well as in the lateral release of proteins into the IMM [89]. The ability of Mgr2 to be crosslinked to precursors in transit suggests that it may make up part of the channel [67].

#### 2.5.2. TIM22 Complex (Carrier Pathway)

In the previous section, we described how proteins resident in the IMM, containing a single transmembrane domain and a mitochondrial targeting sequence, use the TIM23 complex for insertion. However, some hydrophobic proteins destined for the IMM are synthesised without a presequence and comprise multiple transmembrane domains and consequently, require a different import pathway named TIM22 or carrier pathway [90,91,92]. The majority of these proteins belong to the solute carrier family, typically containing six α-helical domains with multiple internal targeting sequences within the mature protein [90,93]. However, the exact mechanism by which these internal targeting sequences target carrier proteins to the IMM remains to be fully elucidated. The carrier pathway is particularly important for mitochondrial protein translocation as a whole since some of its substrates include translocase subunits Tim17, Tim22, and Tim23 [94].

Recent cryo-EM studies have determined the structure of the human TIM22 complex at 3.7 Å from overexpression in HEK293T cells [95] and yeast TIM22 at 3.8 Å resolution from endogenous protein levels [96]. The obtained models revealed notable structural differences between the two. Human TIM22 is a complex of ~440 kDa, and the cryo-EM structure (approx. 100 Å height and 160 Å width) revealed six subunits: TIM22, TIM29, acylglycerol kinase (AGK), TIM9, TIM10A, and TIM10B (Table 1) [95]. This structure shows the complex mainly extending into the IMS, along with a transmembrane region consisting of four transmembranes of TIM22 and one transmembrane of TIM29 and AGK. TIM29 acts as a scaffold, holding both TIM9-TIM10A-TIM10B and AGK in proximity to the TIM22 channel. The human TIM22 structure showed the chaperone ring to be tilted at a 45° angle [95]. It is also thought that TIM29 links the TIM22 and TOM complexes, mediating transfer of the carrier protein, a link that has not yet been shown in yeast [97,98]. Recent studies have revealed that AGK, which is involved in lipid biosynthesis, is important for TIM22 assembly and function [99,100].

In yeast, the TIM22 complex is ~300 kDa and consists of seven subunits: Tim22, Tim18, Tim54, Sdh3, Tim9, Tim10, and Tim12 (Table 1) [96]. The yeast structure showed that the small TIM subunits (Tim9–Tim10–Tim12) sit on the membrane in a hexameric ring formation and are anchored to the rest of the TIM22 complex via a docking platform consisting of Tim18-Sdh3 and Tim22. Tim54 is also required to hold Tim9–Tim10–Tim12 in a tilted conformation, like in humans, at around 45°, allowing them to receive substrates and pass them to the Tim22 channel [96]. Interestingly, Sdh3 is also a component of respiratory Complex II [101]. However, there is no evidence to suggest that the human Sdh3 homolog SDHC associates with the TIM22 complex.

Overall, the TIM22 carrier import pathway can be divided into five distinct and consecutive stages (Figure 1) with different energy requirements, producing perceivable transport intermediates to be monitored in vitro [102]. The stages are described in yeast below but are thought to be very similar in humans. In Stage I, the recently translated precursor is found in a soluble chaperone-bound form (chaperones of the Hsp70/Hsp90 families) not associated with mitochondria.

Then, during Stage II, the precursor–chaperone complex is passed on to the Tom70 receptor in an energy-independent manner, driven solely by the affinity of the receptor towards the precursor and the tetratricopeptide repeats in the chaperone. The Tom70 molecules contain two binding sites, one for the precursor and one for the chaperones [103], and aid in the transfer of the protein to Tom22 for insertion into the Tom40 channel [104,105]. More recently, the biological significance of Tom70 has been challenged, and it is suggested that the receptor acts as a general interface between cytosolic chaperones and the mitochondrial import machinery, and not as a specific receptor for carrier precursors [106]. In this regard, Tom70 would play a key role in reducing precursor-induced proteostasis stress. Next, ATP binding to the cytosolic chaperone triggers the release of the precursor and progression through the Tom40 channel. Importantly, the precursor can be arrested in Stage II by ATP depletion [102]. Interestingly, it is thought that carrier proteins are inserted into the Tom40 channel with both termini remaining in the cytosol, in a loop-like formation [107].

During Stage III, the precursor emerges from the IMS-facing side of the Tom40 channel, binding the small TIM chaperones (Tim9–Tim10), which tend to exist as hetero-hexameric complexes, for handover to the TIM22 complex. However, experimental data where Δψ was dissipated showed the accumulation of two distinct populations, suggesting that the following stages, namely insertion, are Δψ-dependent, and that Stage III is further divided in two sub-stages. Stage IIIa represents the precursor deeply inserted in the TOM complex and protected from exogenous proteases [102]. Stage IIIb represents a fully translocated precursor across the OMM, tethered to the TIM22-bound TIM chaperone complex (Tim9–Tim10–Tim12) via hydrophobic interactions [102]. Tim12 is bound to the TIM22 complex, and thus aids in passing chaperoned carrier proteins to the Tim22 channel via the Tim54 docking site. Recently, it has been shown in yeast that Porins can assist the translocation by recruiting and interacting with the TIM22 complex, forming contact sites between OMM/IMM, to spatially coordinate inner and outer membrane transport steps [108]. However, others have identified that these juxtapositions are maintained by the interaction of TIM22 with the mitochondrial contact site and cristae organising system (MICOS) complex in humans [109]. Conversely, MICOS is found in association with the TIM23 complex in yeast [109].

Interestingly, the last two stages of the translocation of carrier precursors show differential dependence on Δψ, confirmed experimentally through the use of ionophores [92]. Stage IV, also known as docking, can occur in a partially depolarised membrane (−120 to −60 mV) whereby the precursor is in full association with the TIM22 complex and one of its loops is inserted in the Tim22 channel [92]. Despite the low Δψ, the electrophoretic effect experienced by the positive charges on the matrix loops of the carrier precursor is apparently sufficient to drive its partial translocation into the complex. Finally, Stage V requires a fully energised membrane (>−120 mV) to successfully insert the carrier precursor into the IMM after lateral opening of TIM22 [92,102].

Recently, the canonical even-numbered paired transmembrane helices with N_out_- and C_out_-terminal rule for TIM22 substrates has been challenged [110]. In this report, authors observed that the yeast mitochondrial pyruvate carrier, which has an odd number of transmembrane segments and a matrix-facing N-terminus, was imported specifically via the TIM22 complex. Similarly, it has been recently reported that human sideroflexins, a class of IMM proteins that contain five transmembrane domains and that do not belong to the SLC25 family, are imported via TIM22 [111]. Therefore, we can assume that the TIM22 substrate spectrum is less intransigent and contains proteins with paired and non-paired transmembrane domains.

#### 2.5.3. Oxa1

Despite the endosymbiotic character of mitochondria, the organelle lacks a SecY-like translocon and possesses instead an import machinery that more closely relates to the bacterial membrane insertase YidC [112]. The so called IMM protein oxidase assembly protein 1 (OXA1L, OXA1 in yeast; Figure 1 and Table 1) is highly conserved from bacteria to mammals and plants [113].

OXA1 is nuclear-encoded, translated in the cytosol, and imported into the mitochondria by the TOM/TIM23 pathway via its N-terminal MTS in an mtHsp70- and ATP-dependent manner [114]. Interestingly, recently imported OXA1 is first observed in the matrix and then uses endogenous OXA1 to successfully insert itself into the IMM [114]. Mature OXA1 (36 kDa) is known to form oligomers, although its behaviour is still controversial. For example, in *Neurospora crassa*, it exists as a homo-tetramer [115], while human OXA1L has an apparent mass of 600–700 kDa, suggesting a hetero-oligomeric complex of unknown identity [116].

Since the majority of mtDNA-encoded proteins are highly hydrophobic, it is predictable that OXA1L interacts with mito-ribosomes for a co-translation process, whereby nascent chains associate with the insertase to suppress possible aggregation of the polypeptide in the matrix. This interaction occurs via the long C-terminus of OXA1L/OXA1, in both humans and yeast [117]. Recently, a cryo-EM structure showed an association between human OXA1L and mitochondrial ribosomes in a native state, coupling protein synthesis and membrane delivery [118].

In addition to its role in the insertion of mtDNA-encoded proteins, OXA1 is also responsible for N-terminal insertion of some nuclear-encoded proteins [119]. In these cases, proteins with N-terminal MTS are not arrested during import via TIM23^SORT^ but are fully imported into the matrix via TIM23^MOTOR^ and thereafter sorted for export from the matrix via OXA1 after cleavage of the MTS [119]. Similarly, multispanning proteins such as the ABC transporter Mdl1 can cooperatively make use of the stop-transfer (TIM23) and conservative (OXA1) sorting for integration into the IMM [120]. In regard to yeast Mdl1, the insertion topology occurs as follows: transmembranes 1 and 2 are imported via stop-transfer; the subsequent transmembranes 3 and 4 are imported into the matrix in an mtHSP60/ATP-dependent manner, and exported into the IMM via OXA1; transmembranes 5 and 6 are OXA1-independent and probably use the stop-transfer mechanism. Interestingly, the middle two TM helices 2 and 3 (of Mdl1), dependent on Oxa1 for their insertion, are not particularly hydrophobic. This ties in well with the noted evolutionary conservation and striking structural similarity of the Oxa1/YidC family with EMC3 of the ER membrane complex [121,122,123]. Given their common mechanism for membrane protein insertion, it is perhaps significant that the EMC is also recruited for the incorporation of TM helices with reduced hydrophobicity [124]. Therefore, the possibility that OXA1 assists more widely in the insertion of less-hydrophobic TM helices, such as those possessed by transporters (like Mdl1), proton translocators and carriers, is worthy of further investigation.

In regard to energy dependence, OXA1 does not require ATP for protein insertion, similarly to TIM22; however, its dependence on Δψ is not as obvious. For example, export of the N-terminus of nuclear-encoded proteins requires an energised membrane [125], as is the case for the mtDNA-encoded Cox2 yeast protein [126], but not for yeast Cox1, Cox3, or cytochrome *b* [126]. Interestingly, this same correlation is observed in regard to negative charges, i.e., substrates with negatively charged N-terminus and/or IMS loops are Δψ-dependent, while those with less negative or neutral character are not [127], suggesting that the content of charged residues in an IMM protein determines its dependence on the OXA1 translocase.

## 3. The Respiratory Chain and Supercomplexes

The IMM is extremely rich in protein content and accommodates among other classes a vital group of proteins known as the ETC. Under physiological conditions, the respiratory complexes forming the ETC can exist as individual entities and/or in association with one another to form high-order structures, known as supercomplexes (SC) [128]. Interestingly, it has been suggested that an important role of SC is to participate in the assembly and/or stability of single respiratory complexes [129,130,131]. In fact, defects in one complex can lead to multi-complex deficiencies [132,133,134]. Additionally, CI and CIII intermediates were found to bind CIII and CIV subunits before maturation of the respiratory complex [135].

There are numerous reported interactions between components of the ETC and the import machinery. For example, Tim21 and the two regulatory PAM subunits Pam16 and Pam18, all part of TIM23, were found to interact with SCIII_2_IV in yeast [86,136]. Moreover, human TIM21 was co-purified with CI assembly intermediates and identified as a CI interactor by complexome profiling [137]. Other import-related proteins have also been found to associate with respiratory complex subunits in yeast, such as mHsp70, which was found to interact with Mss51, an MTCO1 mRNA-specific translation activator [138], and also with CIV subunit Cox4 [139]. These interactions suggest a functional interdependence between the import machinery and the respiration complexes, which still needs to be clarified. Hypothetically, a direct interaction with the translocase system might favour a faster and more efficient regulation of the ETC complexes assembly, possibly in response to cellular signalling. Alternatively, the import machinery in the direct vicinity of proton-pumping respiratory complexes could benefit from the higher Δψ [140] required for protein import.

### 3.1. Respiratory Complexes Assembly

**Figure 2:**
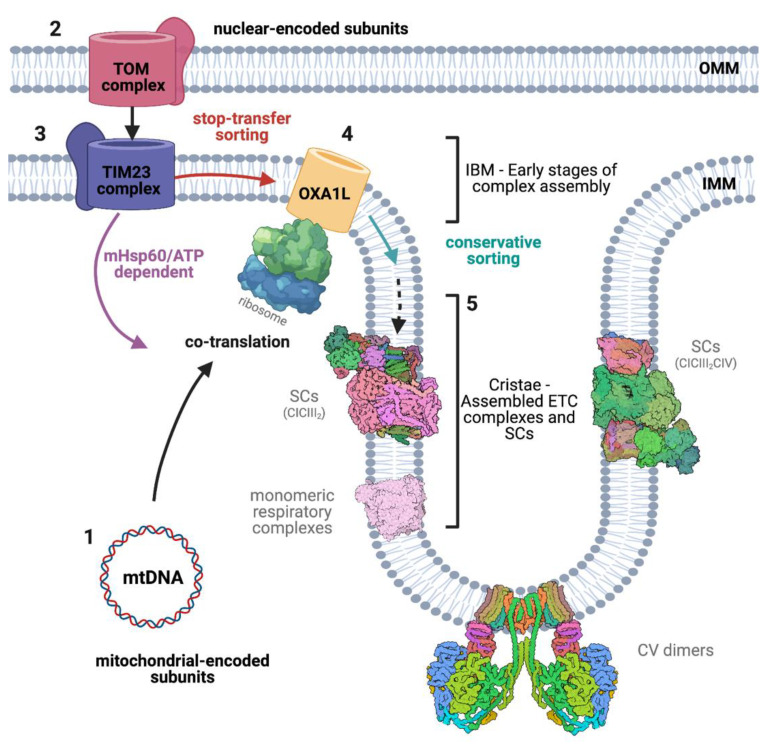
Spatial orchestration of mitochondrial respiratory complexes import and assembly and their organisation in the IMM. ETC complexes I, III, IV, and V are composed of both mitochondrial and nuclear-encoded subunits. Transcripts from the mitochondrial genome (**1**) are co-translated by mitochondrial ribosomes (here depicted as a simple arrow for clarity) and proteins inserted in the IMM via OXA1L. These newly synthesised proteins are then assembled together with the nuclear-encoded subunits, which are imported primarily through the TOM/TIM23 (**2**,**3**) complex. Additionally, proteins carrying a hydrophobic segment downstream of the MTS are arrested in the Tim23 channel and laterally inserted into the IMM through a stop-transfer sorting mechanism acquiring a N_in_/C_out_ topology. Proteins with a N_out_/C_in_ topology are instead fully imported and inserted into the IMM from the matrix side through a process known as conservative sorting, involving OXA1L (**4**). The import and insertion of these subunits in the IMM take place predominantly in IBM, a section of the IMM that runs parallel to the OMM. Then, the ETC subunits undergo a series of post-translational modifications and are incorporated in a nascent enzyme, often due to the interaction with assembly factors or chaperons. This process can occur in the monomeric enzymes and/or in the high-order SCs. Fully assembled enzymes and SCs are enriched in the cristae region of the IMM (**5**). Note: the size of monomeric respiratory complexes, supercomplexes, import machineries, and ribosomes are not to scale.

As mentioned earlier, the OXPHOS machinery is composed of both nuclear and mitochondrial-encoded subunits, requiring the synchronisation of a series of pathways and cellular machineries (Figure 2). Firstly, nuclear and mitochondrial gene expression must be coordinated. This process has been observed in yeast [141], but the underlying mechanisms are still poorly understood. It is believed that the translation of mtDNA-encoded mRNAs is regulated by a series of translational activators acting on the 5′-UTR, while other translational activators could interact with ribosomes or play a role in transcript stabilisation [142,143,144,145]. Moreover, feedback regulation mechanisms linking respiratory complex subunits’ expression with the state of complexes assembly have been described for CIII [146,147], CIV [148,149,150,151], and CV [152,153].

Interestingly, the route of import can vary for different OXPHOS subunits as well as for assembly factors (Table S1). It has long been known that the mRNA encoding nuclear proteins targeted to mitochondria can form polysomes with several ribosomes and localise to the surface of the OMM where it is translated and imported, a phenomenon known as co-translation [154,155,156]. This mechanism, observed for example for the CV subunit *ATP2* in yeast [50], is thought to promote import and assembly efficiency and requires specific nucleotide signals in the mRNA 3′-UTRs in addition to the MTS [50,157]. However, other ETC subunits, such as CIV COX4 [158], are encoded in a different type of polysomes, known as ‘free polysomes’, which are not attached to the organelle membrane [51,159,160,161,162]. Moreover, detailed observations revealed that evolutionary ancient proteins are mainly synthesised at the mitochondrial surface, the core subunits (bacterial orthologs), or proteins involved in the synthesis of metal and heme co-factors, while eukaryotic-specific supernumerary subunits are more likely to be produced in free polysomes [163].

Quantitative immunogold electron microscopy studies in both isolated mammalian mitochondria and yeast cells showed that fully assembled Complexes I-IV and SC are found preferentially in cristae membranes [164,165]. However, it has been observed that complexes intermediates might localise in specific regions of the IMM during different maturation stages [166]. Therefore, respiratory complex assembly requires the temporal and spatial coordination of two independent protein synthesis machineries. In this regard, it was initially proposed that mitochondrial-encoded subunits translated in the matrix are inserted into the cristae membrane and that imported nuclear-encoded subunits are primarily inserted in the inner boundary membrane (IBM) for later incorporation in the nascent enzymes [165].

Using super-resolution microscopy and quantitative cryo-immunogold-EM a group of authors addressed this issue in yeast and concluded that although mature CIII, CIV, and CV localise mainly in the cristae, the early stages of assembly are enriched in the IBM [166]. Nonetheless, the complete assembly pathway of CV appears to develop specifically at IMM invaginations [166]. Mature CV is known to reside at the tip of these invaginations and to play a role in membrane curvature and cristae organisation [167,168].

Lastly, ETC subunits can undergo a series of post-translational modifications, in particular cleavages and insertion of prosthetic groups, such as heme groups, copper centres, and iron/sulphur (Fe/S) clusters, that are incorporated in the nascent enzymes, as extensively reviewed [169,170,171]. The insertion of these subunits occurs in a precise order and might require the involvement of assembly factors, as described more in detail in the following sections.

#### 3.1.1. CI Assembly

**Figure 3:**
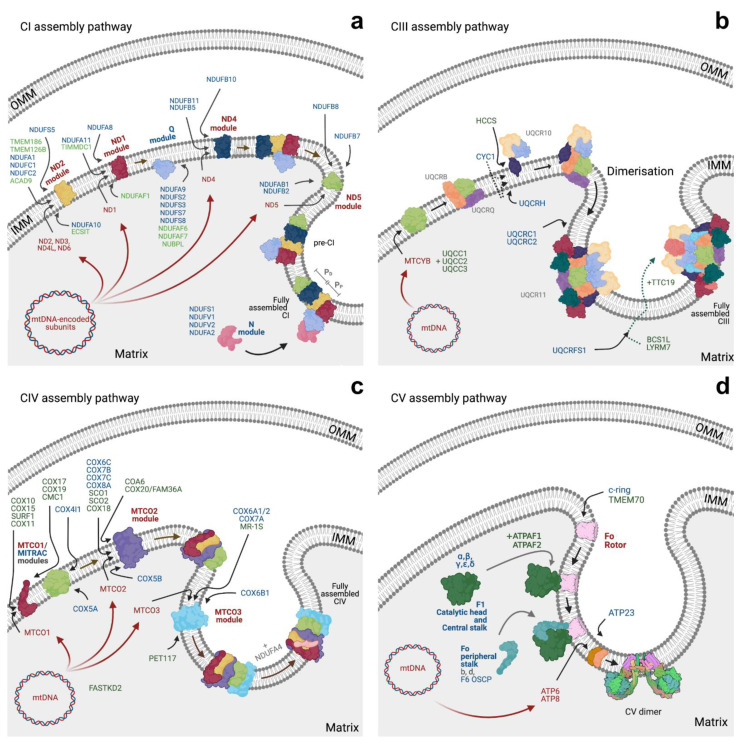
Assembly pathways for the different respiratory complexes. For the purpose of simplification, only a small portion of subunits and assembly factors are depicted in the figure, namely those for which the import route is known or expected (see Supplementary Table S1). For detailed view, please see [174]. Panel (**a**)-Complex I (CI) assembly pathway is modular and takes place in the IMM. After the synthesis, import, and maturation of both the mitochondrial- and the nuclear-encoded subunits, six subassemblies are independently formed: ND2, ND1, ND4, ND5, Q, and N-module. The preassembled modules associate with each other in a precise order. Initially, the central structure of the enzyme is formed, starting from the ND2-module and continuing with the subsequential addition of the ND1, Q, and then ND4 modules. Secondly, the extremities of the enzyme are incorporated, starting from the ND5-module, the last part of the membrane domain of CI, and finishing with the N-module, the FMN-containing intermediate that binds NADH and completes the assembly of the functional enzyme. mtDNA-encoded subunits (NDs) are indicated in red, while modules containing only nuclear-encoded subunits are indicated in blue. Panel (**b**)-Complex III (CIII) assembly starts with the maturation and insertion in the IMM of the single mitochondrial-encoded subunit, cytochrome *b* (MTCYB). The remaining nine subunits (in blue or gray, for those with known and unknown import routes) are incorporated on top of this ‘seed’, following a precise order. For CYC1, the two debated import routes are shown as dashed line. Few assembly factors (in green) are known for CIII and are involved in the maturation of MTCYB and the Rieske protein (UQCRFS1). Different assembly factors are also shown in green. Mature CIII dimerisation is required for full activity and competence. Panel (**c**)-Complex IV (CIV) assembly is modular and is initiated by the parallel formation of the MTCO1 and of the COX4/COX5A modules. The MTCO1 module associates with a variety of assembly factors including Tim21, forming the MITRAC complex. One of the last subunits to be added is NDUFA4, which was initially misattributed to Complex I. Structural subunits are shown in red/blue and assembly factors in green colour. Panel (**d**)-Complex V (CV) is comprised of three modules: F1, Fo, and the peripheral stalk. The mtDNA subunits ATP6 and ATP8 (in red) together with other nuclear-encoded subunits (in blue), including the c-ring, form the Fo domain inserted in the IMM. The F1 domain is the matrix-facing part of the enzyme. The peripheral stalk is important for the stability of the complex and also contains key subunits required for the dimerisation of mature CV.

Complex I (CI) is composed of 44 different subunits in mammals [172], organised in three structural domains: the *P-module*, inserted in the IMM, and the *N-* and *Q-modules*, protruding into the mitochondrial matrix (Figure 3a and Table S1). While the N- and Q-modules are formed exclusively by nuclear-encoded subunits, the P-module contains seven mitochondrial-encoded proteins (NDs; Figure 3a) [173]. CI assembly requires the formation of six independent modules, N, Q, ND1/P_P_-a, ND2/P_P_-b, ND4/P_D-a_, and ND5/P_D-b_, and the incorporation of each of them in a specific order [174]. The ND2 module is generated first [137] and is stabilised by its interaction with numerous assembly factors: ACAD9, ECSIT, TMEM126B, NDUFAF1, COA1, and the putative assembly factor TMEM186, which form the mitochondrial CI intermediate assembly (MCIA) [175,176]. Then, ND3, ND6, and ND4L are added to this intermediate. At least two of these assembly factors, namely TMEM126B and NDUFAF1, have been recently reported to be imported in a TIM22-dependent manner [111].

In parallel, a Q-module intermediate starts emerging through the binding of the assembly factor TIMMDC1 and the subunits ND1, NDUFA3, NDUFA8, and NDUFA13 (Figure 3a) [137]. In the intermediate phase of CI assembly, the ND4 module is formed, involving the assembly factors FOXRED1, ATP5SL, and TMEM70, followed by the ND5 module, the distal extremity of the membrane arm, which binds the assembly factor DMAC1/TMEM261 [177]. Finally, the N-module, composed of NDUFV1, NDUFV2, NDUFS1, and NDUFA2, is incorporated, generating the functional enzyme (Figure 3a).

It is worth noting that all mtDNA-enconded subunits, i.e., the NDs, are inserted in the IMM via OXA1. Still, most CI subunits are synthesised in the cytosol and then targeted to mitochondria by their N-terminal MTS, and thus preferentially use the TIM23 route [178]. Contrarily, NDUFS5, NDUFB7, NDUFB10, and NDUFA8 have been shown to be imported to the IMS using the MIA pathway [179,180]. The remaining 12 subunits (NDUFA5, NDUFS5, NDUFC2, NDUFB10, NDUFB6, NDUFB9, NDUFB3, NDUFA3, NDUFA8, NDUFA13, NDUFB1, NDUFB4) [177] and three CI assembly factors (TMEM126B, FOXRED1, and TIMMDC1) [181] do not contain a cleavable MTS and are imported into the organelle as a result of uncharacterised internal signals. Notwithstanding, it has been recently demonstrated that NDUFB10, TIMMDC1, and TMEM126B are imported via TIM22 [111], possibly suggesting a similar import route for this class of proteins that requires further investigation. This pathway is also used by NDUFA11, a supernumerary subunit of CI that is conspicuously located at the interface between CI and CIII in SCs. Interestingly, TIMMDC1 and NDUFA11 belong to the Tim17 family [182], providing another link between protein import and respiratory/SC assembly and function. A direct involvement can be observed in plants where in this case the NDUFA11 homolog B14.7 is directly associated with TIM23 complex [183].

Once imported, several core subunits need further maturation and insertion of prosthetic groups, such as Fe/S clusters. To date, only one assembly factor is known to be involved in the incorporation of 4Fe/4S clusters in the peripheral arm: NUBPL, a member of the Mrp/NBP35 ATP-binding proteins family [184,185]. However, it is expected that several other unidentified proteins play a role in this process.

#### 3.1.2. CII Assembly

CII subunits (SDHA-D) are all nuclear-encoded and imported into mitochondria post-translationally. The hydrophilic membrane domain (SDHC and SDHD) contains a heme *b* group and two ubiquinone binding sites [186] and forms an intermediate subcomplex [187]. In contrast, the mature forms of SDHA and SDHB are produced and inserted independently.

A flavine adenine dinucleotide (FAD) cofactor is inserted in SDHA via the interaction with the assembly factor SDHAF2/Sdh5 [188], while SDHAF1, assisted by SDHAF3, promotes the insertion of SDHB Fe/S clusters ([2Fe-2S], [4Fe-4S], and [3Fe-4S]) [189,190,191,192].

Interestingly, the yeast ortholog of SDHC, Sdh3, was found to form a subcomplex with Tim18 and participate in the biogenesis and assembly of the TIM22 complex [193]. However, Tim18 arose from duplication of the Sdh3 gene and does not have an ortholog in mammals. Similarly, the mammalian SDHC subunit has never been detected interacting with the TIM22 complex, suggestive of divergent mechanisms for the formation of the translocase between the two organisms [98], as discussed in the previous section.

#### 3.1.3. CIII Assembly

Complex III (CIII) is composed of 10 subunits in both yeast and mammals. All CIII subunits are encoded by nuclear DNA except cytochrome *b* [194,195].

CIII assembly (Figure 3b) begins with the synthesis and insertion of the mtDNA-encoded subunit cytochrome *b* in the IMM, in both yeast and in mammals. The insertion of the yeast subunit has been shown to occur via Oxa1 [126], while in mammals, the depletion of OXA1L only marginally affects the biogenesis and function of the enzyme, suggesting a possible alternative route or compensatory mechanisms [196]. In yeast, this process is highly coordinated with the synthesis of nuclear-encoded proteins as a result of translational activators that regulate the expression of mitochondrial genes and their own expression in relation to mitochondrial respiration [197]; however, the same mechanism has not been observed in mammals yet. Four translational activators are known to be involved in the stability and translation of cytochrome *b* mRNA: Cbp1, Cbs1, Cbs2, and the complex Cbp3/Cbp6 [198]. However, only three factors are known in mammals, the ubiquinol–cytochrome *c* reductase complex assembly factors 1 and 2 (UQCC1 and UQCC2) [199], orthologs of Cbp3/Cbp6, and UQCC3, ortholog of Cbp4 (Table S1) [200].

In both yeast and mammals, the second step of CIII maturation involves the insertion of the subunits Qcr7 and Qcr8 (UQCRB and UQCRQ in mammals) [201], whilst the following proceedings differ between the two model organisms. In yeast, for example, an independent subassembly module containing the two large structural core subunits, Cor1 and Cor2, and the catalytic subunit cytochrome *c1* is formed [202]. This intermediate is then incorporated into the nascent enzyme together with the Qcr6 subunit [202,203,204]. In humans, however, CYC1 forms sub-assemblies with UQCR10 and potentially UQCRH (Qcr6 in yeast), without interacting directly with the core proteins UQCRC1 and UQCRC2 [131]. Moreover, this intermediate can be found in association with CIV subunits [131], suggesting that CIII might use CIV or modules of CIV as a structural scaffold during biogenesis. An alternative hypothesis is that CIII intermediates sequester CIV subunits when SC formation is impaired [131]. Importantly, it has been shown that dimerisation of two nascent CIII occurs during this stage [205].

During the intermediate assembly process, another player is added to the CIII complex. Mature Cyt1 contains a single heme centre and is anchored to the IMM via a single transmembrane segment near its C-terminus with its mature N-terminus soluble in the cytosol [206]. The full mechanism for the insertion and maturation of this atypical topology of an MTS-containing protein is still under debate. Nonetheless, both models share the initial steps with the Cyt1 precursor being translated in the cytosol and imported via TOM/TIM23 complexes into the mitochondria, where its N-terminal bipartite presequence is cleaved into two independent processes [207]. According to one proposed mechanism, the whole protein is fully imported into the mitochondrial matrix, and only after the first cleavage is performed, the hydrophobic sequence is re-located into the membrane, allowing the second proteolytic cleavage in the IMS [208]. However, earlier studies showed that depletion of matrix ATP has no impact on the import and maturation of Cyt1, suggesting that the precursor does not cross the IMM completely during translocation [209]. Therefore, the second model suggests that although the positively charged portion of the MTS reaches the mitochondrial matrix, the internal hydrophobic signal halts import, allowing for the lateral release (stop-transfer) of Cyt1 from the TIM23 channel and its insertion in the membrane [198]. The positive-charged MTS is then cleaved by MPP in the matrix, while the C-terminal α-helix is inserted into the IMM and the heme group is subsequently added to the protein by the holocytochrome *c*_1_ synthetase (Cyt2 or HCCS1 in mammals) [210]. This modification leads to a conformational change that exposes the second hydrophobic targeting sequence for cleavage by Imp2, leaving the N-terminal of the mature Cyt1 soluble in the IMS [211].

The last stages of CIII assembly involve the incorporation of Qcr9 (UQCR10 in mammals), Qcr10 (UQCR11), and Rip1 (UQCRFS1) [212,213]. Once more, minor differences in the import of the nuclear-encoded Rip1/UQCRFS1 subunit exist between yeast and mammals. In yeast, the subunit is imported into the matrix via the TOM/TIM23 route [214] and subsequently cleaved by matrix proteases, MPP and mitochondrial intermediate peptidase (MIP), for complete removal of the MTS [215]. Next, Rip1 is translocated back across the IMM to the IMS, where it is incorporated in the complex. In this regard, Bcs1 is thought to be involved in the export of the Rieske Fe/S domain from the matrix into the IMS [216], since it is able to recognise the correctly folded Rieske protein and act as a protein translocase. In fact, cryo-EM structures of Bcs1 in yeast [217] and mouse [218] suggest the formation of an airlock-like mechanism for Rip1/UQCRFS1 translocation. Conversely, Mzm1 (LYRM7 or MZM1L in humans) stabilises Rip1 in the matrix before translocation to the IMS [219,220].

In contrast to yeast, the mammalian UQCRFS1 N-terminal import signal is not cleaved during import but rather after successful incorporation of the subunit, and in a single cleavage step. Importantly, the cleaved segment remains attached to the enzyme as an extra subunit [221]. Then, mammal-specific assembly factor TTC19 binds to fully assembled CIII for clearance of UQCRFS1 fragments, converting it to a fully functional and competent respiratory complex [222,223].

#### 3.1.4. CIV Assembly

Mammalian CIV is composed of 14 subunits, 11 of which are nuclear-encoded and the remaining three are encoded by mtDNA (MTCO1, MTCO2, and MTCO3) [224]. Similar to CI, CIV assembly also occurs in a modular fashion (Figure 3c), and the first subassembly structure formed during CIV biogenesis contains two nuclear-encoded subunits, COX4I1 and COX5A [225], as well as an assembly factor, HIGD1A [226].

Next, the MTCO1 module, also known as ‘MITRAC’ (MItochondrial Translation Regulation Assembly intermediate of Cytochrome *c* oxidase) [227,228], is generated. This subassembly is composed of the mitochondrial-encoded subunit MTCO1 and a series of assembly factors necessary for its insertion into the IMM (including COX14/C12ORF62, COA3/CCDC56/MITRAC12, and OXA1 [126,227,229,230,231]) and for its maturation (including COX10, COX15, and SURF1 involved in the heme group biosynthesis and insertion [145,232,233] and COX11, COX17, and COX19 involved in the incorporation of the CuB group [234,235,236]). Interestingly, Tim21, a subunit of TIM23, was found to be associated with the MITRAC complex and seems to shuttle imported CIV subunits from the TIM23 translocase to the nascent enzyme [227]. Tim21 was also suggested to play a possible role in CI biogenesis [227].

Following the formation of MITRAC, the next assembly step requires the incorporation of the MTCO2 module (Figure 3c). The mtDNA-encoded subunit MTCO2 is inserted into the IMM via OXA1L together with the assembly factors COX18, COX20/FAM36A, and TMEM177, which are required for the export of MTCO2 C-terminal domain [237,238,239]. Afterwards, the copper-binding proteins COX17, SCO1, and SCO2 [240,241,242] together with COA6 [243,244] and COX16 help with the insertion of the Cu_A_ centre [245,246]. In the meantime, the nuclear-encoded subunits that form this module (COX5B, COX6C, COX7C, COX8A, and, most probably, COX7B) are incorporated. Finally, the MTCO3 module (MTCO3, COX6A1, COX6B1, COX7A2) is formed and added to the nascent enzyme [225], followed by NDUFA4, initially described as a CI subunit and later assigned to CIV [145].

#### 3.1.5. CV Assembly

Complex V (CV), also known as ATP synthase, is organised in two domains: the entirely nuclear-encoded F_1_ domain facing the mitochondrial matrix, and the F_o_ domain embedded in the IMM, containing both nuclear- and mtDNA-encoded subunits, namely ATP6 and ATP8 [247,248].

The formation of CV occurs through three independent sub-assembly steps (Figure 3d) [249]. First, the F1 subcomplex is formed through the interaction of chaperones ATPAF1/ATP11 and ATPAF2/ATP12 with the subunits ATP5B and ATP5A1, respectively [250]. The c-ring module is then assembled independently via mechanisms that remain unclear. Finally, the peripheral stalk is incorporated in two steps: first there is the inclusion of b/ATP5F1, d/ATPH, F6/ATP5J, and OSCP/ATP5O and then the addition of e/ATP5I, g/ATP5L, and f/ATPJ2 [251,252].

Interestingly, the mammalian c-subunit is encoded by three nuclear genes (*ATP5G1*, *ATP5G2*, and *ATP5G3)*, which differ in their cleavable N-terminal targeting sequences but give rise to identical mature proteins [253,254]. The mechanisms behind the import and insertion of mammalian c-subunit(s) in the IMM are still unclear. However, studies in *Neurospora crassa* suggest that the MTS and the first transmembrane region could be initially translocated to the matrix via the TIM23 complex. Then, following the removal of the presequence, the transmembrane domain would be inserted into the membrane and the N-terminus exported to the IMS [125]. In bacteria, the insertase YidC, homolog of OXA1, facilitates the membrane insertion of the c-subunit [255]. The second transmembrane domain, instead, might be imported in a follow up step through a stop-transfer mechanism via the TIM23 complex as previously described for Cox2 in plants [256]. Interestingly, two assembly factors previously known for being involved in CI assembly, TMEM70 and TMEM242, were found acting as a scaffold for c-ring assembly [257,258].

## 4. Pathologies with Underlying Mitochondrial Import Defects

As mentioned earlier, TIM23 is responsible for the import of the vast majority of matrix and IMM proteins. Therefore, it is no surprise that TIM23 knockout in mice is embryonic lethal, even prior to implantation [259]. Similarly, TIM23 haploinsufficiency displays neurological defects and premature aging phenotype, further demonstrating the importance of protein import to maintain mitochondrial function and body health [259].

However, there are other occasions where the dysfunction might result from precursors clogging the channel or impaired ETC unable to provide driving force energy, rather than issues with the translocase *per se*. The cell has developed methods to detect and try to repair these problems, discussed in more detail in the next section. However, whenever this repair system fails or become overwhelmed, it creates cell and tissue stress, leading to general mitochondrial dysfunction, cytosolic toxicity, and disease. In regard to neurodegeneration, whilst mitochondrial dysfunction, amongst other effects, has long been recognised as a contributing factor in the pathogenic mechanisms of neurodegenerative diseases, the involvement of mitochondrial protein import, be it in a causative or consequential manner, is just beginning to emerge more recently. These defects have been summarised in Table 2.

### 4.1. Mitochondrial Diseases

Mitochondrial diseases are generally provoked by genetic mutations in complexes’ subunits or assembly factors, as extensively discussed in numerous other reviews [276,277,278,279,280]. However, defects in the machinery involved in the import of these subunits have also been identified as the cause of mitochondrial pathologies, leading to different clinical features.

Mutations in *TIMM50,* which encodes a subunit of TIM23 complex, have been associated with severe lactic acidosis and seizures, linked to defects in import of ETC proteins, alterations in SC formation and a general respiratory deficiency [281,282,283]. Biochemical analysis of a patient with impaired TIM23 complex due to compound heterozygous mutations in *TIMM50* revealed reduced steady state levels of CI, II, and IV, but interestingly not CIII and V [282]. These results suggest the possibility of alternative import routes for certain ETC subunits or different interactions with the import machinery for subunits transported into the matrix or inserted into the IMM. Another study with patients with two homozygous missense mutations in the *TIMM50* gene, however, produced opposite results, showing normal activities of Complex I–IV and defective activity of CV [281]. A possible explanation for this phenotype is that, as shown in yeast, the import of subunits 9 and β of CV is highly dependent on TIM50, whilst import of CIII subunit CYC1 and CIV subunit COX5A import is only mildly affected by TIM50 defects [66]. Finally, another subject carrying compound heterozygous mutations within the IMS domain of *TIMM50* exhibited 3-ethylglutaconic aciduria, symptoms of Leigh syndrome, and dilated hypercardiomyopathy, associated with altered mitochondrial morphology [283]. Additionally, patients also experienced a general decrease in the levels of fully assembled complexes and their activity alongside a reduction in SC formation and a drop in the maximum respiratory capacity [283].

Interestingly, defects in other TIM23 complex subunits do not result in impaired mitochondrial respiration. For example, alterations in the formation of the DDP1/TIMM8a–TIMM13 complex found in a patient with deafness/dystonia and with a *de novo* mutation in DDP1(C66W) did not lead to defects in the activity of any of the respiratory complexes [284]. More recently, another reported case of neuromuscular presentation of mitochondrial disease was found to be associated with compound heterozygous mutations in *TIMM22* [285]. However, while cellular respiration was reduced in the patient cells, no evident defect in respiratory complexes or SC assembly was found. In a different report, where TIM22 assembly and activity was impaired by the removal of AGK, a mild CI assembly defect and respiration impairment was observed [111]. In contrast to the compound heterozygous variant, this observation suggests a direct involvement of the TIM22 complex in the import of CI subunits and/or factors required for CI assembly that requires further investigation.

Regarding defects at the TOM complex level, a patient with severe anaemia, lactic acidosis, and developmental delay were identified with compound heterozygous variants in the *TOMM70* gene [286]. Interestingly, this patient presented with respiratory complex deficiencies with a primarily marked defect in CIV, including decreased steady state levels of fully assembled enzyme, activity, and a reduction in CIV-containing SC, while SCI:CIII_2_ species appeared unaffected.

Finally, defects in OXA1L, essential for the re-localisation of newly imported nuclear-encoded proteins in the matrix into the IMM, could have an impact on complexes’ assembly and activity. One patient was identified with mutations in *OXA1L* and tissue-specific combined respiratory complex deficiencies, which led to severe encephalopathy, hypotonia, and developmental delay [196]. Interestingly, skeletal muscle biopsy from this patient showed defects primarily at the CIV and CV level with only milder defects in CI despite the fact that neuropathology experiments indicated an isolated CI deficiency in the central nervous system. Although the tissue-specificity observed in this patient remains unclear, it suggests a possible differential expression of OXA1L isoforms in different tissues or the presence of alternative insertases in human mitochondria.

### 4.2. Alzheimer’s Disease

Alzheimer’s disease (AD) is the most commonly occurring form of neurodegeneration, and growing evidence is linking it to mitochondrial dysfunction at all levels of AD neuropathology. AD is characterised by the death or loss of neurons in specific, susceptible areas of the brain, as well as by the presence of two pathological hallmarks: extracellular senile plaques and neurofibrillary tangles (NFTs) [287].

Senile plaques are deposits of accumulated amyloid-beta peptide (Aβ), a 40-42 amino acid peptide that is produced by specific, sequential proteolytic cleavages of amyloid precursor protein (APP). The biology of APP processing and its relevance in AD is reviewed in great detail in a previous review [287]. In a study carried out in mitochondria from human AD brains, APP has been found to accumulate in the TOM40 channel, forming a stable complex of ~480 kDa (Table 2) [260]. It also accumulates with both TOM40 and TIM23 to form a supercomplex of ~620 kDa [260]. Interestingly, mitochondrial APP levels varied both among patients, corresponding to the severity of AD, as well as across brain regions, with higher levels displayed in the regions of the brain that are more vulnerable to AD: the cortex, hippocampus, and amygdala [260]. Furthermore, the levels of APP accumulation in the mitochondria of AD brains directly correlates with mitochondrial dysfunction [260], suggesting that APP-mitochondrial translocase complex formation and aggregation may in fact be a causative factor in AD progression.

Furthermore, a study in PC12 cells showed that chronic, sub-lethal Aβ exposure induces a significant reduction in mitochondrial protein import, and that this, when sustained over long periods, leads to mitochondrial dysfunction highlighted by a reduction in Δψ, altered mitochondrial morphology, and increased ROS production (Table 2) [261]. This consequential negative impact on mitochondrial function is likely due to the loss of important proteins that are usually imported via TOM40, such as proteins necessary for respiratory complex activity and assembly, as well as ROS scavenging proteins.

The second characteristic hallmark of AD, neurofibrillary tangles, insoluble aggregations made up primarily of hyperphosphorylated Tau protein, is a symptom of not only AD, but of all tauopathies. Cell line studies have shown that various forms of aggregation-prone Tau (wildtype, hyperphosphorylated, or caspase cleaved N-terminal fragment) are imported into mitochondria and localised to the IMS and OMM (Table 2) [262,263]. Whilst, to the best of our knowledge, no studies have specifically looked at the impact of Tau on mitochondrial protein import efficiency, the body of evidence highlighting Tau accumulation in mitochondria suggests this would be worth investigating.

### 4.3. Parkinson’s Disease

Parkinson’s disease (PD) is very closely associated with mitochondrial dysfunction, owing to consistent evidence suggesting reductions in CI activity in PD patient brains and other tissues [288,289], in addition to genetic links between familial PD and mitochondrial dysfunction [290]. These well characterised mitochondrial abnormalities in PD and potential therapeutic strategies to target them have been reviewed extensively previously [291].

Lewy bodies, which form in the *SN,* are the main pathological hallmark of PD and are made up mainly of aggregated alpha-synuclein (α-syn), an abundant presynaptic molecule [292,293]. Alpha-synuclein is a 140 amino acid molecule, which is thought to play a role in neuronal plasticity and synaptic function [292,294,295]. The aggregation of α-syn is highly neurotoxic, and studies of transgenic mice overexpressing α-syn have shown that its accumulation can lead to a PD-like phenotype, consisting of the formation of prominent intraneuronal inclusion bodies, loss of dopamine neuron terminals, and motor deficits [296]. Intriguingly, much evidence has suggested that neuronal injury caused by α-syn may be mediated by mitochondrial dysfunction and degeneration [264,266,297,298,299,300,301].

Multiple studies have shown that α-syn localises to, and accumulates within, mitochondria (Table 2) [264,266,300,301]. This is thought to be mediated by a cryptic, non-canonical MTS within the N-terminal 32 amino acids of α-syn [266]. The transport of α-syn into mitochondria does not occur in the presence of oligomycin, which inhibits ATP synthase and thus depletes mitochondrial ATP, or, carbonyl cyanide-*m*-lorophenylhydrazone (CCCP), which disrupts the mitochondrial Δψ, highlighting that its import is dependent on both ATP and Δψ, consistent with the import requirements for known mitochondrial proteins [266]. The A53T point mutation that occurs in rare familial PD cases is also imported into mitochondria, but with significantly higher efficiency than the wildtype protein [266], which may account for the faster development of cellular abnormalities seen in cells expressing the A53T version of α-syn compared to the wildtype [265].

It has been previously shown by electron microscopy that the majority of mitochondrial α-syn accumulates at the IMM and that it interacts with CI [266]. This causes a significant reduction in CI activity, as well as an increase in ROS production, inducing oxidative stress [266], which may account for some of the toxic effects on dopaminergic neurons. Importantly, α-syn lacking the N-terminal MTS failed to localise to mitochondria and did not exhibit any of the mitochondrial dysfunctions seen in the wildtype [266].

A study carried out in cell models of PD showed that in vitro treatment with rotenone leads to an increase in S129 phosphorylation of α-syn [267]. The resulting post-translationally modified α-syn species were observed to bind with high affinity to TOM20 molecules, leading to a loss of the critical interaction between TOM20 and TOM22 (Table 2) [267]. Consequently, mitochondria have impaired protein import and widespread mitochondrial dysfunction, displayed by a loss of Δψ, reduced respiratory capacity, and increased oxidative stress in SH-SY5Y cells [267]. This α-syn/ TOM20 interaction and subsequent loss of import were also detected in the dopaminergic neurons from the *SN* of post-mortem brains of PD patients [267]. The authors highlighted mechanisms for rescuing this disorder, namely by in vivo knockdown of endogenous α-syn and by in vitro TOM20 overexpression, both of which preserve mitochondrial import and thus present potential therapeutic strategies for further investigation [267,302].

It has been shown that the core component of the TOM complex, TOM40, is downregulated in the midbrain of PD patients as well as in α-syn transgenic mice (Table 2) [268]. Importantly, levels of TOM20 remained the same, suggesting that this is a specific effect of TOM40, rather than a general reduction in mitochondrial proteins. Furthermore, this reduction in TOM40 levels corresponded with α-syn accumulation in PD brains, inferring a further functional link between α-syn aggregation and mitochondrial import dysfunction [268].

A recent study showed that, in addition to the key roles in mitochondrial quality control and biogenesis already established [303,304,305,306,307,308], Parkin, an E3 ubiquitin ligase, also plays a part in stimulating mitochondrial protein import, whilst stimulation of import is not achieved by disease-causing Parkin variants (Table 2) [27]. Furthermore, the results of this study showed that this effect relies on PINK1-mediated Parkin activation and results in ubiquitylation of TOM40 subunits, as well as an increase in K11 ubiquitin chains on mitochondria [27]. The importance of PINK1-Parkin regulation of mitochondrial import is highlighted by data showing excessively low levels of mitochondrial import in cells from PINK1- and PARK2-linked PD patients. This effect may be reversed by phosphomimetic ubiquitin in cells with residual Parkin activity, probably by bypassing the need for PINK1-dependent Parkin activation or by enhancing Parkin activity [27].

### 4.4. Huntington’s Disease

Huntington’s disease (HD) is an autosomal dominant neurological disorder characterised by neuronal loss in the striatal and cortical regions of the brain. The genetic cause of HD is an abnormal expansion of polyglutamine repeats (encoded by the CAG codon) in the huntingtin gene (HTT) [309].

N-terminal fragments of variant Huntingtin proteins, which form cytotoxic aggregates [310,311], have been shown to interact directly with mitochondria in cell and mouse models of HD (Table 2) [312,313]. Furthermore, a study showed that the variant Huntingtin localises to mitochondria from human HD brains isolated mitochondria, and that it directly interacts with the TIM23 complex, inhibiting import as a result (Table 2) [269]. These import defects were consistent in primary neurons expressing Huntingtin variant as well as in forebrain synaptosomal mitochondria in HD mice at early stages of the disease [269]. Notably, these import defects were not found in liver mitochondria from the same mice, suggesting that the import defects are specific to neurons [269]. Additionally, the inhibition of import preceded mitochondrial respiratory dysfunction and acted as a trigger for cell death, which was rescued upon augmentation of mitochondrial import by overexpression of TIM23 complex subunits, highlighting this pathway as a potential therapeutic strategy against HD [269].

Considering the early detection of impaired import in HD mice [269], it suggests that import defects precede the other mitochondrial insults described in HD models, namely decreased Δψ [314], reduced respiratory capacity and ATP levels [315,316], defective calcium buffering function [317], and altered mitochondrial morphology and number [318]. A plausible explanation is that inhibition of import would prevent key respiratory complex proteins from being imported and carrying out their functions, resulting in widespread mitochondrial damage.

Mutant Huntingtin has been linked to dysfunctions in the MIA pathway (Table 2) [270]. In neuronal cell lines, the expression of proteins of the MIA pathway were found to be significantly different to levels in control cells [270]. More specifically, ALR and CHCHD4 levels were reduced, and the ratio altered compared to control cells, whilst cytochrome *c* levels were increased, compared to the control group. Proteins that require the MIA pathway for import also displayed reduced expression levels, whilst CIV proteins not imported via this route, such as MTCO3, were unchanged, highlighting that this effect is specific to MIA substrates rather than a CIV effect [270]. In cells with a homozygous variant, however, levels of MTCO3 were also reduced [270], suggesting that there may be some CIV assembly defects. The observed effects on the MIA pathway were accompanied by deficient respiration, alterations in mtDNA, and changes in mitochondrial morphology [270]. These effects are consistent with what has been shown previously in both HD models and MIA deficient models [319,320,321,322,323,324].

### 4.5. Amyotrophic Lateral Sclerosis

Amyotrophic lateral sclerosis (ALS) is a rare motor neuron disease, strongly associated with mutations in *SOD1*, a ROS scavenging enzyme [325,326]. Characteristic features of mitochondrial dysfunction have been observed across ALS patients, and respiratory chain impairment has been highlighted as a common feature in the muscles of ALS patients, even prior to neuronal deficits being found [327,328,329]. This finding is consistent across both patient samples and experimental model systems and has highlighted mitochondrial dysfunction as a major pathological feature in ALS [330].

A small proportion of wildtype SOD1 is known to localise to the IMS under physiological conditions in both yeast and mammals [331,332]. Its antioxidant role in detoxifying ROS species produced by the ETC (mainly CI and CIII) is well established [332,333]. Disease associated variants of SOD1, however, have been shown to accumulate not only in the IMS but also within the matrix and the OMM, where it aggregates and interacts with OMM proteins (Table 2) [272,273]. This mislocalisation of SOD1 variants lead to excessive ROS production and subsequent mitochondrial dysfunction and toxic effects on the cells, which can be rescued by selective targeting of wildtype SOD1 to the IMS [271]. Evidence also shows alterations in activity of the respiratory complexes and in mitochondrial calcium buffering capacity associated with disease-causing SOD1 variants [317,334].

A proteomic screen of protein level changes in mitochondria from rat spinal cord of ALS-linked variant SOD1^G93A^ showed vast changes in mitochondrial import and CI related proteins compared to SOD1^WT^ mitochondria (Table 2) [274]. Levels of TOM subunits TOM20, TOM22, and TOM40 were increased in the affected mitochondria although, surprisingly, in vitro import assays highlighted a 30% reduction in protein import levels in these mitochondria compared to wildtype [274].

Furthermore, variants of mitochondrial IMS protein CHCHD10, which is crucial for cristae remodelling, have been linked to progression of ALS as well as frontotemporal dementia [275]. The native version of this protein is imported via the MIA pathway, where disulphide bonds are formed within the CHCHD of the protein [275]. A novel CHCHD10 variant, Q108P, discovered in a patient with rapidly progressing ALS, has been shown to almost completely abolish its import, resulting in reduced mitochondrial respiratory capacity, an effect that is rescued by overexpression of CHCHD4 (Table 2) [335]. Interestingly, the C9orf72 protein, which is often mutated in cases of ALS and frontotemporal dementia, has recently been shown to be an IMM protein vital for the assembly and stabilisation of CI, and its translocation occurs via the MIA pathway [336]. These studies demonstrate the importance of mitochondrial protein import and proper respiratory function in the prevention of motor neuron diseases such as ALS, highlighting import pathways as interesting potential targets for treatment.

## 5. Repair Pathways

In order to maintain the integrity and function of the mitochondria, a complex hierarchy of quality control mechanisms exists. This consists of repair mechanisms at the molecular, organelle, and cellular levels via a plethora of complex systems including mitochondrial chaperones and proteases, mitochondrial dynamics and distribution, mitochondrial-derived vesicles (MDVs), mitophagy, and apoptosis [337]. In addition to the emergence of links between mitochondrial import defects and neurodegenerative diseases, there is also evidence implicating stress response pathways in neurodegeneration. This evidence suggests that the pathways may have either a protective or exacerbating role in disease progression in different models. This section will discuss some of the stress response pathways that cells have developed in response to mitochondrial dysfunction for restoration of mitochondrial import function, respiratory capacity, and mitochondrial and cytosolic proteostasis.

### 5.1. UPR^mt^

The mitochondrial unfolded protein response (UPR^mt^) is known to be directly activated in response to impaired proteostasis in the mitochondrial matrix and has been extensively studied in *Caenorhabditis elegans*, where it was first identified [338]. The UPR^mt^ is a transcriptional response pathway that eliminates proteotoxic stress and fine-tunes mitochondrial respiration [339,340].

The sensor for this pathway is stress activated transcription factor (ATFS-1, ATF5 in mammals), which contains both a weak N-terminal MTS and a strong C-terminal nuclear localisation sequence (NLS) [341]. Proteotoxic mitochondrial stress, caused by a variety of mitochondrial stressors including: impairment of the import machinery (*timm23* or *tomm40*(RNAi) or paraquat application via CI inhibition), loss of ETC quality control (*spg-7*(RNAi)), or mtDNA depletion (ethidium bromide application) [341], results in retargeting of ATFS-1 primarily to the nucleus. There, ATFS-1 acts with transcriptional regulators DVE-1 and UBL-5 to induce the production of mitochondrial chaperone proteins HSP-6 and HSP-60, as well as proteases CLPP-1, LONP-1, SPG-7, and YMEL-1, metabolic genes GPD-2 and SKN-1, and core component of the TIM23 complex, TIM17 [339,342,343]. ATFS-1 is also responsible for repressing the expression of ETC genes, thus shifting expression capacity to increase mitochondrial protein folding and reducing the proteotoxic stress from mistargeted proteins in the cytosol [344].

Importantly, the localisation of ATFS-1 is mediated by HAF-1, the previously identified UPR^mt^ regulator and general attenuator of mitochondrial protein import during stress [345]. In the absence of HAF-1, ATFS-1 is unable to transition to the nucleus under stress conditions, thus failing to activate the UPR^mt^ [341,345]. It is important to note that ATFS-1 has a relatively weak MTS, meaning that minor effects on mitochondrial protein import efficiency, such as partially depolarised mitochondria, can trigger the stress response pathway, even though some mitochondrial proteins with stronger targeting sequences may still be imported successfully under these conditions [346].

In mammalian cells, the UPR^mt^ is thought to act in a similar way to that described above for *C. elegans*, where transcription factor ATF5 is regulated and triggers a stress response very similar to that described for the *C. elegans* homolog ATFS-1 [347]. However, studies have shown that integrated stress response (ISR) factor ATF4 is also involved in the transcriptional reprogramming of the mammalian UPR^mt^ [348,349]. It is also thought that the heat shock response (HSR) is activated alongside the UPR^mt^ in what is known as the mitochondrial to cytosolic stress response (MCSR) [338]. The HSR is activated by dysfunctional ETC activity or complex assembly and restores cytosolic proteostasis via transcription factor HSF-1 [350].

Given the vast mitochondrial dysfunction described in neurodegeneration, it is not surprising that there is an emerging body of evidence linking the UPR^mt^ to neurodegeneration. In PD, variants of *C. elegans* PINK1 and Parkin orthologs PINK-1 and PDR-1 lead to increased activation of the UPR^mt^, which mitigates mitochondrial dysfunction caused by the corresponding mutations, subsequently increasing dopaminergic neuron survival [351]. However, a study in *C. elegans* showed that prolonged UPR^mt^ activation can in fact exacerbate mitochondrial dysfunction and dopaminergic cell death by favouring retention of dysfunctional mitochondria [352], which is important to note given the long-term and progressive nature of neurodegenerative diseases. Furthermore, the HSR has been shown to be activated in mouse and cell models of PD [353,354], and studies have also highlighted heat shock protein overexpression as an attenuator of α-syn aggregation and subsequent dopaminergic cell death [355].

In vivo studies also reveal that the accumulation of ALS SOD1 variant SOD1^G93A^ in the IMS leads to activation of the UPR^mt^ [356], consistent with other studies showing that activation of the UPR^mt^ precedes disease onset and increases throughout disease progression in ALS mutant mice [357]. Similarly, in AD, accumulation of Aβ has been shown to activate the UPR^mt^ [358], and there are high levels of UPR^mt^ marker genes in post-mortem brain samples from AD patients [359]. Interestingly, the inhibition of UPR^mt^ by knockdown of genes coding for key UPR^mt^ proteins HSP-6, HSP-60, and DVE-1 exacerbates AD phenotypes in *C. elegans* [360], suggesting that the UPR^mt^ may play a protective role in AD progression.

Recently, evidence has shown that an earlier form of the UPR^mt^ precedes the classical UPR^mt^, and is activated by the accumulation of unprocessed precursor proteins inside mitochondria, due to impaired processing by MPP [361]. In this case, yeast nuclear transcription factor Rox1 is relocalised to mitochondria, binding to mtDNA and regulating mtDNA transcription and translation, and maintenance of mitochondrial respiratory and import functions [361].

### 5.2. UPR^am^

The ‘UPR activated by the mistargeting of proteins’ (UPR^am^) is another major stress response pathway that responds to mitochondrial import defects via the TIM23 or MIA pathways [362]. It has been well characterised in yeast, and there is some evidence that suggests that it also takes place in mammalian cells [362,363]. In yeast, the trigger for this is not the lack of import of a sensor protein, like ATFS-1 in the UPR^mt^, but instead the accumulation of cytosolic precursor proteins [362]. This accumulation of cytosolic precursors leads to increased proteasome assembly, triggered by increased activity of proteasome assembly factors Irc25 and Poc4, and subsequent proteasomal degradation of the accumulated cytosolic precursor proteins [362]. This is accompanied by an inhibition of protein synthesis, which acts to prevent further accumulation of mistargeted proteins in the cytosol [362].

The UPR^am^ pathway is in part identical to the UPR^mt^ and is probably activated simultaneously alongside the UPR^mt^; however, they differ in that the UPR^mt^ acts by regulating the abundance of mitochondrial chaperones and proteases, whilst the UPR^am^ regulates the expression of all mitochondrial proteins, as well as activating the proteasome to clear aggregated proteins [214,364].

To the best of our knowledge, there have been no studies thus far directly implicating the UPR^am^ pathway in neurodegeneration. However, proteasomal degradation via the ubiquitin–proteasome system is known to be downregulated in the affected neurons of many neurodegenerative diseases including AD, PD, HD, and ALS, and it is thought that this is mainly caused by the accumulation of cytotoxic protein aggregates [365,366,367]. For example, in AD, aggregated, ubiquitinated Tau can block entry of unfolded proteins to the 19S catalytic subunit of the proteasome by binding to the recognition site, resulting in impaired proteasomal degradation and enhancing the accumulation of precursor proteins [368].

### 5.3. mPOS

The mitochondrial precursor over-accumulation stress (mPOS) pathway is a mechanism of mitochondria mediated cell death, and has been characterised in yeast [369]. mPOS is usually triggered by any dysfunction that leads to over-accumulation of precursor proteins in the cytoplasm. Usually, this accumulation would occur as a consequence of import dysfunctions, but it can also be related to other mitochondrial damage, particularly damage that alters IMM integrity such as misfolding of IMM proteins [369]. mPOS is thought to lead to cell degeneration due to the toxic cytosolic accumulation of misfolded proteins exceeding the cells’ capacity to remove these proteins [369]. However, there is a large network of genes responsible for suppressing mPOS and thus promoting cell survival by means of modulating ribosomal biogenesis, translation of specific transcripts, increasing protein chaperones and turnover, and decapping mRNA [369]. Of these proteins in yeast, Gis2 and Nog2 are particularly important in encouraging cell survival. Gis2 is involved in promoting cap-independent translation whilst Nog2 inhibits the nuclear export of the 60S RNA subunit of the ribosome, promoting cell survival and attenuating mPOS [369,370,371]. Furthermore, the mPOS pathway can trigger additional stress response pathways within the cell, including the ISR, which restores cellular homeostasis by reducing global protein synthesis, triggered by phosphorylation of eukaryotic translation initiation factor 2 alpha (eIF2α) [372].

Though there have been no specific examples of mPOS in neurodegeneration as of yet, it may have extremely important implications, especially given the mutations in genes of the anti-degenerative network seen in some neurodegenerative diseases such as ALS [373] and PD [374], which have been implicated in suppressing mPOS. The potential association of mPOS in neurodegeneration has been discussed in detail in a recent review [375].

### 5.4. mitoCPR

The mitochondrial compromised protein import response (mitoCPR) pathway was discovered in yeast and is activated when a mitochondrial protein is stalled in the Tom40 channel, inducing mitochondrial import stress and accumulation of proteins on the mitochondrial surface [376]. In yeast, the mitoCPR is activated and transcription factor Pdr3 induces the expression of *CIS1*. Cytosolic protein Cis1 binds to Tom70 and recruits the AAA+ ATPase Msp1, which removes stalled precursor proteins from mitochondrial channels and targets them for proteasomal degradation [376]. This allows mitochondria to maintain their functions under import stress conditions. This is interesting in the context of AD, especially given that APP, the precursor protein responsible for the production of toxic amyloid plaques in Alzheimer’s brains, was shown to accumulate within TOM channels, driving mitochondrial dysfunction in AD [260]. This indicates that the mitoCPR pathway may be defective under these conditions, or may not be sufficient to rescue mitochondrial dysfunction associated with APP-TOM aggregation [377].

### 5.5. mitoTAD

The mitochondrial protein translocation-associated degeneration (mitoTAD) pathway differs from those described already in that it is a quality control pathway that occurs constitutively under non-stress conditions [378]. In yeast, it is triggered by precursor proteins trapped in the Tom40 channel, sensed by Ubx2, which consistently interacts with the TOM complex under normal conditions, monitoring protein import through Tom40 [378]. If Ubx2 senses that a precursor protein is arrested within the TOM complex, a pool of Ubx2 binds to TOM and recruits the AAA+ ATPase Cdc48 for removal of arrested precursor proteins from the Tom40 channel [378]. The mitoTAD pathway was discovered in yeast, and interestingly, shows similarities to a quality control pathway in the ER, which involves Ubx2 exporting unfolded proteins from the ER [379,380]. No examples of the mitoTAD pathway have been described in models of neurodegeneration as of yet; however, as discussed above for the mitoCPR pathway, it is intriguing in the context of studies showing accumulation of proteins in the mitochondria during neurodegeneration, and further research into this link would be most interesting.

## 6. Concluding Remarks

Over recent years, remarkable progress has been made towards understanding the processes of mitochondrial protein import and respiratory complexes assembly. Recent advances in structural biology have begun to further elucidate the different structural properties of the mitochondrial translocases in high resolution, and this sheds further light on the various processes of mitochondrial protein import for specific protein classes. Whilst progress has been made, there remain areas of uncertainty regarding the organisation and dynamic action of the translocase complexes. Advances in import assay methods, such as the one recently developed [381], are also of paramount importance to dissect the mechanism of the import process and its kinetics. Hopefully, a revamped in cell assay will allow one to perform drug and phenotypic screenings, allowing for the easy identification of new players and modulators as well as small molecules that target this biological pathway.

Here, we highlight the body of evidence surrounding how closely interlinked mitochondrial protein translocation pathways are with the assembly of respiratory complexes and their function. Recent advances have begun clarifying exactly which translocation pathways are taken by nuclear-encoded respiratory complex proteins, though much more is yet to be done. Elucidating this link between import and respiratory function will be of vital importance, especially since in cases of mitochondrial disease, the importance of import pathways for respiratory complexes assembly and function is now clear. This suggests that targeting import pathways in cases of mitochondrial disease may become a credible therapeutic strategy.

Whilst mitochondrial dysfunction has long been recognised as a key factor in neurodegenerative diseases, mitochondrial protein import is now being implicated as a key factor in this dysfunction, across all levels of neurodegenerative disease models from the simple cell line setup right up to animal models and patient samples. Interestingly, the findings from these studies suggest that dysfunctional mitochondrial import is a driving force for the prominent mitochondrial irregularities observed in these diseases. This therefore represents an important target for further research to address the major outstanding questions. Namely, are the links between mitochondrial import defects and disease causative or consequential? What exact role might such defects play in disease progression?

This review has also outlined the various stress response pathways that have been shown to be activated in response to mitochondrial protein import defects. We highlight their importance in maintaining cell proteostasis and fine-tuning respiratory processes that rescue mitochondrial function. It is thought that these pathways are interlinked with one another; for example, the UPR^am^ and UPR^mt^, the two most well characterised pathways to date, are thought to be activated simultaneously, despite having different triggers [364]. Interestingly, the UPR^am^ and mPOS pathways both share a common trigger, that is, they become activated by accumulation of precursor proteins in the cytosol, yet thus far no evidence has shown their simultaneous activation. Interestingly, the mitoCPR and mitoTAD pathways also share the same trigger. It is thought that the mitoTAD pathway is active under non-stress conditions, whilst the mitoCPR pathway is activated only under stress conditions. Does this mean that mitoCPR is activated only when the mitoTAD pathway has failed? Since these stress response pathways are relatively new concepts, much more research is required. As more evidence emerges, enlightening the precise mechanisms of these pathways, they may generate therapeutically interesting targets for interventions against neurodegenerative diseases.

## Figures and Tables

**Table 1:** Structure and function of subunits of the mitochondrial translocase complexes in humans and their yeast counterparts.

Pathway	Complex		Subunit (Mammalian)	Yeast Homolog	Main Function	Topology
TOM	TOM-Holo Complex	Core complex	TOM40 and TOM40L	Tom40	Channel protein	β-barrel (19 β strands) and one N-terminal α-helical segment located inside pore
			TOM22	Tom22	Receptor protein. Located at the dimer interface between TOM40 pores.	α-helical (single TMD); C_in_-N_out_
			TOM5	Tom5	Complex assembly/stability	α-helical (single TMD); C_in_-N_out_
			TOM6	Tom6	Complex assembly/stability	α-helical (single TMD); C_in_-N_out_
			TOM7	Tom7	Complex assembly/stability	α-helical (single TMD); C_in_-N_out_
		Receptors	TOM70	Tom70	Receptor for carrier precursors	α-helical (single TMD); N-terminally inserted
			TOM20	Tom20	Receptor for presequence precursors	α-helical (single TMD); N-terminally inserted
SAM	SAM Complex		SAM50	Sam50	Core subunit responsible for β-barrel protein insertion	β-barrel (16 β-strands)
			MTX1 and MTX3	Sam37	Accessory subunit	N/A
			MTX2	Sam35	Accessory subunit	N/A
MIM	MIM Complex		Unknown	Mim1	Biogenesis of α-helical OMM proteins	--
			Unknown	Mim2	Biogenesis of α-helical OMM proteins	--
MIA	MIA Complex		CHCHD4	Mia40	Oxidoreductase	Helix-loop-helix attached to a flexible helical arm
			ALR	Erv1	Reoxidises Mia40	α-helical (a1-5) bundle
			Cytochrome C/ETC	Cytochrome C/ETC	Final electron acceptor	Class I of the c type cytochrome
			AIF	-	Anchors CHCHD4 to the IMM	One C-terminal TMD; N_in_, C_out_
TIM23/Presequence	TIM23^SORT^ Complex		TIM21	Tim21	Recognition/direction of precursor proteins to TIM23	α-helical (single TMD) with a large IMS domain; N_in_-C_out_
			ROMO1	Mgr2	Lateral release of proteins into the IMM	Two α-helical TMDs, joined by a basic loop
		TIM23^MOTOR^ Complex	TIM17A/B	Tim17	Channel forming	4 TMDs and a small IMS domain
			TIM23	Tim23	Channel forming	Multiple TMDs, and IMS exposed hydrophilic domain
			TIM50	Tim50	Receptor Protein	Single TMD, large IMS exposed C-terminal domain
	PAM Complex		TIM44	Tim44	Scaffold for complex & binding emerging precursor	Peripheral membrane protein on matrix side
			mtHSP70 (Mortalin)	SSC1 (mtHsp70)	ATPase	β-sheet and α-helical domains
			DNAJC15 and DNAJC19	Pam18 (Tim14)	Stimulates ATPase activity of mHsp70	Single α-helical TMD, with large C-terminal matrix domain and small N-terminal IMS domain
			TIM16	Pam16 (Tim16)	Inhibits Pam18 stimulatory effect on ATPase activity of mHsp70	Three α-helices forming an antiparallel hairpin
			GrpEL1/2	Mge1	Regeneration of mtHSP70	Long N-terminal α-helical region, small helical bundle region, and a C-teminal β-sheet domain
			Unknown	Pam17	Binds precursor:chaperone complex in matrix	--
TIM22/Carrier	TIM22 Complex		TIM22	Tim22	Channel	4 TMs that form a curved surface; IMS-facing N-helix
			TIM29	-	Scaffold	Matrix-facing N-helix, single TM and an IMS domain
			AGK	-	Assembly and function	N-terminally inserted with an IMS α/β motif
			TIM9	Tim9	Chaperone	Donut-shaped hexamer structure
			TIM10A	Tim10	Chaperone	
			TIM10B	Tim12	Chaperone	
			-	Tim54	Holds chaperone ring in tilted conformation	N-terminally inserted with an IMS α/β motif
			-	Tim18	Docking platform for chaperones	3 TMs and an amphipathic helix on the IMS side
			-	Sdh3	Docking platform for chaperones	3 TMs and an amphipathic helix on the IMS side
OXA Pathway	OXA Complex		OXA1L	OXA1	Insertion of mtDNA encoded proteins and N-terminal insertion of nuclear encoded proteins into the IMM	5 TM helices and a large internal C-terminal domain; N_out_-C_in_

**Table 2:** Summary of import defects associated with neurodegenerative diseases and their consequences on respiratory complexes.

Pathology	Import Defect(s)	Known Consequence(s)	Model Organism/System	Reference
Alzheimer’s Disease	APP accumulation in Tom40 and Tim23 channels, with higher levels in AD susceptible brain regions.	Inhibition of import of CIV 4 and 5b, and subsequent reduction in CIV activity, leading to increased ROS.	Human AD brains.	[260]
	Chronic, sub-lethal Aβ exposure induces a significant reduction in mitochondrial protein import.	Reduction in Δψ, altered mitochondrial morphology, and increased ROS production.	PC12 cells.	[261]
	Tau accumulation in OMM and IMS, and interactions between N-terminal Tau fragment with OPA1 and Mfn1.	N/A	HEK293T cells, HeLa cells.	[262,263]
Parkinson’s Disease	α-syn localises to and accumulates within mitochondria, mediated by a cryptic non-canonical MTS, in an ATP and Δψ dependent manner	N/A	Human dopaminergic neuronal cultures, PD brains.	[264]
	A53T version of α-syn is imported more efficiently than wildtype variant.	May account for faster development of cellular abnormalities seen in cells expressing the A53T version of α-syn compared to the wildtype.	Human dopaminergic neuronal cultures, PD brains, A53T mutant alpha-synuclein-inducible PC12 cell lines.	[265,266]
	Mitochondrial α-syn accumulates at IMM and interacts with CI.	Reduction in CI activity, increase in ROS production, inducing oxidative stress.	Human dopaminergic neuronal cultures, PD brains, rat *SN* neurons, human neuroblastoma cell line (SK-N-MC cells).	[266]
	S129 phosphorylated α-syn binds tightly to Tom20, inducing loss in Tom20-Tom22 interaction.	Impaired protein import, loss of Δψ, reduced respiratory capacity, and increased oxidative stress. Rescued by in vivo knockdown of endogenous α-syn, and by in vitro Tom20 overexpression.	SH-SY5Y cells and dopaminergic neurons from *SN* of post-mortem PD patient brains.	[267]
	Tom40 downregulation, corresponding with α-syn accumulation in PD brains.	N/A	Midbrain of PD patients and α-syn transgenic mice.	[268]
	Excessively low levels of mitochondrial import in cells from *PINK1-* and *PARK2-*linked PD patients.	N/A Import defects reversed by phosphomimetic ubiquitin in cells with residual Parkin activity.	Cells from *PINK1-* and *PARK2-*linked PD patients.	[27]
Huntington’s Disease	Disease variant Htt localises to mitochondria and directly interacts with the TIM23 complex.	Inhibited import and subsequent respiratory dysfunction, triggering cell death, rescued by TIM23 overexpression.	Isolated mitochondria from human HD brains, primary neurons expressing Htt variant, forebrain synaptosomal mitochondria in HD mice at early stages of HD.	[269]
	Dysfunctions in MIA pathway associated with mutant Htt: reduced levels and ratio of Erv1 and Mia40.	Reduced import of MIA pathway precursors, CIV assembly defects, deficient respiration, alterations in mtDNA, altered mitochondrial morphology.	Neuronal cell lines.	[270]
Amyotrophic Lateral Sclerosis	Variants of SOD1 accumulate in IMS, matrix, and OMM, and interact with OMM proteins.	Excessive ROS production, mitochondrial dysfunction, and toxic effects on the cells rescued by selective IMS targeting of wildtype SOD1.	Transgenic mouse models, spinal cord mitochondria.	[271,272,273]
	Increased levels of TOM subunits Tom20, Tom22, and Tom40. Overall reduction in import efficiency by 30%.	Changes in CI related protein expression levels.	Rat spinal cord of ALS-linked variant SOD1^G93A^.	[274]
	Novel CHCHD10 mutant, *Q108P*, discovered in a patient with rapidly progressing ALS, almost completely abolishes its import.	Reduced mitochondrial respiratory capacity, an effect that is rescued by Mia40 overexpression.	HeLa cells and primary rat embryonic neurons transduced with genomic DNA from a young ALS patient.	[275]

## Data Availability

Not applicable.

## Supplementary Materials

The following are available online at https://www.mdpi.com/article/10.3390/life11050432/s1, Table S1: Supporting Table for Main Figure 3 on the Assembly of human Respiratory Complexes.

## Abbreviations

Δψ: mitochondrial transmembrane potential; α-syn: alpha-synuclein; Aβ: amyloid-beta peptide; AD: Alzheimer’s Disease; AGK: acylglycerol kinase; AIF: apoptosis inducing factor; ALS: Amyotrophic lateral sclerosis; APP: amyloid precursor protein; CCCP: carbonyl cyanide-*m*-lorophenylhydrazone; CHCHD: coiled-coil-helix-coiled-coil-helix domain; CI-V: mitochondrial respiratory complexes one to five; cryo-EM: cryogenic electron microscopy; DAMPs: damage-associated molecular patterns; Drp1: mitochondrial fission GTPase dynamin-related protein 1; eIF2α: eukaryotic translation initiation factor 2 alpha; ER: endoplasmic reticulum; ETC: electron transport chain; Fe/S: iron/sulphur; HD: Huntington’s disease; HSR: heat shock response; Hsp: heat-shock protein; HTT: Huntingtin; IBM: inner boundary membrane; IMM: inner mitochondrial membrane; IMS: intermembrane space; iMTS: internal mitochondrial targeting sequence like signal sequence; ISR: integrated stress response; LHON: Leber hereditary optic neuropathy; MCIA: mitochondrial complex I intermediate assembly; MCSR: mitochondrial to cytosolic stress response; MDVs: mitochondrial-derived vesicles; MIA: mitochondrial IMS assembly; MICOS: mitochondrial contact site and cristae organising system; MIM: insertase of the outer mitochondrial membrane; MIP: mitochondrial intermediate peptidase; mitoCPR: mitochondrial compromised protein import response; mitoTAD: mitochondrial protein translocation-associated degeneration; MITRAC: Mitochondrial Translation Regulation Assembly intermediate of Cytochrome *c* oxidase; mPOS: mitochondrial precursor over-accumulation stress; MPP: mitochondrial processing peptidase; mtDNA: mitochondrial DNA; MTS: mitochondrial targeting sequence; NFTs: neurofibrillary tangles; NLS: nuclear localisation sequence; OMM: outer mitochondrial membrane; OXA1: mitochondrial oxidase assembly protein 1; PAM: presequence translocase-associated motor; PD: Parkinson’s disease; ROS: reactive oxygen species; SAM: sorting and assembly machinery; SC: supercomplexes; *SN*: *Substantia Nigra*; TIM22: translocase of the inner membrane 23; TIM23: translocase of the inner membrane 23; TOM: translocase of the outer membrane; TOM-CC: translocase of the outer membrane core complex; UQCC1 and UQCC2: ubiquinol-cytochrome *c* reductase complex assembly factors 1 and 2; UPR^am^: UPR activated by the mistargeting of proteins; UPR^mt^: mitochondrial unfolded protein response; UTR: untranslated region.

## References

[1] Osellame L.D., Blacker T.S., Duchen M.R. (2012). Cellular and molecular mechanisms of mitochondrial function. Best Pract. Res. Clin. Endoc. Metab..

[2] Chacinska A., Koehler C.M., Milenkovic D., Lithgow T., Pfanner N. (2009). Importing Mitochondrial Proteins: Machineries and Mechanisms. Cell.

[3] MacKenzie J.A., Payne R.M. (2007). Mitochondrial protein import and human health and disease. Biochim. Biophys. Acta Mol. Basis Dis..

[4] Briston T., Hicks A.R. (2018). Mitochondrial dysfunction and neurodegenerative proteinopathies: Mechanisms and prospects for therapeutic intervention. Biochem. Soc. Trans..

[5] Jackson T.D., Palmer C.S., Stojanovski D. (2018). Mitochondrial diseases caused by dysfunctional mitochondrial protein import. Biochem. Soc. Trans..

[6] Rath S., Sharma R., Gupta R., Ast T., Chan C., Durham T.J., Goodman R.P., Grabarek Z., Haas M.E., Hung W.H.W. (2021). MitoCarta3.0: An updated mitochondrial proteome now with sub-organelle localization and pathway annotations. Nucleic Acids Res..

[7] Tucker K., Park E. (2019). Cryo-EM structure of the mitochondrial protein-import channel TOM complex at near-atomic resolution. Nat. Struct. Mol. Biol..

[8] Sollner T., Griffiths G., Pfaller R., Pfanner N., Neupert W. (1989). Mom19, an import receptor for mitochondrial precursor proteins. Cell.

[9] Moczko M., Dietmeier K., Sollner T., Segui B., Steger H.F., Neupert W., Pfanner N. (1992). Identification of the mitochondrial receptor complex in saccharomyces-cerevisiae. FEBS Lett..

[10] Hines V., Brandt A., Griffiths G., Horstmann H., Brutsch H., Schatz G. (1990). Protein import into yeast mitochondria is accelerated by the outer-membrane protein mas70. Embo J..

[11] Sollner T., Pfaller R., Griffiths G., Pfanner N., Neupert W. (1990). A mitochondrial import receptor for the adp/atp carrier. Cell.

[12] Chacinska A., Lind M., Frazier A.E., Dudek J., Meisinger C., Geissler A., Sickmann A., Meyer H.E., Truscott K.N., Guiard B. (2005). Mitochondrial presequence translocase: Switching between TOM tethering and motor recruitment involves Tim21 and Tim17. Cell.

[13] Van Wilpe S., Ryan M.T., Hill K., Maarse A.C., Meisinger C., Brix J., Dekker P.J., Moczko M., Wagner R., Meijer M. (1999). Tom22 is a multifunctional organizer of the mitochondrial preprotein translocase. Nature.

[14] Lithgow T., Junne T., Suda K., Gratzer S., Schatz G. (1994). The mitochondrial outer membrane protein Mas22p is essential for protein import and viability of yeast. Proc. Natl. Acad. Sci. USA.

[15] Sakaue H., Shiota T., Ishizaka N., Kawano S., Tamura Y., Tan K.S., Imai K., Motono C., Hirokawa T., Taki K. (2019). Porin Associates with Tom22 to Regulate the Mitochondrial Protein Gate Assembly. Mol. Cell.

[16] Grevel A., Becker T. (2020). Porins as helpers in mitochondrial protein translocation. Biol. Chem..

[17] Harbauer A.B., Opalinska M., Gerbeth C., Herman J.S., Rao S., Schonfisch B., Guiard B., Schmidt O., Pfanner N., Meisinger C. (2014). Cell cycle-dependent regulation of mitochondrial preprotein translocase. Science.

[18] Kunkele K.P., Heins S., Dembowski M., Nargang F.E., Benz R., Thieffry M., Walz J., Lill R., Nussberger S., Neupert W. (1998). The preprotein translocation channel of the outer membrane of mitochondria. Cell.

[19] Shiota T., Imai K., Qiu J., Hewitt V.L., Tan K., Shen H.H., Sakiyama N., Fukasawa Y., Hayat S., Kamiya M. (2015). Molecular architecture of the active mitochondrial protein gate. Science.

[20] Model K., Prinz T., Ruiz T., Radermacher M., Krimmer T., Kuhlbrandt W., Pfanner N., Meisinger C. (2002). Protein translocase of the outer mitochondrial membrane: Role of import receptors in the structural organization of the TOM complex. J. Mol. Biol..

[21] Model K., Meisinger C., Kuhlbrandt W. (2008). Cryo-Electron Microscopy Structure of a Yeast Mitochondrial Preprotein Translocase. J. Mol. Biol..

[22] Bausewein T., Mills D.J., Langer J.D., Nitschke B., Nussberger S., Kuhlbrandt W. (2017). Cryo-EM Structure of the TOM Core Complex from Neurospora crassa. Cell.

[23] Moczko M., Bomer U., Kubrich M., Zufall N., Honlinger A., Pfanner N. (1997). The intermembrane space domain of mitochondrial Tom22 functions as a trans binding site for properties with N-terminal targeting sequences. Mol. Cell. Biol..

[24] Harner M., Neupert W., Deponte M. (2011). Lateral release of proteins from the TOM complex into the outer membrane of mitochondria. Embo J..

[25] Sekine S., Youle R.J. (2018). PINK1 import regulation; a fine system to convey mitochondrial stress to the cytosol. BMC Biol..

[26] Hasson S.A., Kane L.A., Yamano K., Huang C.H., Sliter D.A., Buehler E., Wang C.X., Heman-Ackah S.M., Hessa T., Guha R. (2013). High-content genome-wide RNAi screens identify regulators of parkin upstream of mitophagy. Nature.

[27] Jacoupy M., Hamon-Keromen E., Ordureau A., Erpapazoglou Z., Coge F., Corvol J.C., Nosjean O., la Cour C.M., Millan M.J., Boutin J.A. (2019). The PINK1 kinase-driven ubiquitin ligase Parkin promotes mitochondrial protein import through the presequence pathway in living cells. Sci. Rep..

[28] Phu L., Rose C.M., Tea J.S., Wall C.E., Verschueren E., Cheung T.K., Kirkpatrick D.S., Bingol B. (2020). Dynamic Regulation of Mitochondrial Import by the Ubiquitin System. Mol. Cell.

[29] Ordureau A., Paulo J.A., Zhang J.C., An H., Swatek K.N., Cannon J.R., Wan Q.Q., Komander D., Harper J.W. (2020). Global Landscape and Dynamics of Parkin and USP30-Dependent Ubiquitylomes in iNeurons during Mitophagic Signaling. Mol. Cell.

[30] Harbauer A.B., Zahedi R.P., Sickmann A., Pfanner N., Meisinger C. (2014). The Protein Import Machinery of Mitochondria-A Regulatory Hub in Metabolism, Stress, and Disease. Cell Metab..

[31] Paschen S.A., Waizenegger T., Stan T., Preuss M., Cyrklaff M., Hell K., Rapaport D., Neupert W. (2003). Evolutionary conservation of biogenesis of beta-barrel membrane proteins. Nature.

[32] Kutik S., Stojanovski D., Becker L., Becker T., Meinecke M., Kruger V., Prinz C., Meisinger C., Guiard B., Wagner R. (2008). Dissecting membrane insertion of mitochondrial beta-barrel proteins. Cell.

[33] Becker T., Voegtle F.N., Stojanovski D., Meisinger C. (2008). Sorting and assembly of mitochondrial outer membrane proteins. Biochim. Biophys. Acta-Bioenerg..

[34] Diederichs K.A., Ni X.D., Rollauer S.E., Botos I., Tan X.F., King M.S., Kunji E.R.S., Jiang J.S., Buchanan S.K. (2020). Structural insight into mitochondrial beta-barrel outer membrane protein biogenesis. Nat. Commun..

[35] Wenz L.S., Ellenrieder L., Qiu J., Bohnert M., Zufall N., van der Laan M., Pfanner N., Wiedemann N., Becker T. (2015). Sam37 is crucial for formation of the mitochondrial TOM-SAM supercomplex, thereby promoting beta-barrel biogenesis. J. Cell Biol..

[36] Meisinger C., Rissler M., Chacinska A., Szklarz L.K.S., Milenkovic D., Kozjak V., Schonfisch B., Lohaus C., Meyer H.E., Yaffe M.P. (2004). The mitochondrial morphology protein Mdm10 functions in assembly of the preprotein translocase of the outer membrane. Dev. Cell.

[37] Doan K.N., Grevel A., Martensson C.U., Ellenrieder L., Thornton N., Wenz L.S., Opalinski L., Guiard B., Pfanner N., Becker T. (2020). The Mitochondrial Import Complex MIM Functions as Main Translocase for alpha-Helical Outer Membrane Proteins. Cell Rep..

[38] Becker T., Wenz L.S., Kruger V., Lehmann W., Muller J.M., Goroncy L., Zufall N., Lithgow T., Guiard B., Chacinska A. (2011). The mitochondrial import protein Mim1 promotes biogenesis of multispanning outer membrane proteins. J. Cell Biol..

[39] Krumpe K., Frumkin I., Herzig Y., Rimon N., Ozbalci C., Brugger B., Rapaport D., Schuldiner M. (2012). Ergosterol content specifies targeting of tail-anchored proteins to mitochondrial outer membranes. Mol. Biol. Cell.

[40] Kruger V., Becker T., Becker L., Montilla-Martinez M., Ellenrieder L., Vogtle F.N., Meyer H.E., Ryan M.T., Wiedemann N., Warscheid B. (2017). Identification of new channels by systematic analysis of the mitochondrial outer membrane. J. Cell Biol..

[41] Dimmer K.S., Papic D., Schumann B., Sperl D., Krumpe K., Walther D.M., Rapaport D. (2012). A crucial role for Mim2 in the biogenesis of mitochondrial outer membrane proteins. J. Cell Sci..

[42] Wenz L.S., Opalinski L., Schuler M.H., Ellenrieder L., Ieva R., Bottinger L., Qiu J., van der Laan M., Wiedemann N., Guiard B. (2014). The presequence pathway is involved in protein sorting to the mitochondrial outer membrane. Embo Rep..

[43] Sinzel M., Tan T., Wendling P., Kalbacher H., Ozbalci C., Chelius X., Westermann B., Brugger B., Rapaport D., Dimmer K.S. (2016). Mcp3 is a novel mitochondrial outer membrane protein that follows a unique IMP-dependent biogenesis pathway. Embo Rep..

[44] Mokranjac D., Neupert W. (2008). Energetics of protein translocation into mitochondria. Biochim. Biophys. Acta Bioenerg..

[45] Komiya T., Rospert S., Schatz G., Mihara K. (1997). Binding of mitochondrial precursor proteins to the cytoplasmic domains of the import receptors Tom70 and Tom20 is determined by cytoplasmic chaperones. Embo J..

[46] Yamamoto H., Esaki M., Kanamori T., Tamura Y., Nishikawa S., Endo T. (2002). Tim50 is a subunit of the TIM23 complex that links protein translocation across the outer and inner mitochondrial membranes. Cell.

[47] Komiya T., Rospert S., Koehler C., Looser R., Schatz G., Mihara K. (1998). Interaction of mitochondrial targeting signals with acidic receptor domains along the protein import pathway: Evidence for the ‘acid chain’ hypothesis. Embo J..

[48] Dudek J., Rehling P., van der Laan M. (2013). Mitochondrial protein import: Common principles and physiological networks. Biochim. Biophys. Acta-Mol. Cell Res..

[49] Garcia M., Delaveau T., Goussard S., Jacq C. (2010). Mitochondrial presequence and open reading frame mediate asymmetric localization of messenger RNA. Embo Rep..

[50] Margeot A., Blugeon C., Sylvestre J., Vialette S., Jacq C., Corral-Debrinski M. (2002). In Saccharomyces cerevisiae, ATP2 mRNA sorting to the vicinity of mitochondria is essential for respiratory function. Embo J..

[51] Corral-Debrinski M., Blugeon C., Jacq C. (2000). In yeast, the 3’ untranslated region or the presequence of ATM1 is required for the exclusive localization of its mRNA to the vicinity of mitochondria. Mol Cell Biol.

[52] George R., Walsh P., Beddoe T., Lithgow T. (2002). The nascent polypeptide-associated complex (NAC) promotes interaction of ribosomes with the mitochondrial surface in vivo. FEBS Lett..

[53] MacKenzie J.A., Payne R.M. (2004). Ribosomes specifically bind to mammalian mitochondria via protease-sensitive proteins on the outer membrane. J. Biol. Chem..

[54] Edwards R., Eaglesfield R., Tokatlidis K. (2021). The mitochondrial intermembrane space: The most constricted mitochondrial sub-compartment with the largest variety of protein import pathways. Open Biol..

[55] Peleh V., Cordat E., Herrmann J.M. (2016). Mia40 is a trans-site receptor that drives protein import into the mitochondrial intermembrane space by hydrophobic substrate binding. eLife.

[56] Chacinska A., Pfannschmidt S., Wiedemann N., Kozjak V., Szklarz L.K.S., Schulze-Specking A., Truscott K.N., Guiard B., Meisinger C., Pfanner N. (2004). Essential role of Mia40 in import and assembly of mitochondrial intermembrane space proteins. Embo J..

[57] Banci L., Bertini I., Cefaro C., Ciofi-Baffoni S., Gallo A., Martinelli M., Sideris D.P., Katrakili N., Tokatlidis K. (2009). MIA40 is an oxidoreductase that catalyzes oxidative protein folding in mitochondria. Nat. Struct. Mol. Biol..

[58] Reinhardt C., Arena G., Nedara K., Edwards R., Brenner C., Tokatlidis K., Modjtahedi N. (2020). AIF meets the CHCHD4/Mia40-dependent mitochondrial import pathway. Biochim. Biophys. Acta Mol. Basis Dis..

[59] Bien M., Longen S., Wagener N., Chwalla I., Herrmann J.M., Riemer J. (2010). Mitochondrial Disulfide Bond Formation Is Driven by Intersubunit Electron Transfer in Erv1 and Proofread by Glutathione. Mol. Cell.

[60] Allen S., Balabanidou V., Sideris D.P., Lisowsky T., Tokatlidis K. (2005). Erv1 mediates the Mia40-dependent protein import pathway and provides a functional link to the respiratory chain by shuttling electrons to cytochrome c. J. Mol. Biol..

[61] Neupert W. (1997). Protein import into mitochondria. Annu. Rev. Biochem..

[62] Backes S., Hess S., Boos F., Woellhaf M.W., Godel S., Jung M., Muhlhaus T., Herrmann J.M. (2018). Tom70 enhances mitochondrial preprotein import efficiency by binding to internal targeting sequences. J. Cell Biol..

[63] Demishtein-Zohary K., Azem A. (2017). The TIM23 mitochondrial protein import complex: Function and dysfunction. Cell Tissue Res..

[64] Maarse A.C., Blom J., Keil P., Pfanner N., Meijer M. (1994). Identification of the essential yeast protein mim17, an integral mitochondrial inner membrane-protein involved in protein import. FEBS Lett..

[65] Dekker P.J.T., Keil P., Rassow J., Maarse A.C., Pfanner N., Meijer M. (1993). Identification of mim23, a putative component of the protein import machinery of the mitochondrial inner membrane. FEBS Lett..

[66] Geissler A., Chacinska A., Truscott K.N., Wiedemann N., Brandner K., Sickmann A., Meyer H.E., Meisinger C., Pfanner N., Rehling P. (2002). The mitochondrial presequence translocase: An essential role of Tim50 in directing preproteins to the import channel. Cell.

[67] Gebert M., Schrempp S.G., Mehnert C.S., Heisswolf A.K., Oeljeklaus S., Ieva R., Bohnert M., von der Malsburg K., Wiese S., Kleinschroth T. (2012). Mgr2 promotes coupling of the mitochondrial presequence translocase to partner complexes. J. Cell Biol..

[68] Maarse A.C., Blom J., Grivell L.A., Meijer M. (1992). Mpi1, an essential gene encoding a mitochondrial-membrane protein, is possibly involved in protein import into yeast mitochondria. Embo J..

[69] Kang P.J., Ostermann J., Shilling J., Neupert W., Craig E.A., Pfanner N. (1990). Requirement for hsp70 in the mitochondrial matrix for translocation and folding of precursor proteins. Nature.

[70] Frazier A.E., Dudek J., Guiard B., Voos W., Li Y.F., Lind M., Meisinger C., Geissler A., Sickmann A., Meyer H.E. (2004). Pam16 has an essential role in the mitochondrial protein import motor. Nat. Struct. Mol. Biol..

[71] Truscott K.N., Voos W., Frazier A.E., Lind M., Li Y.F., Geissler A., Dudek J., Muller H., Sickmann A., Meyer H.E. (2003). A J-protein is an essential subunit of the presequence translocase-associated protein import motor of mitochondria. J. Cell Biol..

[72] Van der Laan M., Chacinska A., Lind M., Perschil I., Sickmann A., Meyer H.E., Guiard B., Meisinger C., Pfanner N., Rehling P. (2005). Pam17 is required for architecture and translocation activity of the mitochondrial protein import motor. Mol. Cell. Biol..

[73] Laloraya S., Gambill B.D., Craig E.A. (1994). A role for a eukaryotic grpe-related protein, mge1p, in protein translocation. Proc. Natl. Acad. Sci. USA.

[74] Tamura Y., Harada Y., Shiota T., Yamano K., Watanabe K., Yokota M., Yamamoto H., Sesaki H., Endo T. (2009). Tim23-Tim50 pair coordinates functions of translocators and motor proteins in mitochondrial protein import. J. Cell Biol..

[75] Schwartz M.P., Matouschek A. (1999). The dimensions of the protein import channels in the outer and inner mitochondrial membranes. Proc. Natl. Acad. Sci. USA.

[76] Truscott K.N., Kovermann P., Geissler A., Merlin A., Meijer M., Driessen A.J.M., Rassow J., Pfanner N., Wagner R. (2001). A presequence- and voltage-sensitive channel of the mitochondrial preprotein translocase formed by Tim23. Nat. Struct. Biol..

[77] Martinez-Caballero S., Grigoriev S.M., Herrmann J.M., Campo M.L., Kinnally K.W. (2007). Tim17p regulates the twin pore structure and voltage gating of the mitochondrial protein import complex TIM23. J. Biol. Chem..

[78] Chacinska A., Rehling P., Guiard B., Frazier A.E., Schulze-Specking A., Pfanner N., Voos W., Meisinger C. (2003). Mitochondrial translocation contact sites: Separation of dynamic and stabilizing elements in formation of a TOM-TIM-preprotein supercomplex. Embo J..

[79] Waegemann K., Popov-Celeketic D., Neupert W., Azem A., Mokranjac D. (2015). Cooperation of TOM and TIM23 complexes during translocation of proteins into mitochondria. J Mol. Biol..

[80] Shiota T., Mabuchi H., Tanaka-Yamano S., Yamano K., Endo T. (2011). In vivo protein-interaction mapping of a mitochondrial translocator protein Tom22 at work. Proc. Natl. Acad. Sci. USA.

[81] Niemi N.M., Wilson G.M., Overmyer K.A., Vogtle F.N., Myketin L., Lohman D.C., Schueler K.L., Attie A.D., Meisinger C., Coon J.J. (2019). Pptc7 is an essential phosphatase for promoting mammalian mitochondrial metabolism and biogenesis. Nat. Commun..

[82] Marom M., Dayan D., Demishtein-Zohary K., Mokranjac D., Neupert W., Azem A. (2011). Direct Interaction of Mitochondrial Targeting Presequences with Purified Components of the TIM23 Protein Complex. J. Biol. Chem..

[83] Ting S.Y., Yan N.L., Schilke B.A., Craig E.A. (2017). Dual interaction of scaffold protein Tim44 of mitochondrial import motor with channel-forming translocase subunit Tim23. eLife.

[84] Neupert W., Brunner M. (2002). The protein import motor of mitochondria. Nat. Rev. Mol. Cell Biol..

[85] Gruhler A., Arnold I., Seytter T., Guiard B., Schwarz E., Neupert W., Stuart R.A. (1997). N-terminal hydrophobic sorting signals of preproteins confer mitochondrial hsp70 independence for import into mitochondria. J. Biol. Chem..

[86] Van der Laan M., Wiedemann N., Mick D.U., Guiard B., Rehling P., Pfanner N. (2006). A role for Tim21 in membrane-potential-dependent preprotein sorting in mitochondria. Curr. Biol..

[87] Albrecht R., Rehling P., Chacinska A., Brix J., Cadamuro S.A., Volkmer R., Guiard B., Pfanner N., Zeth K. (2006). The Tim21 binding domain connects the preprotein translocases of both mitochondrial membranes. Embo Rep..

[88] Mokranjac D., Popov-Celeketic D., Hell K., Neupert W. (2005). Role of Tim21 in mitochondrial translocation contact sites. J. Biol. Chem..

[89] Ieva R., Schrempp S.G., Opalinski L., Wollweber F., Hoss P., Heisswolf A.K., Gebert M., Zhang Y., Guiard B., Rospert S. (2014). Mgr2 Functions as Lateral Gatekeeper for Preprotein Sorting in the Mitochondrial Inner Membrane. Mol. Cell.

[90] Endres M., Neupert W., Brunner M. (1999). Transport of the ADP ATP carrier of mitochondria from the TOM complex to the TIM22.54 complex. Embo J..

[91] Curran S.P., Leuenberger D., Oppliger W., Koehler C.M. (2002). The Tim9p-Tim10p complex binds to the transmembrane domains of the ADP/ATP carrier. Embo J..

[92] Rehling P., Model K., Brandner K., Kovermann P., Sickmann A., Meyer H.E., Kuhlbrandt W., Wagner R., Truscott K.N., Pfanner N. (2003). Protein insertion into the mitochondrial inner membrane by a twin-pore translocase. Science.

[93] Brix J., Rudiger S., Bukau B., Schneider-Mergener J., Pfanner N. (1999). Distribution of binding sequences for the mitochondrial import receptors Tom20, Tom22, and Tom70 in a presequence-carrying preprotein and a non-cleavable preprotein. J. Biol. Chem..

[94] Curran S.P., Leuenberger D., Schmidt E., Koehler C.M. (2002). The role of the Tim8p-Tim13p complex in a conserved import pathway for mitochondrial polytopic inner membrane proteins. J. Cell Biol..

[95] Qi L.B., Wang Q., Guan Z.Y., Wu Y., Shen C.C., Hong S.X., Cao J.B., Zhang X., Yan C.Y., Yin P. (2021). Cryo-EM structure of the human mitochondrial translocase TIM22 complex. Cell Res..

[96] Zhang Y., Ou X., Wang X., Sun D., Zhou X., Wu X., Li Q., Li L. (2021). Structure of the mitochondrial TIM22 complex from yeast. Cell Res.

[97] Callegari S., Richter F., Chojnacka K., Jans D.C., Lorenzi I., Pacheu-Grau D., Jakobs S., Lenz C., Urlaub H., Dudek J. (2016). TIM29 is a subunit of the human carrier translocase required for protein transport. Febs Lett..

[98] Kang Y.L., Baker M.J., Liem M., Louber J., McKenzie M., Atukorala I., Ang C.S., Keerthikumar S., Mathivanan S., Stojanovski D. (2016). Tim29 is a novel subunit of the human TIM22 translocase and is involved in complex assembly and stability. Elife.

[99] Vukotic M., Nolte H., Konig T., Saita S., Ananjew M., Kruger M., Tatsuta T., Langer T. (2017). Acylglycerol Kinase Mutated in Sengers Syndrome Is a Subunit of the TIM22 Protein Translocase in Mitochondria. Mol. Cell.

[100] Kang Y.L., Stroud D.A., Baker M.J., De Souza D.P., Frazier A.E., Liem M., Tull D., Mathivanan S., McConville M.J., Thorburn D.R. (2017). Sengers Syndrome-Associated Mitochondrial Acylglycerol Kinase Is a Subunit of the Human TIM22 Protein Import Complex. Mol. Cell.

[101] Wiedemann N., Pfanner N., Kornberg R.D. (2017). Mitochondrial Machineries for Protein Import and Assembly. Annual Review of Biochemistry.

[102] Rehling P., Brandner K., Pfanner N. (2004). Mitochondrial import and the twin-pore translocase. Nat. Rev. Mol. Cell Biol..

[103] Wu Y.K., Sha B.D. (2006). Crystal structure of yeast mitochondrial outer membrane translocon member Tom70p. Nat. Struct. Mol. Biol..

[104] Young J.C., Hoogenraad N.J., Hartl F.U. (2003). Molecular chaperones Hsp90 and Hsp70 deliver preproteins to the mitochondrial import receptor Tom70. Cell.

[105] Bhangoo M.K., Tzankov S., Fan A.C.Y., Dejgaard K., Thomas D.Y., Young J.C. (2007). Multiple 40-kDa heat-shock protein chaperones function in Tom70-dependent mitochondrial import. Mol. Biol. Cell.

[106] Backes S., Bykov Y.S., Räschle M., Zhou J., Lenhard S., Krämer L., Mühlhaus T., Bibi C., Jann C., Smith J.D. (2020). The mitochondrial surface receptor Tom70 protects the cytosol against mitoprotein-induced stress. bioRxiv.

[107] Wiedemann N., Pfanner N., Ryan M.T. (2001). The three modules of ADP/ATP carrier cooperate in receptor recruitment and translocation into mitochondria. Embo J..

[108] Ellenrieder L., Dieterle M.P., Doan K.N., Martensson C.U., Floerchinger A., Campo M.L., Pfanner N., Becker T. (2019). Dual Role of Mitochondrial Porin in Metabolite Transport across the Outer Membrane and Protein Transfer to the Inner Membrane. Mol. Cell.

[109] Callegari S., Muller T., Schulz C., Lenz C., Jans D.C., Wissel M., Opazo F., Rizzoli S.O., Jakobs S., Urlaub H. (2019). A MICOS-TIM22 Association Promotes Carrier Import into Human Mitochondria. J. Mol. Biol..

[110] Rampelt H., Sucec I., Bersch B., Horten P., Perschil I., Martinou J.C., van der Laan M., Wiedemann N., Schanda P., Pfanner N. (2020). The mitochondrial carrier pathway transports non-canonical substrates with an odd number of transmembrane segments. BMC Biol..

[111] Jackson T.D., Hock D.H., Fujihara K.M., Palmer C.S., Frazier A.E., Low Y.C., Kang Y., Ang C.-S., Clemons N.J., Thorburn D.R. (2021). The TIM22 complex mediates the import of Sideroflexins and is required for efficient mitochondrial one-carbon metabolism. Mol. Biol. Cell.

[112] Luirink J., Samuelsson T., de Gier J.W. (2001). YidC/Oxa1p/Alb3: Evolutionarily conserved mediators of membrane protein assembly. FEBS Lett..

[113] Bonnefoy N., Chalvet F., Hamel P., Slonimski P.P., Dujardin G. (1994). OXA1, a saccharomyces-cerecconserved from prokaryotes to eukaryotes controls cytochrome-oxidase biogenesis. J. Mol. Biol..

[114] Herrmann J.M., Neupert W., Stuart R.A. (1997). Insertion into the mitochondrial inner membrane of a polytopic protein, the nuclear-encoded Oxa1p. Embo J..

[115] Nargang F.E., Preuss M., Neupert W., Herrmann J.M. (2002). The oxal protein forms a homooligomeric complex and is an essential part of the mitochondrial export translocase in Neurospora crassa. J. Biol. Chem..

[116] Stiburek L., Fornuskova D., Wenchich L., Pejznochova M., Hansikova H., Zeman J. (2007). Knockdown of human Oxa1l impairs the biogenesis of F1Fo-ATP synthase and NADH: Ubiquinone oxidoreductase. J. Mol. Biol..

[117] Haque M.E., Elmore K.B., Tripathy A., Koc H., Koc E.C., Spremulli L.L. (2010). Properties of the C-terminal Tail of Human Mitochondrial Inner Membrane Protein Oxa1L and Its Interactions with Mammalian Mitochondrial Ribosomes. J. Biol. Chem..

[118] Itoh Y., Andrell J., Choi A., Richter U., Maiti P., Best R.B., Barrientos A., Battersby B.J., Amunts A. (2021). Mechanism of membrane-tethered mitochondrial protein synthesis. Science.

[119] Stuart R.A. (2002). Insertion of proteins into the inner membrane of mitochondria: The role of the Oxa1 complex. Biochim. Biophys. Acta Mol. Cell Res..

[120] Bohnert M., Rehling P., Guiard B., Herrmann J.M., Pfanner N., van der Laan M. (2010). Cooperation of Stop-Transfer and Conservative Sorting Mechanisms in Mitochondrial Protein Transport. Curr. Biol..

[121] Anghel S.A., McGilvray P.T., Hegde R.S., Keenan R.J. (2017). Identification of Oxa1 Homologs Operating in the Eukaryotic Endoplasmic Reticulum. Cell Rep..

[122] Pleiner T., Tomaleri G.P., Januszyk K., Inglis A.J., Hazu M., Voorhees R.M. (2020). Structural basis for membrane insertion by the human ER membrane protein complex. Science.

[123] Bai L., You Q.L., Feng X., Kovach A., Li H.L. (2020). Structure of the ER membrane complex, a transmembrane-domain insertase. Nature.

[124] Chitwood P.J., Juszkiewicz S., Guna A., Shao S.C., Hegde R.S. (2018). EMC Is Required to Initiate Accurate Membrane Protein Topogenesis. Cell.

[125] Rojo E.E., Stuart R.A., Neupert W. (1995). Conservative sorting of f-0-atpase subunit-9—export from matrix requires delta-ph across inner membrane and matrix atp. Embo J..

[126] Hell K., Neupert W., Stuart R.A. (2001). Oxa1p acts as a general membrane insertion machinery for proteins encoded by mitochondrial DNA. Embo J..

[127] Herrmann J.M., Bonnefoy N. (2004). Protein export across the inner membrane of mitochondria—The nature of translocated domains determines the dependence on the Oxa1 translocase. J. Biol. Chem..

[128] Schagger H., Pfeiffer K. (2000). Supercomplexes in the respiratory chains of yeast and mammalian mitochondria. Embo J..

[129] Acin-Perez R., Bayona-Bafaluy M.P., Fernandez-Silva P., Moreno-Loshuertos R., Perez-Martos A., Bruno C., Moraes C.T., Enriquez J.A. (2004). Respiratory complex III is required to maintain complex I in mammalian mitochondria. Mol. Cell.

[130] Diaz F., Fukui H., Garcia S., Moraes C.T. (2006). Cytochrome c oxidase is required for the assembly/stability of respiratory complex I in mouse fibroblasts. Mol. Cell Biol..

[131] Protasoni M., Pérez-Pérez R., Lobo-Jarne T., Harbour M.E., Ding S., Peñas A., Diaz F., Moraes C.T., Fearnley I.M., Zeviani M. (2020). Respiratory supercomplexes act as a platform for complex III-mediated maturation of human mitochondrial complexes I and IV. Embo J..

[132] Blakely E.L., Mitchell A.L., Fisher N., Meunier B., Nijtmans L.G., Schaefer A.M., Jackson M.J., Turnbull D.M., Taylor R.W. (2005). A mitochondrial cytochrome b mutation causing severe respiratory chain enzyme deficiency in humans and yeast. FEBS J..

[133] Andreu A.L., Hanna M.G., Reichmann H., Bruno C., Penn A.S., Tanji K., Pallotti F., Iwata S., Bonilla E., Lach B. (1999). Exercise intolerance due to mutations in the cytochrome b gene of mitochondrial DNA. N. Engl. J. Med..

[134] Lamantea E., Carrara F., Mariotti C., Morandi L., Tiranti V., Zeviani M. (2002). A novel nonsense mutation (Q352X) in the mitochondrial cytochrome b gene associated with a combined deficiency of complexes I and III. Neuromuscul. Disord. NMD.

[135] Moreno-Lastres D., Fontanesi F., Garcia-Consuegra I., Martin M.A., Arenas J., Barrientos A., Ugalde C. (2012). Mitochondrial complex I plays an essential role in human respirasome assembly. Cell Metab..

[136] Wiedemann N., van der Laan M., Hutu D.P., Rehling P., Pfanner N. (2007). Sorting switch of mitochondrial presequence translocase involves coupling of motor module to respiratory chain. J. Cell Biol..

[137] Guerrero-Castillo S., Baertling F., Kownatzki D., Wessels H.J., Arnold S., Brandt U., Nijtmans L. (2017). The Assembly Pathway of Mitochondrial Respiratory Chain Complex I. Cell Metab..

[138] Fontanesi F., Soto I.C., Horn D., Barrientos A. (2010). Mss51 and Ssc1 facilitate translational regulation of cytochrome c oxidase biogenesis. Mol. Cell Biol..

[139] Böttinger L., Guiard B., Oeljeklaus S., Kulawiak B., Zufall N., Wiedemann N., Warscheid B., van der Laan M., Becker T. (2013). A complex of Cox4 and mitochondrial Hsp70 plays an important role in the assembly of the cytochrome c oxidase. Mol. Biol. Cell.

[140] Rieger B., Junge W., Busch K.B. (2014). Lateral pH gradient between OXPHOS complex IV and F0F1 ATP-synthase in folded mitochondrial membranes. Nat. Commun..

[141] Couvillion M.T., Soto I.C., Shipkovenska G., Churchman L.S. (2016). Synchronized mitochondrial and cytosolic translation programs. Nature.

[142] Herrmann J.M., Woellhaf M.W., Bonnefoy N. (2013). Control of protein synthesis in yeast mitochondria: The concept of translational activators. Biochim. Biophys. Acta.

[143] Ott M., Amunts A., Brown A. (2016). Organization and Regulation of Mitochondrial Protein Synthesis. Annu. Rev. Biochem..

[144] Dennerlein S., Wang C., Rehling P. (2017). Plasticity of Mitochondrial Translation. Trends Cell Biol..

[145] Timón-Gómez A.N.E., Abriata L.A., Vila A.J., Hosler J., Barrientos A. (2018). Mitochondrial cytochrome c oxidase biogenesis: Recent developments. Semin Cell Dev. Biol..

[146] Gruschke S., Römpler K., Hildenbeutel M., Kehrein K., Kühl I., Bonnefoy N., Ott M. (2012). The Cbp3-Cbp6 complex coordinates cytochrome b synthesis with bc(1) complex assembly in yeast mitochondria. J. Cell Biol..

[147] Hildenbeutel M., Hegg E.L., Stephan K., Gruschke S., Meunier B., Ott M. (2014). Assembly factors monitor sequential hemylation of cytochrome b to regulate mitochondrial translation. J. Cell Biol..

[148] García-Villegas R., Camacho-Villasana Y., Shingú-Vázquez M., Cabrera-Orefice A., Uribe-Carvajal S., Fox T.D., Pérez-Martínez X. (2017). The Cox1 C-terminal domain is a central regulator of cytochrome c oxidase biogenesis in yeast mitochondria. J. Biol. Chem..

[149] Perez-Martinez X., Butler C.A., Shingu-Vazquez M., Fox T.D. (2009). Dual functions of Mss51 couple synthesis of Cox1 to assembly of cytochrome c oxidase in Saccharomyces cerevisiae mitochondria. Mol. Biol Cell.

[150] Tavares-Carreón F., Camacho-Villasana Y., Zamudio-Ochoa A., Shingú-Vázquez M., Torres-Larios A., Pérez-Martínez X. (2008). The pentatricopeptide repeats present in Pet309 are necessary for translation but not for stability of the mitochondrial COX1 mRNA in yeast. J. Biol. Chem..

[151] Zamudio-Ochoa A., Camacho-Villasana Y., García-Guerrero A.E., Pérez-Martínez X. (2014). The Pet309 pentatricopeptide repeat motifs mediate efficient binding to the mitochondrial COX1 transcript in yeast. RNA Biol..

[152] Godard F., Tetaud E., Duvezin-Caubet S., di Rago J.P. (2011). A genetic screen targeted on the FO component of mitochondrial ATP synthase in Saccharomyces cerevisiae. J. Biol. Chem..

[153] Helfenbein K.G., Ellis T.P., Dieckmann C.L., Tzagoloff A. (2003). ATP22, a nuclear gene required for expression of the F0 sector of mitochondrial ATPase in Saccharomyces cerevisiae. J. Biol. Chem..

[154] Kellems R.E., Allison V.F., Butow R.A. (1975). Cytoplasmic type 80S ribosomes associated with yeast mitochondria. IV. Attachment of ribosomes to the outer membrane of isolated mitochondria. J. Cell Biol..

[155] Ades I.Z., Butow R.A. (1980). The transport of proteins into yeast mitochondria. Kinetics and pools. J. Biol. Chem..

[156] Suissa M., Schatz G. (1982). Import of proteins into mitochondria. Translatable mRNAs for imported mitochondrial proteins are present in free as well as mitochondria-bound cytoplasmic polysomes. J. Biol. Chem..

[157] Hwang S.T., Wachter C., Schatz G. (1991). Protein import into the yeast mitochondrial matrix. A new translocation intermediate between the two mitochondrial membranes. J. Biol. Chem..

[158] Garcia M.D.X., Devaux F., Singer R.H., Jacq C. (2007). Yeast Mitochondrial Transcriptomics.

[159] Marc P., Margeot A., Devaux F., Blugeon C., Corral-Debrinski M., Jacq C. (2002). Genome-wide analysis of mRNAs targeted to yeast mitochondria. EMBO Rep..

[160] Garcia M., Darzacq X., Delaveau T., Jourdren L., Singer R.H., Jacq C. (2007). Mitochondria-associated yeast mRNAs and the biogenesis of molecular complexes. Mol. Biol. Cell.

[161] Saint-Georges Y., Garcia M., Delaveau T., Jourdren L., Le Crom S., Lemoine S., Tanty V., Devaux F., Jacq C. (2008). Yeast mitochondrial biogenesis: A role for the PUF RNA-binding protein Puf3p in mRNA localization. PLoS ONE.

[162] Gadir N., Haim-Vilmovsky L., Kraut-Cohen J., Gerst J.E. (2011). Localization of mRNAs coding for mitochondrial proteins in the yeast Saccharomyces cerevisiae. RNA.

[163] Fox T. (2012). Mitochondrial Protein Synthesis, Import, and Assembly. Genetics.

[164] Gilkerson R.W., Selker J.M., Capaldi R.A. (2003). The cristal membrane of mitochondria is the principal site of oxidative phosphorylation. FEBS Lett..

[165] Vogel F., Bornhövd C., Neupert W., Reichert A.S. (2006). Dynamic subcompartmentalization of the mitochondrial inner membrane. J. Cell Biol..

[166] Stoldt S., Wenzel D., Kehrein K., Riedel D., Ott M., Jakobs S. (2018). Spatial orchestration of mitochondrial translation and OXPHOS complex assembly. Nat. Cell Biol..

[167] Paumard P., Vaillier J., Coulary B., Schaeffer J., Soubannier V., Mueller D.M., Brethes D., di Rago J.P., Velours J. (2002). The ATP synthase is involved in generating mitochondrial cristae morphology. Embo J..

[168] Spikes T.E., Montgomery M.G., Walker J.E. (2021). Interface mobility between monomers in dimeric bovine ATP synthase participates in the ultrastructure of inner mitochondrial membranes. Proc. Natl. Acad. Sci. USA.

[169] Kim H.J., Khalimonchuk O., Smith P.M., Winge D.R. (2012). Structure, function, and assembly of heme centers in mitochondrial respiratory complexes. Biochim. Biophys. Acta Mol. Cell Res..

[170] Stram A.R., Payne R.M. (2016). Post-translational modifications in mitochondria: Protein signaling in the powerhouse. Cell. Mol. Life Sci..

[171] Cardenas-Rodriguez M., Chatzi A., Tokatlidis K. (2018). Iron-sulfur clusters: From metals through mitochondria biogenesis to disease. J. Biol. Inorg. Chem..

[172] Vinothkumar K.R., Zhu J., Hirst J. (2014). Architecture of mammalian respiratory complex I. Nature.

[173] Carroll J., Ding S., Fearnley I.M., Walker J.E. (2013). Post-translational modifications near the quinone binding site of mammalian complex I. J. Biol. Chem..

[174] Signes A., Fernandez-Vizarra E. (2018). Assembly of mammalian oxidative phosphorylation complexes I-V and supercomplexes. Essays Biochem..

[175] Formosa L.E., Muellner-Wong L., Reljic B., Sharpe A.J., Jackson T.D., Beilharz T.H., Stojanovski D., Lazarou M., Stroud D.A., Ryan M.T. (2020). Dissecting the Roles of Mitochondrial Complex I Intermediate Assembly Complex Factors in the Biogenesis of Complex I. Cell Rep..

[176] Andrews B., Carroll J., Ding S.J., Fearnley I.M., Walker J.E. (2013). Assembly factors for the membrane arm of human complex I. Proc. Natl. Acad. Sci. USA.

[177] Stroud D.A., Surgenor E.E., Formosa L.E., Reljic B., Frazier A.E., Dibley M.G., Osellame L.D., Stait T., Beilharz T.H., Thorburn D.R. (2016). Accessory subunits are integral for assembly and function of human mitochondrial complex I. Nature.

[178] Claros M.G., Vincens P. (1996). Computational method to predict mitochondrially imported proteins and their targeting sequences. Eur. J. Biochem..

[179] Szklarczyk R., Wanschers B.F.J., Nabuurs S.B., Nouws J., Nijtmans L.G., Huynen M.A. (2011). NDUFB7 and NDUFA8 are located at the intermembrane surface of complex I. FEBS Lett..

[180] Friederich M.W., Erdogan A.J., Coughlin C.R., Elos M.T., Jiang H., O’Rourke C.P., Lovell M.A., Wartchow E., Gowan K., Chatfield K.C. (2017). Mutations in the accessory subunit NDUFB10 result in isolated complex I deficiency and illustrate the critical role of intermembrane space import for complex I holoenzyme assembly. Hum. Mol. Genet..

[181] Sánchez-Caballero L., Ruzzenente B., Bianchi L., Assouline Z., Barcia G., Metodiev M.D., Rio M., Funalot B., van den Brand M.A., Guerrero-Castillo S. (2016). Mutations in Complex I Assembly Factor TMEM126B Result in Muscle Weakness and Isolated Complex I Deficiency. Am. J. Hum. Genet..

[182] Zarsky V., Dolezal P. (2016). Evolution of the Tim17 protein family. Biol. Direct.

[183] Wang Y., Carrie C., Giraud E., Elhafez D., Narsai R., Duncan O., Whelan J., Murcha M.W. (2012). Dual Location of the Mitochondrial Preprotein Transporters B14.7 and Tim23-2 in Complex I and the TIM17:23 Complex in Arabidopsis Links Mitochondrial Activity and Biogenesis. Plant Cell.

[184] Sheftel A.D., Stehling O., Pierik A.J., Netz D.J., Kerscher S., Elsasser H.P., Wittig I., Balk J., Brandt U., Lill R. (2009). Human ind1, an iron-sulfur cluster assembly factor for respiratory complex I. Mol. Cell Biol..

[185] Bych K., Kerscher S., Netz D.J., Pierik A.J., Zwicker K., Huynen M.A., Lill R., Brandt U., Balk J. (2008). The iron-sulphur protein Ind1 is required for effective complex I assembly. Embo J..

[186] Sun F., Huo X., Zhai Y., Wang A., Xu J., Su D., Bartlam M., Rao Z. (2005). Crystal structure of mitochondrial respiratory membrane protein complex II. Cell.

[187] Van Vranken J., Na U., Winge D.R., Rutter J. (2015). Protein-mediated assembly of succinate dehydrogenase and its cofactors. Crit. Rev. Biochem. Mol. Biol..

[188] Hao H.-X., Khalimonchuk O., Schraders M., Dephoure N., Bayley J.-P., Kunst H., Devilee P., Cremers C.W.R.J., Schiffman J.D., Bentz B.G. (2009). SDH5, a gene required for flavination of succinate dehydrogenase, is mutated in paraganglioma. Science.

[189] Cecchini G. (2003). Function and structure of complex II of the respiratory chain. Annu Rev. Biochem..

[190] Ghezzi D., Goffrini P., Uziel G., Horvath R., Klopstock T., Lochmüller H., D’Adamo P., Gasparini P., Strom T.M., Prokisch H. (2009). SDHAF1, encoding a LYR complex-II specific assembly factor, is mutated in SDH-defective infantile leukoencephalopathy. Nat. Genet..

[191] Maio N., Ghezzi D., Verrigni D., Rizza T., Bertini E., Martinelli D., Zeviani M., Singh A., Carrozzo R., Rouault T.A. (2016). Disease-Causing SDHAF1 Mutations Impair Transfer of Fe-S Clusters to SDHB. Cell Metab..

[192] Na U., Yu W., Cox J., Bricker D.K., Brockmann K., Rutter J., Thummel C.S., Winge D.R. (2014). The LYR factors SDHAF1 and SDHAF3 mediate maturation of the iron-sulfur subunit of succinate dehydrogenase. Cell Metab..

[193] Gebert N., Gebert M., Oeljeklaus S., von der Malsburg K., Stroud D.A., Kulawiak B., Wirth C., Zahedi R.P., Dolezal P., Wiese S. (2011). Dual Function of Sdh3 in the Respiratory Chain and TIM22 Protein Translocase of the Mitochondrial Inner Membrane. Mol. Cell.

[194] Schagger H., Link T.A., Engel W.D., von Jagow G. (1986). Isolation of the eleven protein subunits of the bc1 complex from beef heart. Methods Enzymol..

[195] Iwata S., Lee J.W., Okada K., Lee J.K., Iwata M., Rasmussen B., Link T.A., Ramaswamy S., Jap B.K. (1998). Complete structure of the 11-subunit bovine mitochondrial cytochrome bc1 complex. Science.

[196] Thompson K., Mai N., Oláhová M., Scialó F., Formosa L.E., Stroud D.A., Garrett M., Lax N.Z., Robertson F.M., Jou C. (2018). OXA1L mutations cause mitochondrial encephalopathy and a combined oxidative phosphorylation defect. EMBO Mol. Med..

[197] Cogliati S., Lorenzi I., Rigoni G., Caicci F., Soriano M.E. (2018). Regulation of Mitochondria! Electron Transport Chain Assembly. J. Mol. Biol..

[198] Ndi M., Marin-Buera L., Salvatori R., Singh A.P., Ott M. (2018). Biogenesis of the bc1 Complex of the Mitochondrial Respiratory Chain. J. Mol. Biol..

[199] Tucker E.J., Wanschers B.F., Szklarczyk R., Mountford H.S., Wijeyeratne X.W., van den Brand M.A., Leenders A.M., Rodenburg R.J., Reljic B., Compton A.G. (2013). Mutations in the UQCC1-interacting protein, UQCC2, cause human complex III deficiency associated with perturbed cytochrome b protein expression. PLoS Genet..

[200] Wanschers B.F.J., Szklarczyk R., van den Brand M.A.M., Jonckheere A., Suijskens J., Smeets R., Rodenburg R.J., Stephan K., Helland I.B., Elkamil A. (2014). A mutation in the human CBP4 ortholog UQCC3 impairs complex III assembly, activity and cytochrome b stability. Hum. Mol. Genet..

[201] Zara V., Palmisano I., Conte L., Trumpower B.L. (2004). Further insights into the assembly of the yeast cytochrome bc1 complex based on analysis of single and double deletion mutants lacking supernumerary subunits and cytochrome b. Eur. J. Biochem..

[202] Zara V., Conte L., Trumpower B.L. (2007). Identification and characterization of cytochrome bc(1) subcomplexes in mitochondria from yeast with single and double deletions of genes encoding cytochrome bc(1) subunits. FEBS J..

[203] Zara V., Conte L., Trumpower B.L. (2009). Biogenesis of the yeast cytochrome bc1 complex. Biochim. Biophys. Acta.

[204] Zara V., Conte L., Trumpower B.L. (2009). Evidence that the assembly of the yeast cytochrome bc1 complex involves the formation of a large core structure in the inner mitochondrial membrane. FEBS J..

[205] Stephan K., Ott M. (2020). Timing of dimerization of the bc(1) complex during mitochondrial respiratory chain assembly. Biochim. Et Biophys. Acta. Bioenerg..

[206] Xia D., Yu C.A., Kim H., Xia J.Z., Kachurin A.M., Zhang L., Yu L., Deisenhofer J. (1997). Crystal structure of the cytochrome bc1 complex from bovine heart mitochondria. Science.

[207] Sadler I., Suda K., Schatz G., Kaudewitz F., Haid A. (1984). Sequencing of the nuclear gene for the yeast cytochrome c1 precursor reveals an unusually complex amino-terminal presequence. Embo J..

[208] Arnold I., Folsch H., Neupert W., Stuart R.A. (1998). Two distinct and independent mitochondrial targeting signals function in the sorting of an inner membrane protein, cytochrome c1. J. Biol. Chem..

[209] Wachter C., Schatz G., Glick B.S. (1992). Role of atp in the intramitochondrial sorting of cytochrome-C(1) and the adenine-nucleotide translocator. Embo J..

[210] Zollner A., Rödel G., Haid A. (1992). Molecular cloning and characterization of the Saccharomyces cerevisiae CYT2 gene encoding cytochrome-c1–heme lyase. FEBS J..

[211] Van Loon A., Brandli A.W., Pesold-Hurt B., Blank D., Schatz G. (1987). Transport of proteins to the mitochondrial intermembrane space: The ‘matrix-targeting’ and the ‘sorting’ domains in the cytochrome c1 presequence. Embo J..

[212] Phillips J., Schmitt M.E., Brown T.A., Beckmann J.D., Trumpower B.L. (1990). Isolation and characterization of QCR9, a nuclear gene encoding the 7.3-kDa subunit 9 of the Saccharomyces cerevisiae ubiquinol-cytochrome c oxidoreductase complex. An intron-containing gene with a conserved sequence occurring in the intron of COX4. J. Biol. Chem..

[213] Brandt U., Uribe S., Schägger H., Trumpower B.L. (1994). Isolation and characterization of QCR10, the nuclear gene encoding the 8.5-kDa subunit 10 of the Saccharomyces cerevisiae cytochrome bc1 complex. J. Biol. Chem..

[214] Wasilewski M., Chojnacka K., Chacinska A. (2017). Protein trafficking at the crossroads to mitochondria. Biochim. Biophys. Acta. Mol. Cell Res..

[215] Fu W., Japa S., Beattie D.S. (1990). Import of the iron-sulfur protein of the cytochrome b.c1 complex into yeast mitochondria. J. Biol. Chem..

[216] Wagener N., Ackermann M., Funes S., Neupert W. (2011). A pathway of protein translocation in mitochondria mediated by the AAA-ATPase Bcs1. Mol. Cell..

[217] Kater L., Wagener N., Berninghausen O., Becker T., Neupert W., Beckmann R. (2020). Structure of the Bcs1 AAA-ATPase suggests an airlock-like translocation mechanism for folded proteins. Nat. Struct. Mol. Biol..

[218] Tang W.K., Borgnia M.J., Hsu A.L., Esser L., Fox T., de Val N., Xia D. (2020). Structures of AAA protein translocase Bcs1 suggest translocation mechanism of a folded protein. Nat. Struct. Mol. Biol..

[219] Cui T.Z., Smith P.M., Fox J.L., Khalimonchuk O., Winge D.R. (2012). Late-Stage Maturation of the Rieske Fe/S Protein: Mzm1 Stabilizes Rip1 but Does Not Facilitate Its Translocation by the AAA ATPase Bcs1. Mol. Cell. Biol..

[220] Sanchez E., Lobo T., Fox J.L., Zeviani M., Winge D.R., Fernandez-Vizarra E. (2013). LYRM7/MZM1L is a UQCRFS1 chaperone involved in the last steps of mitochondrial Complex III assembly in human cells. Biochim. Biophys. Acta Bioenerg..

[221] Brandt U., Yu L., Yu C.A., Trumpower B.L. (1993). The mitochondrial targeting presequence of the Rieske iron-sulfur protein is processed in a single step after insertion into the cytochrome bc1 complex in mammals and retained as a subunit in the complex. J. Biol. Chem..

[222] Bottani E., Cerutti R., Harbour M.E., Ravaglia S., Dogan S.A., Giordano C., Fearnley I.M., D’Amati G., Viscomi C., Fernandez-Vizarra E. (2017). TTC19 Plays a Husbandry Role on UQCRFS1 Turnover in the Biogenesis of Mitochondrial Respiratory Complex III. Mol. Cell.

[223] Ghezzi D., Arzuffi P., Zordan M., Da Re C., Lamperti C., Benna C., D’Adamo P., Diodato D., Costa R., Mariotti C. (2011). Mutations in TTC19 cause mitochondrial complex III deficiency and neurological impairment in humans and flies. Nat. Genet..

[224] Capaldi R.A. (1990). Structure and function of cytochrome c oxidase. Annu. Rev. Biochem..

[225] Vidoni S.H.M.E., Guerrero-Castillo S., Signes A., Ding S., Fearnley I.M., Taylor R.W., Tiranti V., Arnold S., Fernandez-Vizarra E., Zeviani M. (2017). MR-1S Interacts with PET100 and PET117 in Module-Based Assembly of Human Cytochrome c Oxidase. Cell Rep..

[226] Hayashi T., Asano Y., Shintani Y., Aoyama H., Kioka H., Tsukamoto O., Hikita M., Shinzawa-Itoh K., Takafuji K., Higo S. (2015). Higd1a is a positive regulator of cytochrome c oxidase. Proc. Natl. Acad. Sci. USA.

[227] Mick D., Dennerlein S., Wiese H., Reinhold R., Pacheu-Grau D., Lorenzi I., Sasarman F., Weraarpachai W., Shoubridge E.A., Warscheid B. (2012). MITRAC links mitochondrial protein translocation to respiratory-chain assembly and translational regulation. Cell.

[228] Dennerlein S., Oeljeklaus S., Jans D., Hellwig C., Bareth B., Jakobs S., Deckers M., Warscheid B., Rehling P. (2015). MITRAC7 Acts as a COX1-Specific Chaperone and Reveals a Checkpoint during Cytochrome c Oxidase Assembly. Cell Rep..

[229] Szklarczyk R., Wanschers B.F., Cuypers T.D., Esseling J.J., Riemersma M., van den Brand M.A., Gloerich J., Lasonder E., van den Heuvel L.P., Nijtmans L.G. (2012). Iterative orthology prediction uncovers new mitochondrial proteins and identifies C12orf62 as the human ortholog of COX14, a protein involved in the assembly of cytochrome c oxidase. Genome Biol..

[230] Clemente P., Peralta S., Cruz-Bermudez A., Echevarría L., Fontanesi F., Barrientos A., Fernandez-Moreno M.A., Garesse R. (2013). hCOA3 stabilizes cytochrome c oxidase 1 (COX1) and promotes cytochrome c oxidase assembly in human mitochondria. J. Biol. Chem..

[231] Mick D., Vukotic M., Piechura H., Meyer H.E., Warscheid B., Deckers M., Rehling P. (2010). Coa3 and Cox14 are essential for negative feedback regulation of COX1 translation in mitochondria. J. Cell Biol..

[232] Antonicka H., Leary S.C., Guercin G.H., Agar J.N., Horvath R., Kennaway N.G., Harding C.O., Jaksch M., Shoubridge E.A. (2003). Mutations in COX10 result in a defect in mitochondrial heme A biosynthesis and account for multiple, early-onset clinical phenotypes associated with isolated COX deficiency. Hum. Mol. Genet..

[233] Diaz F., Thomas C.K., Garcia S., Hernandez D., Moraes C.T. (2005). Mice lacking COX10 in skeletal muscle recapitulate the phenotype of progressive mitochondrial myopathies associated with cytochrome c oxidase deficiency. Hum. Mol. Genet..

[234] Hiser L., Di Valentin M., Hamer A.G., Hosler J.P. (2000). Cox11p is required for stable formation of the Cu(B) and magnesium centers of cytochrome c oxidase. J. Biol. Chem..

[235] Glerum D., Shtanko A., Tzagoloff A. (1996). Characterization of COX17, a yeast gene involved in copper metabolism and assembly of cytochrome oxidase. J. Biol. Chem..

[236] Bode M., Woellhaf M.W., Bohnert M., van der Laan M., Sommer F., Jung M., Zimmermann R., Schroda M., Herrmann J.M. (2015). Redox-regulated dynamic interplay between Cox19 and the copper-binding protein Cox11 in the intermembrane space of mitochondria facilitates biogenesis of cytochrome c oxidase. Mol. Biol. Cell.

[237] Mansilla N., Racca S., Gras D.E., Gonzalez D.H., Welchen E. (2018). The Complexity of Mitochondrial Complex IV: An Update of Cytochrome c Oxidase Biogenesis in Plants. Int. J. Mol. Sci..

[238] Bourens M., Boulet A., Leary S.C., Barrientos A. (2014). Human COX20 cooperates with SCO1 and SCO2 to mature COX2 and promote the assembly of cytochrome c oxidase. Hum. Mol. Genet..

[239] Lorenzi I., Oeljeklaus S., Aich A., Ronsör C., Callegari S., Dudek J., Warscheid B., Dennerlein S., Rehling P. (2018). The mitochondrial TMEM177 associates with COX20 during COX2 biogenesis. Biochim. Biophys. Acta Mol. Cell Res..

[240] Leary S.C., Sasarman F., Nishimura T., Shoubridge E.A. (2009). Human SCO2 is required for the synthesis of CO II and as a thiol-disulphide oxidoreductase for SCO1. Hum. Mol. Genet..

[241] Leary S., Cobine P.A., Kaufman B.A., Guercin G.H., Mattman A., Palaty J., Lockitch G., Winge D.R., Rustin P., Horvath R. (2007). The human cytochrome c oxidase assembly factors SCO1 and SCO2 have regulatory roles in the maintenance of cellular copper homeostasis. Cell Metab..

[242] Leary S., Kaufman B.A., Pellecchia G., Guercin G.H., Mattman A., Jaksch M., Shoubridge E.A. (2004). Human SCO1 and SCO2 have independent, cooperative functions in copper delivery to cytochrome c oxidase. Hum. Mol. Genet..

[243] Stroud D., Maher M.J., Lindau C., Vögtle F.N., Frazier A.E., Surgenor E., Mountford H., Singh A.P., Bonas M., Oeljeklaus S. (2015). COA6 is a mitochondrial complex IV assembly factor critical for biogenesis of mtDNA-encoded COX2. Hum. Mol. Genet..

[244] Pacheu-Grau D., Bareth B., Dudek J., Juris L., Vögtle F.N., Wissel M., Leary S.C., Dennerlein S., Rehling P., Deckers M. (2015). Cooperation between COA6 and SCO2 in COX2 maturation during cytochrome c oxidase assembly links two mitochondrial cardiomyopathies. Cell Metab..

[245] Aich A., Wang C., Chowdhury A., Ronsör C., Pacheu-Grau D., Richter-Dennerlein R., Dennerlein S., Rehling P. (2018). COX16 promotes COX2 metallation and assembly during respiratory complex IV biogenesis. eLife.

[246] Cerqua C., Morbidoni V., Desbats M.A., Doimo M., Frasson C., Sacconi S., Baldoin M.C., Sartori G., Basso G., Salviati L. (2018). COX16 is required for assembly of cytochrome c oxidase in human cells and is involved in copper delivery to COX2. Biochim. Biophys. Acta.

[247] Jonckheere A.I., Smeitink J.A., Rodenburg R.J. (2012). Mitochondrial ATP synthase: Architecture, function and pathology. J. Inherit. Metab. Dis..

[248] Abrahams J.P., Leslie A.G., Lutter R., Walker J.E. (1994). Structure at 2.8 A resolution of F1-ATPase from bovine heart mitochondria. Nature.

[249] Wittig I., Schagger H. (2008). Structural organization of mitochondrial ATP synthase. Biochim. Biophys. Acta.

[250] Ackerman S.H., Tzagoloff A. (1990). Identification of two nuclear genes (ATP11, ATP12) required for assembly of the yeast F1-ATPase. Proc. Natl. Acad. Sci. USA.

[251] He J., Ford H.C., Carroll J., Ding S., Fearnley I.M., Walker J.E. (2017). Persistence of the mitochondrial permeability transition in the absence of subunit c of human ATP synthase. Proc. Natl. Acad. Sci. USA.

[252] Walker J.E. (2013). The ATP synthase: The understood, the uncertain and the unknown. Biochem. Soc. Trans..

[253] Dyer M.R., Walker J.E. (1993). Sequences of members of the human gene family for the c subunit of mitochondrial ATP synthase. Biochem. J..

[254] Yan W.L., Lerner T.J., Haines J.L., Gusella J.F. (1994). Sequence analysis and mapping of a novel human mitochondrial ATP synthase subunit 9 cDNA (ATP5G3). Genomics.

[255] Van Bloois E., Haan G.J., de Gier J.W., Oudega B., Luirink J. (2004). F1F0 ATP synthase subunit c is targeted by the SRP to YidC in the E. coli inner membrane. FEBS Lett..

[256] Kolli R., Soll J., Carrie C. (2018). Plant Mitochondrial Inner Membrane Protein Insertion. Int. J. Mol. Sci..

[257] Bahri H., Buratto J., Rojo M., Dompierre J.P., Salin B., Blancard C., Cuvellier S., Rose M., Ben Ammar Elgaaied A., Tetaud E. (2021). TMEM70 forms oligomeric scaffolds within mitochondrial cristae promoting in situ assembly of mammalian ATP synthase proton channel. Biochim. Biophys. Acta. Mol. Cell Res..

[258] Carroll J., He J., Ding S., Fearnley I.M., Walker J.E. (2021). TMEM70 and TMEM242 help to assemble the rotor ring of human ATP synthase and interact with assembly factors for complex I. Proc. Natl. Acad. Sci. USA.

[259] Ahting U., Floss T., Uez N., Schneider-Lohmar I., Becker L., Kling E., Iuso A., Bender A., de Angelis M.H., Gailus-Durner V. (2009). Neurological phenotype and reduced lifespan in heterozygous Tim23 knockout mice, the first mouse model of defective mitochondrial import. Biochim. Biophys. Acta Bioenerg..

[260] Devi L., Prabhu B.M., Galati D.F., Avadhani N.G., Anandatheerthavarada H.K. (2006). Accumulation of amyloid precursor protein in the mitochondrial import channels of human Alzheimer’s disease brain is associated with mitochondrial dysfunction. J. Neurosci..

[261] Sirk D., Zhu Z., Wadia J.S., Shulyakova N., Phan N., Fong J., Mills L.R. (2007). Chronic exposure to sub-lethal beta-amyloid (A beta) inhibits the import of nuclear-encoded proteins to mitochondria in differentiated PC12 cells. J. Neurochem..

[262] Cieri D., Vicario M., Vallese F., D’Orsi B., Berto P., Grinzato A., Catoni C., De Stefani D., Rizzuto R., Brini M. (2018). Tau localises within mitochondrial sub-compartments and its caspase cleavage affects ER-mitochondria interactions and cellular Ca2+ handling. Biochim. Et Biophys. Acta-Mol. Basis Dis..

[263] Hu Y., Li X.C., Wang Z.H., Luo Y., Zhang X.N., Liu X.P., Feng Q., Wang Q., Yue Z.Y., Chen Z. (2016). Tau accumulation impairs mitophagy via increasing mitochondrial membrane potential and reducing mitochondrial Parkin. Oncotarget.

[264] Parihar M.S., Parihar A., Fujita M., Hashimoto M., Ghafourifar P. (2008). Mitochondrial association of alpha-synuclein causes oxidative stress. Cell. Mol. Life Sci..

[265] Smith W.W., Jiang H.B., Pei Z., Tanaka Y., Morita H., Sawa A., Dawson V.L., Dawson T.M., Ross C.A. (2005). Endoplasmic reticulum stress and mitochondrial cell death pathways mediate A53T mutant alpha-synuclein-induced toxicity. Hum. Mol. Genet..

[266] Devi L., Raghavendran V., Prabhu B.M., Avadhani N.G., Anandatheerthavarada H.K. (2008). Mitochondrial import and accumulation of alpha-synuclein impair complex I in human dopaminergic neuronal cultures and Parkinson disease brain. J. Biol. Chem..

[267] Di Maio R., Barrett P.J., Hoffman E.K., Barrett C.W., Zharikov A., Borah A., Hu X.P., McCoy J., Chu C.T., Burton E.A. (2016). Alpha-Synuclein binds to TOM20 and inhibits mitochondrial protein import in Parkinson’s disease. Sci. Transl. Med..

[268] Bender A., Desplats P., Spencer B., Rockenstein E., Adame A., Elstner M., Laub C., Mueller S., Koob A.O., Mante M. (2013). TOM40 Mediates Mitochondrial Dysfunction Induced by alpha-Synuclein Accumulation in Parkinson’s Disease. PLoS ONE.

[269] Yano H., Baranov S.V., Baranova O.V., Kim J., Pan Y.C., Yablonska S., Carlisle D.L., Ferrante R.J., Kim A.H., Friedlander R.M. (2014). Inhibition of mitochondrial protein import by mutant huntingtin. Nat. Neurosci..

[270] Napoli E., Wong S., Hung C., Ross-Inta C., Bomdica P., Giulivi C. (2013). Defective mitochondrial disulfide relay system, altered mitochondrial morphology and function in Huntingtons disease. Hum. Mol. Genet..

[271] Fischer L.R., Igoudjil A., Magrane J., Li Y.J., Hansen J.M., Manfredi G., Glass J.D. (2011). SOD1 targeted to the mitochondrial intermembrane space prevents motor neuropathy in the Sod1 knockout mouse. Brain.

[272] Vijayvergiya C., Beal M.F., Buck J., Manfredi G. (2005). Mutant superoxide dismutase 1 forms aggregates in the brain mitochondrial matrix of amyotrophic lateral sclerosis mice. J. Neurosci..

[273] Pasinelli P., Belford M.E., Lennon N., Bacskai B.J., Hyman B.T., Trotti D., Brown R.H. (2004). Amyotrophic lateral sclerosis-associated SOD1 mutant proteins bind and aggregate with Bcl-2 in spinal cord mitochondria. Neuron.

[274] Li Q.A., Vande Velde C., Israelson A., Xie J., Bailey A.O., Dong M.Q., Chun S.J., Roy T., Winer L., Yates J.R. (2010). ALS-linked mutant superoxide dismutase 1 (SOD1) alters mitochondrial protein composition and decreases protein import. Proc. Natl. Acad. Sci. USA.

[275] Bannwarth S., Ait-El-Mkadem S., Chaussenot A., Genin E.C., Lacas-Gervais S., Fragaki K., Berg-Alonso L., Kageyama Y., Serre V., Moore D.G. (2014). A mitochondrial origin for frontotemporal dementia and amyotrophic lateral sclerosis through CHCHD10 involvement. Brain.

[276] Fernandez-Vizarra E., Zeviani M. (2021). Mitochondrial disorders of the OXPHOS system. FEBS Lett..

[277] Brischigliaro M., Zeviani M. (2021). Cytochrome c oxidase deficiency. Biochim. Biophys. Acta Bioenerg..

[278] Ghezzi D., Zeviani M., Garone C., Minczuk M. (2018). Human diseases associated with defects in assembly of OXPHOS complexes. Mitochondrial Diseases.

[279] Gorman G.S., Chinnery P.F., DiMauro S., Hirano M., Koga Y., McFarland R., Suomalainen A., Thorburn D.R., Zeviani M., Turnbull D.M. (2016). Mitochondrial diseases. Nat. Rev. Dis. Primers.

[280] Fiedorczuk K., Sazanov L.A. (2018). Mammalian Mitochondrial Complex I Structure and Disease-Causing Mutations. Trends Cell Biol..

[281] Shahrour M.A., Staretz-Chacham O., Dayan D., Stephen J., Weech A., Damseh N., Pri Chen H., Edvardson S., Mazaheri S., Saada A. (2017). Mitochondrial epileptic encephalopathy, 3-methylglutaconic aciduria and variable complex V deficiency associated with TIMM50 mutations. Clin. Genet..

[282] Reyes A., Melchionda L., Burlina A., Robinson A.J., Ghezzi D., Zeviani M. (2018). Mutations in TIMM50 compromise cell survival in OxPhos-dependent metabolic conditions. EMBO Mol. Med..

[283] Tort F., Ugarteburu O., Texidó L., Gea-Sorlí S., García-Villoria J., Ferrer-Cortès X., Arias Á., Matalonga L., Gort L., Ferrer I. (2019). Mutations in TIMM50 cause severe mitochondrial dysfunction by targeting key aspects of mitochondrial physiology. Hum. Mutat..

[284] Roesch K., Curran S.P., Tranebjaerg L., Koehler C.M. (2002). Human deafness dystonia syndrome is caused by a defect in assembly of the DDP1/TIMM8a-TIMM13 complex. Hum. Mol. Genet..

[285] Pacheu-Grau D., Callegari S., Emperador S., Thompson K., Aich A., Topol S.E., Spencer E.G., McFarland R., Ruiz-Pesini E., Torkamani A. (2018). Mutations of the mitochondrial carrier translocase channel subunit TIM22 cause early-onset mitochondrial myopathy. Hum. Mol. Genet..

[286] Wei X., Du M., Xie J., Luo T., Zhou Y., Zhang K., Li J., Chen D., Xu P., Jia M. (2020). Mutations in TOMM70 lead to multi-OXPHOS deficiencies and cause severe anemia, lactic acidosis, and developmental delay. J. Hum. Genet..

[287] Zheng H., Koo E.H. (2011). Biology and pathophysiology of the amyloid precursor protein. Mol. Neurodegener..

[288] Mann V.M., Cooper J.M., Daniel S.E., Srai K., Jenner P., Marsden C.D., Schapira A.H.V. (1994). Complex-i, iron, and ferritin in parkinsons-disease substantia-nigra. Ann. Neurol..

[289] Bindoff L.A., Birchmachin M.A., Cartlidge N.E.F., Parker W.D., Turnbull D.M. (1991). Respiratory-chain abnormalities in skeletal-muscle from patients with parkinsons-disease. J. Neurol. Sci..

[290] Schapira A.H.V. (1999). Mitochondria in the aetiology and pathogenesis of Parkinson’s disease. Parkinsonism Relat. Disord..

[291] Macdonald R., Barnes K., Hastings C., Mortiboys H. (2018). Mitochondrial abnormalities in Parkinson’s disease and Alzheimer’s disease: Can mitochondria be targeted therapeutically?. Biochem. Soc. Trans..

[292] Iwai A., Masliah E., Yoshimoto M., Ge N.F., Flanagan L., Desilva H.A.R., Kittel A., Saitoh T. (1995). The precursor protein of non-a-beta component of alzheimers-disease amyloid is a presynaptic protein of the central-nervous-system. Neuron.

[293] Spillantini M.G., Schmidt M.L., Lee V.M.Y., Trojanowski J.Q., Jakes R., Goedert M. (1997). alpha-synuclein in Lewy bodies. Nature.

[294] Clayton D.F., George J.M. (1998). The synucleins: A family of proteins involved in synaptic function, plasticity, neurodegeneration and disease. Trends Neurosci..

[295] Jakes R., Spillantini M.G., Goedert M. (1994). Identification of two distinct synucleins from human brain. FEBS Lett.

[296] Masliah E., Rockenstein E., Veinbergs I., Mallory M., Hashimoto M., Takeda A., Sagara Y., Sisk A., Mucke L. (2000). Dopaminergic loss and inclusion body formation in alpha-synuclein mice: Implications for neurodegenerative disorders. Science.

[297] Banerjee R., Starkov A.A., Beal M.F., Thomas B. (2009). Mitochondrial dysfunction in the limelight of Parkinson’s disease pathogenesis. Biochim. Biophys. Acta Mol. Basis Dis..

[298] Martin L.J., Pan Y., Price A.C., Sterling W., Copeland N.G., Jenkins N.A., Price D.L., Lee M.K. (2006). Parkinson’s disease alpha-synuclein transgenic mice develop neuronal mitochondrial degeneration and cell death. J. Neurosci..

[299] Song D.D., Shults C.W., Sisk A., Rockenstein E., Masliah E. (2004). Enhanced substantia nigra mitochondrial pathology in human alpha-synuclein transgenic mice after treatment with MPTP. Exp. Neurol..

[300] Li W.W., Yang R., Guo J.C., Ren H.M., Zha X.L., Cheng J.S., Cai D.F. (2007). Localization of alpha-synuclein to mitochondria within midbrain of mice. Neuroreport.

[301] Parihar M.S., Parihar A., Fujita M., Hashimoto M., Ghafourifar P. (2009). Alpha-synuclein overexpression and aggregation exacerbates impairment of mitochondrial functions by augmenting oxidative stress in human neuroblastoma cells. Int. J. Biochem. Cell Biol..

[302] Tanner C.M., Kamel F., Ross G.W., Hoppin J.A., Goldman S.M., Korell M., Marras C., Bhudhikanok G.S., Kasten M., Chade A.R. (2011). Rotenone, Paraquat, and Parkinson’s Disease. Environ. Health Perspect..

[303] Pickrell A.M., Youle R.J. (2015). The Roles of PINK1, Parkin, and Mitochondrial Fidelity in Parkinson’s Disease. Neuron.

[304] Kazlauskaite A., Kondapalli C., Gourlay R., Campbell D.G., Ritorto M.S., Hofmann K., Alessi D.R., Knebel A., Trost M., Muqit M.M.K. (2014). Parkin is activated by PINK1-dependent phosphorylation of ubiquitin at Ser(65). Biochem. J..

[305] Kondapalli C., Kazlauskaite A., Zhang N., Woodroof H.I., Campbell D.G., Gourlay R., Burchell L., Walden H., Macartney T.J., Deak M. (2012). PINK1 is activated by mitochondrial membrane potential depolarization and stimulates Parkin E3 ligase activity by phosphorylating Serine 65. Open Biol..

[306] Lee Y., Stevens D.A., Kang S.U., Jiang H.S., Lee Y.I., Ko H.S., Scarffe L.A., Umanah G.E., Kang H., Ham S. (2017). PINK1 Primes Parkin-Mediated Ubiquitination of PARIS in Dopaminergic Neuronal Survival. Cell Rep..

[307] Bertolin G., Jacoupy M., Traver S., Ferrando-Miguel R., Saint Georges T., Grenier K., Ardila-Osorio H., Muriel M.P., Takahashi H., Lees A.J. (2015). Parkin maintains mitochondrial levels of the protective Parkinson’s disease-related enzyme 17-beta hydroxysteroid dehydrogenase type 10. Cell Death Differ..

[308] Gehrke S., Wu Z.H., Klinkenberg M., Sun Y.P., Auburger G., Guo S., Lu B.W. (2015). PINK1 and Parkin Control Localized Translation of Respiratory Chain Component mRNAs on Mitochondria Outer Membrane. Cell Metab..

[309] Macdonald M.E., Ambrose C.M., Duyao M.P., Myers R.H., Lin C., Srinidhi L., Barnes G., Taylor S.A., James M., Groot N. (1993). A novel gene containing a trinucleotide repeat that is expanded and unstable on huntingtons-disease chromosomes. Cell.

[310] Li H., Li S.H., Johnston H., Shelbourne P.F., Li X.J. (2000). Amino-terminal fragments of mutant huntingtin show selective accumulation in striatal neurons and synaptic toxicity. Nat. Genet..

[311] DiFiglia M., Sapp E., Chase K.O., Davies S.W., Bates G.P., Vonsattel J.P., Aronin N. (1997). Aggregation of huntingtin in neuronal intranuclear inclusions and dystrophic neurites in brain. Science.

[312] Orr A.L., Li S.H., Wang C.E., Li H., Wang J.J., Rong J., Xu X.S., Mastroberardino P.G., Greenamyre J.T., Li X.J. (2008). N-terminal mutant huntingtin associates with mitochondria and impairs mitochondrial trafficking. J. Neurosci..

[313] Yu Z.X., Li S.H., Evans J., Pillarisetti A., Li H., Li X.J. (2003). Mutant huntingtin causes context-dependent neurodegeneration in mice with Huntington’s disease. J. Neurosci..

[314] Bae B.I., Xu H., Igarashi S., Fujimuro M., Agrawal N., Taya Y., Hayward S.D., Moran T.H., Montell C., Ross C.A. (2005). p53 mediates cellular dysfunction and behavioral abnormalities in Huntington’s disease. Neuron.

[315] Benchoua A., Trioulier Y., Zala D., Gaillard M.C., Lefort N., Dufour N., Saudou F., Elalouf J.M., Hirsch E., Hantraye P. (2006). Involvement of mitochondrial complex II defects in neuronal death produced by N-terminus fragment of mutated Huntingtin. Mol. Biol. Cell.

[316] Brennan W.A., Bird E.D., Aprille J.R. (1985). Regional mitochondrial respiratory activity in huntingtons-disease brain. J. Neurochem..

[317] Damiano M., Starkov A.A., Petri S., Kipiani K., Kiaei M., Mattiazzi M., Beal M.F., Manfredi G. (2006). Neural mitochondrial Ca2+ capacity impairment precedes the onset of motor symptoms in G93A Cu/Zn-superoxide dismutase mutant mice. J. Neurochem..

[318] Kim J., Moody J.P., Edgerly C.K., Bordiuk O.L., Cormier K., Smith K., Beal M.F., Ferrante R.J. (2010). Mitochondrial loss, dysfunction and altered dynamics in Huntington’s disease. Hum. Mol. Genet..

[319] Lisowsky T. (1994). Erv1 is involved in the cell-division cycle and the maintenance of mitochondrial genomes in saccharomyces-cerevisiae. Curr. Genet..

[320] Becher D., Kricke J., Stein G., Lisowsky T. (1999). A mutant for the yeast scERV1 gene displays a new defect in mitochondrial morphology and distribution. Yeast.

[321] Di Fonzo A., Ronchi D., Lodi T., Fassone E., Tigano M., Lamperti C., Corti S., Bordoni A., Fortunato F., Nizzardo M. (2009). The Mitochondrial Disulfide Relay System Protein GFER Is Mutated in Autosomal-Recessive Myopathy with Cataract and Combined Respiratory-Chain Deficiency. Am. J. Hum. Genet..

[322] Browne S.E., Bowling A.C., MacGarvey U., Baik M.J., Berger S.C., Muqit M.M.K., Bird E.D., Beal M.F. (1997). Oxidative damage and metabolic dysfunction in Huntington’s disease: Selective vulnerability of the basal ganglia. Ann. Neurol..

[323] Gu M., Gash M.T., Mann V.M., JavoyAgid F., Cooper J.M., Schapira A.H.V. (1996). Mitochondrial defect in Huntington’s disease on caudate nucleus. Ann. Neurol..

[324] Browne S.E., Beal M.F. (2004). The energetics of Huntington’s disease. Neurochem. Res..

[325] Valentine J.S., Doucette P.A., Potter S.Z. (2005). Copper-zinc superoxide dismutase and amyotrophic lateral sclerosis. Annu. Rev. Biochem.

[326] Carri M.T., Cozzolino M. (2011). SOD1 and mitochondria in ALS: A dangerous liaison. J. Bioenerg. Biomembr..

[327] Cozzolino M., Rossi S., Mirra A., Carri M.T. (2015). Mitochondrial dynamism and the pathogenesis of Amyotrophic Lateral Sclerosis. Front. Cell. Neurosci..

[328] Crugnola V., Lamperti C., Lucchini V., Ronchi D., Peverelli L., Prelle A., Sciacco M., Bordoni A., Fassone E., Fortunato F. (2010). Mitochondrial Respiratory Chain Dysfunction in Muscle From Patients With Amyotrophic Lateral Sclerosis. Arch. Neurol..

[329] Corti S., Donadoni C., Ronchi D., Bordoni A., Fortunato F., Santoro D., Del Bo R., Lucchini V., Crugnola V., Papadimitriou D. (2009). Amyotrophic lateral sclerosis linked to a novel SOD1 mutation with muscle mitochondrial dysfunction. J. Neurol. Sci..

[330] Schon E.A., Przedborski S. (2011). Mitochondria: The Next (Neurode) Generation. Neuron.

[331] Sturtz L.A., Diekert K., Jensen L.T., Lill R., Culotta V.C. (2001). A fraction of yeast Cu,Zn-superoxide dismutase and its metallochaperone, CCS, localize to the intermembrane space of mitochondria—A physiological role for SOD1 in guarding against mitochondrial oxidative damage. J. Biol. Chem..

[332] Okado-Matsumoto A., Fridovich I. (2001). Subcellular distribution of superoxide dismutases (SOD) in rat liver—Cu,Zn-SOD in mitochondria. J. Biol. Chem..

[333] Murphy M.P. (2009). How mitochondria produce reactive oxygen species. Biochem. J..

[334] Hervias I., Beal M.F., Manfredi G. (2006). Mitochondrial dysfunction and amyotrophic lateral sclerosis. Muscle Nerve.

[335] Lehmer C., Schludi M.H., Ransom L., Greiling J., Junghanel M., Exner N., Riemenschneider H., van der Zee J., Van Broeckhoven C., Weydt P. (2018). A novel CHCHD10 mutation implicates a Mia40-dependent mitochondrial import deficit in ALS. EMBO Mol. Med..

[336] Wang T., Liu H., Itoh K., Oh S., Zhao L., Murata D., Sesaki H., Hartung T., Na C.H., Wang J. (2021). C9orf72 regulates energy homeostasis by stabilizing mitochondrial complex I assembly. Cell Metab..

[337] Baker M.J., Palmer C.S., Stojanovski D. (2014). Mitochondrial protein quality control in health and disease. Br. J. Pharm..

[338] Samluk L., Chroscicki P., Chacinska A. (2018). Mitochondrial protein import stress and signaling. Curr. Opin. Physiol..

[339] Quiros P.M., Mottis A., Auwerx J. (2016). Mitonuclear communication in homeostasis and stress. Nat. Rev. Mol. Cell Biol..

[340] Haynes C.M., Ron D. (2010). The mitochondrial UPR—Protecting organelle protein homeostasis. J. Cell Sci..

[341] Nargund A.M., Pellegrino M.W., Fiorese C.J., Baker B.M., Haynes C.M. (2012). Mitochondrial Import Efficiency of ATFS-1 Regulates Mitochondrial UPR Activation. Science.

[342] D’Amico D., Sorrentino V., Auwerx J. (2017). Cytosolic Proteostasis Networks of the Mitochondrial Stress Response. Trends Biochem Sci.

[343] Aldridge J.E., Horibe T., Hoogenraad N.J. (2007). Discovery of genes activated by the mitochondrial unfolded protein response (mtUPR) and cognate promoter elements. PLoS ONE.

[344] Nargund A.M., Fiorese C.J., Pellegrino M.W., Deng P., Haynes C.M. (2015). Mitochondrial and Nuclear Accumulation of the Transcription Factor ATFS-1 Promotes OXPHOS Recovery during the UPRmt. Mol. Cell.

[345] Haynes C.M., Yang Y., Blais S.P., Neubert T.A., Ron D. (2010). The Matrix Peptide Exporter HAF-1 Signals a Mitochondrial UPR by Activating the Transcription Factor ZC376.7 in C. elegans. Mol. Cell.

[346] Rolland S.G., Schneid S., Schwarz M., Rackles E., Fischer C., Haeussler S., Regmi S.G., Yeroslaviz A., Habermann B., Mokranjac D. (2019). Compromised Mitochondrial Protein Import Acts as a Signal for UPRmt. Cell Rep..

[347] Fiorese C.J., Schulz A.M., Lin Y.F., Rosin N., Pellegrino M.W., Haynes C.M. (2016). The Transcription Factor ATF5 Mediates a Mammalian Mitochondrial UPR. Curr. Biol..

[348] Kuhl I., Miranda M., Atanassov I., Kuznetsova I., Hinze Y., Mourier A., Filipovska A., Larsson N.G. (2017). Transcriptomic and proteomic landscape of mitochondrial dysfunction reveals secondary coenzyme Q deficiency in mammals. eLife.

[349] Quiros P.M., Prado M.A., Zamboni N., D’Amico D., Williams R.W., Finley D., Gygi S.P., Auwerx J. (2017). Multi-omics analysis identifies ATF4 as a key regulator of the mitochondrial stress response in mammals. J. Cell Biol..

[350] Labbadia J., Brielmann R.M., Neto M.F., Lin Y.F., Haynes C.M., Morimoto R.I. (2017). Mitochondrial Stress Restores the Heat Shock Response and Prevents Proteostasis Collapse during Aging. Cell Rep..

[351] Cooper J.F., Machiela E., Dues D.J., Spielbauer K.K., Senchuk M.M., Van Raamsdonk J.M. (2017). Activation of the mitochondrial unfolded protein response promotes longevity and dopamine neuron survival in Parkinson’s disease models. Sci Rep..

[352] Martinez B.A., Petersen D.A., Gaeta A.L., Stanley S.P., Caldwell G.A., Caldwell K.A. (2017). Dysregulation of the Mitochondrial Unfolded Protein Response Induces Non-Apoptotic Dopaminergic Neurodegeneration in C-elegans Models of Parkinson’s Disease. J. Neurosci..

[353] St Martin J.L., Klucken J., Outeiro T.F., Nguyen P., Keller-McGandy C., Cantuti-Castelvetri I., Grammatopoulos T.N., Standaert D.G., Hyman B.T., McLean P.J. (2007). Dopaminergic neuron loss and up-regulation of chaperone protein mRNA induced by targeted over-expression of alpha-synuclein in mouse substantia nigra. J. Neurochem..

[354] Gorman A.M., Szegezdi E., Quigney D.J., Samali A. (2005). Hsp27 inhibits 6-hydroxydopamine-induced cytochrome c release and apoptosis in PC12 cells. Biochem. Biophys. Res. Commun..

[355] Klucken J., Shin Y., Masliah E., Hyman B.T., McLean P.J. (2004). Hsp70 reduces alpha-synuclein aggregation and toxicity. J. Biol. Chem..

[356] Riar A.K., Burstein S.R., Palomo G.M., Arreguin A., Manfredi G., Germain D. (2017). Sex specific activation of the ER alpha axis of the mitochondrial UPR (UPRmt) in the G93A-SOD1 mouse model of familial ALS. Hum. Mol. Genet..

[357] Pharaoh G., Sataranatarajan K., Street K., Hill S., Gregston J., Ahn B., Kinter C., Kinter M., Van Remmen H. (2019). Metabolic and Stress Response Changes Precede Disease Onset in the Spinal Cord of Mutant SOD1 ALS Mice. Front. Neurosci..

[358] Shen Y., Ding M., Xie Z.H., Liu X.T., Yang H., Jin S.Q., Xu S.L., Zhu Z.Y., Wang Y., Wang D.W. (2020). Activation of Mitochondrial Unfolded Protein Response in SHSY5Y Expressing APP Cells and APP/PS1 Mice. Front. Cell. Neurosci..

[359] Beck J.S., Mufson E.J., Counts S.E. (2016). Evidence for Mitochondrial UPR Gene Activation in Familial and Sporadic Alzheimer’s Disease. Curr. Alzheimer Res..

[360] Regitz C., Fitzenberger E., Mahn F.L., Dussling L.M., Wenzel U. (2016). Resveratrol reduces amyloid-beta (A beta(1-42))-induced paralysis through targeting proteostasis in an Alzheimer model of Caenorhabditis elegans. Eur. J. Nutr..

[361] Poveda-Huertes D., Matic S., Marada A., Habernig L., Licheva M., Myketin L., Gilsbach R., Tosal-Castano S., Papinski D., Mulica P. (2020). An Early mtUPR: Redistribution of the Nuclear Transcription Factor Rox1 to Mitochondria Protects against Intramitochondrial Proteotoxic Aggregates. Mol. Cell.

[362] Wrobel L., Topf U., Bragoszewski P., Wiese S., Sztolsztener M.E., Oeljeklaus S., Varabyova A., Lirski M., Chroscicki P., Mroczek S. (2015). Mistargeted mitochondrial proteins activate a proteostatic response in the cytosol. Nature.

[363] Papa L., Germain D. (2011). Estrogen receptor mediates a distinct mitochondrial unfolded protein response. J. Cell Sci..

[364] Callegari S., Dennerlein S. (2018). Sensing the Stress: A Role for the UPRmt and UPRam in the Quality Control of Mitochondria. Front. Cell. Dev. Biol..

[365] Hegde A.N., Upadhya S.C. (2011). Role of ubiquitin-proteasome-mediated proteolysis in nervous system disease. Biochim. Biophys. Acta Gene Regul. Mech..

[366] Dennissen F.J.A., Kholod N., van Leeuwen F.W. (2012). The ubiquitin proteasome system in neurodegenerative diseases: Culprit, accomplice or victim?. Prog. Neurobiol..

[367] Ciechanover A., Kwon Y.T. (2015). Degradation of misfolded proteins in neurodegenerative diseases: Therapeutic targets and strategies. Exp. Mol. Med..

[368] Tai H.C., Serrano-Pozo A., Hashimoto T., Frosch M.P., Spires-Jones T.L., Hyman B.T. (2012). The Synaptic Accumulation of Hyperphosphorylated Tau Oligomers in Alzheimer Disease Is Associated With Dysfunction of the Ubiquitin-Proteasome System. Am. J. Pathol..

[369] Wang X.W., Chen X.J. (2015). A cytosolic network suppressing mitochondria-mediated proteostatic stress and cell death. Nature.

[370] Gerbasi V.R., Link A.J. (2007). The myotonic dystrophy type 2 protein ZNF9 is part of an ITAF complex that promotes cap-independent translation. Mol. Cell. Proteom..

[371] Matsuo Y., Granneman S., Thoms M., Manikas R.G., Tollervey D., Hurt E. (2014). Coupled GTPase and remodelling ATPase activities form a checkpoint for ribosome export. Nature.

[372] Pakos-Zebrucka K., Koryga I., Mnich K., Ljujic M., Samali A., Gorman A.M. (2016). The integrated stress response. EMBO Rep..

[373] Elden A.C., Kim H.J., Hart M.P., Chen-Plotkin A.S., Johnson B.S., Fang X.D., Armakola M., Geser F., Greene R., Lu M.M. (2010). Ataxin-2 intermediate-length polyglutamine expansions are associated with increased risk for ALS. Nature.

[374] Chartier-Harlin M.C., Dachsel J.C., Vilarino-Guell C., Lincoln S.J., Lepretre F., Hulihan M.M., Kachergus J., Milnerwood A.J., Tapia L., Song M.S. (2011). Translation Initiator EIF4G1 Mutations in Familial Parkinson Disease. Am. J. Hum. Genet..

[375] Coyne L.P., Chen X.J. (2018). mPOS is a novel mitochondrial trigger of cell death—Implications for neurodegeneration. FEBS Lett..

[376] Weidberg H., Amon A. (2018). MitoCPR-A surveillance pathway that protects mitochondria in response to protein import stress. Science.

[377] Tang M.Z., Luo X.L., Huang Z., Chen L.X. (2018). MitoCPR: A novel protective mechanism in response to mitochondrial protein import stress. Acta Biochim. Biophys. Sin..

[378] Martensson C.U., Priesnitz C., Song J.Y., Ellenrieder L., Doan K.N., Boos F., Floerchinger A., Zufall N., Oeljeklaus S., Warscheid B. (2019). Mitochondrial protein translocation-associated degradation. Nature.

[379] Neuber O., Jarosch E., Volkwein C., Walter J., Sommer T. (2005). Ubx2 links the Cdc48 complex to ER-associated protein degradation. Nat. Cell Biol..

[380] Schuberth C., Buchberger A. (2005). Membrane-bound Ubx2 recruits Cdc48 to ubiquitin ligases and their substrates to ensure efficient ER-associated protein degradation. Nat. Cell Biol..

[381] Pereira G.C., Allen W.J., Watkins D.W., Buddrus L., Noone D., Liu X., Richardson A.P., Chacinska A., Collinson I. (2019). A High-Resolution Luminescent Assay for Rapid and Continuous Monitoring of Protein Translocation across Biological Membranes. J. Mol. Biol..

