# Supplementary material for: Interplay between Mitochondrial Protein Import and Respiratory Complexes Assembly in Neuronal Health and Degeneration
Source: Life (Basel). 2021 May 11;11(5):432. doi: 10.3390/life11050432 (PMC8151517; doi:10.3390/life11050432)
Supplement: Supplementary file 1 [file life-11-00432-s001.zip › life-1177847-supplementary.pdf]

Review

Interplay between Mitochondrial Protein Import and Respiratory Complexes Assembly in Neuronal Health and Degeneration

Hope I. Needs[1], Margherita Protasoni[2], Jeremy M. Henley[1,3], Julien Prudent[2], Ian Collinson[1,#] and Gonçalo C. Pereira[2,*]

[1] School of Biochemistry, University of Bristol, Bristol, United Kingdom
[2] Medical Research Council - Mitochondrial Biology Unit, University of Cambridge, Cambridge, United Kingdom
[3] Centre for Neuroscience and Regenerative Medicine, Faculty of Science, University of Technology Sydney, Ultimo, NSW, Australia
* Correspondence: g.pereira@mrc-mbu.cam.ac.uk; ian.collinson@bristol.ac.uk

Supplementary Material

Supplementary Table 1 – Supporting Table for Main Figure 3 on the Assembly of human Respiratory Complexes. The human subunits and major assembly factors are shown alongside with their yeast orthologs counterparts. Orthologs were checked in the Alliance of Genome Resource Database, release 4.0.0. Subunits are grouped by their assembly module, whenever the respiratory complex assembly is in modular fashion. For the assembly factors the column represents the subunit/module they help with. Protein features, N-terminal targeting sequence (MTS) and transmembrane domains were extracted from Uniprot annotated entries, unless it is supported by previous works in the literature. Similarly, for the proteins where there is no practical evidence for their import route, we inferred the most probable import pathway they use.

**Respiratory Complex**	**Yeast Ortholog**<br>Gene Name/ Access. Number	**Module**	**Pre-Assembly Localisation, Features, and Import Route**
Structural Subunits of CI			
ND1	N/A	ND1/P_P-a	mtDNA-encoded;<br>IMM protein;

			Inserted via OXA1L.
NDUFA3	N/A	ND1/P_P-a	IMM protein;<br>No N-terminal targeting sequence;<br>Unknown import route.
NDUFA8	N/A	ND1/P_P-a	IMS protein;<br>Imported through the MIA pathway [1].
NDUFA11	N/A	ND1/P_P-a	IMM protein;<br>Expected to be inserted via TIM22 [2].
NDUFA13	N/A	ND1/P_P-a	IMM protein;<br>No N-terminal targeting sequence;<br>Residues 20–30 and 40–60 are mitochondrial localisation signals [3];<br>Unknown import route.
ND2	N/A	ND2/P_P-b	mtDNA-encoded;<br>IMM protein;<br>Inserted via OXA1L.
ND3	N/A	ND2/P_P-b	mtDNA-encoded;<br>IMM protein;<br>Inserted via OXA1L.
ND4L	N/A	ND2/P_P-b	mtDNA-encoded;<br>IMM protein;<br>Inserted via OXA1L.
ND6	N/A	ND2/P_P-b	mtDNA-encoded;<br>IMM protein;<br>Inserted via OXA1L.
NDUFA10	N/A	ND2/P_P-b	Matrix protein;<br>N-terminal targeting sequence;<br>Expected to be imported through $TIM23^{MOTOR}$.
NDUFA1	N/A	ND2/P_P-b	IMM protein;<br>No N-terminal targeting sequence;<br>Residues 1-27 are mitochondrial localisation signals [3, 4];<br>Expected to be imported through $TIM23^{SORT}$ [3, 4].
NDUFC1	N/A	ND2/P_P-b	IMM protein;<br>N-terminal targeting sequence;<br>Expected to be imported through $TIM23^{SORT}$.

NDUFC2	N/A	ND2/P_P-b	IMM protein;<br>No N-terminal targeting sequence;<br>Expected to be inserted via TIM22 [5].
NDUFS5	N/A	ND2/P_P-b	IMS protein;<br>Imported through the MIA pathway [1].
ND4	N/A	ND4/$P_{D\text{-}a}$	mtDNA-encoded;<br>IMM protein;<br>Inserted via OXA1L.
NDUFB1	N/A	ND4/$P_{D\text{-}a}$	IMM protein;<br>No N-terminal targeting sequence;<br>Unknown import route.
NDUFB4	N/A	ND4/$P_{D\text{-}a}$	IMM protein;<br>No N-terminal targeting sequence;<br>Unknown import route.
NDUFB5	N/A	ND4/$P_{D\text{-}a}$	IMM protein;<br>N-terminal targeting sequence;<br>Expected to be imported through $TIM23^{SORT}$.
NDUFB6	N/A	ND4/$P_{D\text{-}a}$	IMM protein;<br>No N-terminal targeting sequence;<br>Unknown import route.
NDUFB10	N/A	ND4/$P_{D\text{-}a}$	IMS protein;<br>Imported through the MIA pathway [1].
NDUFB11	N/A	ND4/$P_{D\text{-}a}$	IMM protein;<br>N-terminal targeting sequence;<br>Expected to be imported through $TIM23^{SORT}$.
ND5	N/A	ND5/$P_{D\text{-}b}$	mtDNA-encoded;<br>IMM protein;<br>Inserted via OXA1L.
NDUFAB1	N/A	ND5/$P_{D\text{-}b}$	Matrix protein;<br>N-terminal targeting sequence;<br>Expected to be imported through $TIM23^{MOTOR}$.
NDUFB2	N/A	ND5/$P_{D\text{-}b}$	Matrix protein;<br>N-terminal targeting sequence;<br>Expected to be imported through $TIM23^{MOTOR}$.

NDUFB3	N/A	ND5/$P_{D\text{-}b}$	IMM protein;<br>No N-terminal targeting sequence;<br>Unknown import route.
NDUFB7	N/A	ND5/$P_{D\text{-}b}$	IMS protein;<br>Imported through the MIA pathway [1].
NDUFB8	N/A	ND5/$P_{D\text{-}b}$	IMM protein;<br>N-terminal targeting sequence;<br>Expected to be imported through TIM23^{SORT} [6].
NDUFB9	N/A	ND5/$P_{D\text{-}b}$	No N-terminal targeting sequence;<br>Unknown import route.
NDUFA5	N/A	Q	No N-terminal targeting sequence;<br>Unknown import route.
NDUFA6	N/A	Q	No N-terminal targeting sequence;<br>Unknown import route.
NDUFA7	N/A	Q	No N-terminal targeting sequence;<br>Unknown import route.
NDUFA9	N/A	Q	Matrix protein;<br>N-terminal targeting sequence;<br>Expected to be imported through TIM23^{MOTOR}.
NDUFS2	N/A	Q	Matrix protein;<br>N-terminal targeting sequence;<br>Expected to be imported through TIM23^{MOTOR}.
NDUFS3	N/A	Q	Matrix protein;<br>N-terminal targeting sequence;<br>Expected to be imported through TIM23^{MOTOR}.
NDUFS7	N/A	Q	Matrix protein;<br>N-terminal targeting sequence;<br>Expected to be imported through TIM23^{MOTOR}.
NDUFS8	N/A	Q	Matrix protein;<br>N-terminal targeting sequence;<br>Expected to be imported through TIM23^{MOTOR}.
NDUFS1	N/A	N	Matrix protein;<br>N-terminal targeting sequence;<br>Expected to be imported through TIM23^{MOTOR}.

NDUFS4	N/A	N/Q	Matrix protein;<br>N-terminal targeting sequence;<br>Expected to be imported through $TIM23^{MOTOR}$.
NDUFS6	N/A	N/Q	Matrix protein;<br>N-terminal targeting sequence;<br>Expected to be imported through $TIM23^{MOTOR}$.
NDUFV1	N/A	N	Matrix protein;<br>N-terminal targeting sequence;<br>Expected to be imported through $TIM23^{MOTOR}$.
NDUFV2	N/A	N	Matrix protein;<br>N-terminal targeting sequence;<br>Expected to be imported through $TIM23^{MOTOR}$.
NDUFV3	N/A	N/Q	Matrix protein;<br>N-terminal targeting sequence;<br>Expected to be imported through $TIM23^{MOTOR}$.
NDUFA2	N/A	N	Matrix protein;<br>N-terminal targeting sequence;<br>Expected to be imported through $TIM23^{MOTOR}$.
NDUFA12	N/A	N/Q	No N-terminal targeting sequence;<br>Unknown import route.
Assembly Factors for CI			
ACAD9	N/A	Assists in $ND2/P_P$-b module	IMM protein [7];<br>N-terminal targeting sequence;<br>Expected to be imported through $TIM23^{SORT}$.
ECSIT	N/A	Assists in $ND2/P_P$-b module	Matrix protein;<br>N-terminal targeting sequence;<br>Expected to be imported through $TIM23^{MOTOR}$.
FOXRED1	N/A	Assists in $ND4/P_{D-a}$ module	IMM protein;<br>No N-terminal targeting sequence;<br>Unknown import route [8].
ATP5SL	N/A	Assists in $ND4/P_{D-a}$ module	No N-terminal targeting sequence;<br>Unknown import route.

TMEM70	N/A	Assists in ND4/P_{D-a} module	IMM protein;<br>N-terminal targeting sequence;<br>Unknown import route.
NDUFAF1	N/A	Assists in ND1//P_P-a and N modules	Matrix protein;<br>N-terminal targeting sequence;<br>Expected to be imported through $TIM23^{MOTOR}$.
NDUFAF2	N/A	Assists in N module	Matrix protein;<br>N-terminal targeting sequence;<br>Expected to be imported through $TIM23^{MOTOR}$.
NDUFAF3	N/A	Assists in Q module	IMM protein [9];<br>No N-terminal targeting sequence;<br>Unknown import route [8].
NDUFAF4	N/A	Assists in Q module	IMM protein [9];<br>No N-terminal targeting sequence;<br>Unknown import route.
NDUFAF5	N/A	Assists in Q module	Matrix protein;<br>N-terminal targeting sequence;<br>Expected to be imported through $TIM23^{MOTOR}$.
NDUFAF6	N/A	Assists in ND1/P_{p-a} module	Matrix protein;<br>N-terminal targeting sequence;<br>Expected to be imported through $TIM23^{MOTOR}$.
NDUFAF7	N/A	Assists in Q module	Matrix protein [10];<br>N-terminal targeting sequence;<br>Expected to be imported through $TIM23^{MOTOR}$.
NUBPL	N/A	Assists in N and Q modules	Matrix protein;<br>N-terminal targeting sequence;<br>Expected to be imported through $TIM23^{MOTOR}$.
TIMMDC1	N/A	Assists in ND1/P_P-a	IMM protein;<br>No N-terminal targeting sequence;<br>Expected to be inserted via TIM22 [5].

TMEM126B	N/A	Assists in ND2/P_P-b module	IMM protein;<br>No N-terminal targeting sequence;<br>Expected to be inserted via TIM22 [5].
TMEM186	N/A	Assists in ND2/P_P-b module	IMM protein;<br>N-terminal targeting sequence;<br>imported via the TIM23^{SORT} [11].
DMAC1/TMEM261	N/A	Assists in ND5/P_{D-b} module	IMM protein;<br>No N-terminal targeting sequence;<br>Unknown import route.
COA1	COA1 / YIL157C	Assists in ND2/Pp-b module	IMM protein;<br>No N-terminal targeting sequence;<br>Unknown import route.
Structural Subunits of CII			
SDHA	SDH1 / YKL148C	Non-modular	Matrix protein;<br>N-terminal targeting sequence inferred by similarity with porcine sequence;<br>Expected to be imported through TIM23^{MOTOR}.
SDHB	SDH2 / YLL041C	Non-modular	Matrix protein;<br>N-terminal targeting sequence;<br>Expected to be imported through TIM23^{MOTOR}.
SDHC	SDH3 / YKL141W	Hydrophobic module	IMM protein;<br>N-terminal targeting sequence;<br>expected to be imported through TIM23^{SORT} [12].
SDHD	SDH4 / YDR178W	Hydrophobic module	IMM protein;<br>N-terminal targeting sequence;<br>Expected to be imported through TIM23^{SORT} [12].
Assembly Factors for CII			
SDHAF1	SDH6 / YDR379C-A	Maturation of SDHB	Matrix protein;<br>Non-cleavable N-terminal targeting sequence.
SDHAF2	SDH5 / YOL071W	Flavinylation of SDHA	Matrix protein;<br>N-terminal targeting sequence;<br>Expected to be imported through TIM23^{MOTOR} [13].
SDHAF3	SDH7 / YDR511W	Maturation of SDHB	Matrix protein;<br>N-terminal targeting sequence;

			Expected to be imported through TIM23^{MOTOR}.
SDHAF4	SDH8/ YBR269C	Interacts with mature SDHA	Matrix protein;<br>N-terminal targeting sequence;<br>Expected to be imported through TIM23^{MOTOR}.
Structural Subunits of CIII			
UQCRC1	COR1 / YBL045C	Non-modular	Matrix protein;<br>N-terminal targeting sequence;<br>Expected to be imported through TIM23^{MOTOR}.
UQCRC2	QCR2 / YPR191W	Non-modular	Matrix protein;<br>N-terminal targeting sequence;<br>Expected to be imported through TIM23^{MOTOR}.
MTCYB	COB / Q0105	Non-modular	mtDNA-encoded<br>IMM protein;<br>Inserted via OXA1L.
CYC1	CYT1 / YOR065W	Intermediate-assembly module	IMM protein;<br>N-terminal targeting sequence;<br>Unclear if it is imported into the matrix and then re-located to the IMM or inserted in the IMM through lateral release (stop-transfer) via TIM23^{SORT} [14, 15].
UQCRFS1	RIP1 / YEL024W	Non-modular	IMM protein;<br>Targeted to the matrix and exported in the IMS via BCS1 [16].
UQCRH	QCR6 / YFR033C	Intermediate-assembly module	Matrix protein;<br>N-terminal targeting sequence;<br>Expected to be imported through TIM23^{MOTOR}.
UQCRB	QCR7 / YDR529C	Non-modular	Matrix protein<br>No N-terminal targeting sequence;<br>Unknown import route.
UQCRQ	QCR8 / YJL166W	Non-modular	IMM protein;<br>No N-terminal targeting sequence;<br>Unknown import route.
UQCR10	QCR9 / YGR183C	Intermediate-assembly module	IMM protein;<br>No N-terminal targeting sequence;<br>Unknown import route.
UQCR11	N/A	Non-modular	IMM protein;

			No N-terminal targeting sequence;<br>Unknown import route.
Assembly Factors for CIII			
UQCC1	CBP3 / YPL215W	MTCYB	Matrix protein;<br>No N-terminal targeting sequence;<br>Unknown import route.
UQCC2	N/A	MTCYB	Matrix protein;<br>N-terminal targeting sequence;<br>Expected to be imported through TIM23^{MOTOR}.
UQCC3	N/A	MTCYB	IMM protein;<br>No N-terminal targeting sequence;<br>Unknown import route.
HCCS	Cyt2	CYC1	IMM-associated protein;<br>No N-terminal targeting sequence;<br>Unknown import route [17].
BCS1L	BCS1 / YDR375C	UQCRFS1	IMM protein;<br>No N-terminal targeting sequence;<br>Expected to be imported through TIM23^{MOTOR} based on yeast ortholog [18].
LYRM7	MZM1 / YDR493W	UQCRFS1	Matrix protein;<br>No N-terminal targeting sequence;<br>Unknown import route.
TTC19	N/A	UQCRFS1	IMM protein;<br>N-terminal targeting sequence;<br>Unknown import route.
Structural Subunits of CIV			
MTCO1	COX1 / Q0045	MTCO1-module	mtDNA-encoded;<br>IMM protein;<br>Inserted via OXA1L.
COX4I1	Paralogs<br>COX5A / YNL052W and<br>COX5B / YIL111W	Early-assembly-module	IMM protein;<br>N-terminal targeting sequence<br>Imported via TIM23^{SORT} [19].

COX5A	COX6 / YHR051W	Early-assembly-module	Matrix protein<br>N-terminal targeting sequence;<br>Expected to be imported through $TIM23^{MOTOR}$.
MTCO2	COX2 / Q0250	MTCO2-module	mtDNA-encoded;<br>IMM protein;<br>Inserted via OXA1L.
COX5B	COX4 / YGL187C	MTCO2-module	Matrix protein<br>N-terminal targeting sequence;<br>Expected to be imported through $TIM23^{MOTOR}$.
COX6C	N/A	MTCO2-module	IMM protein;<br>No N-terminal targeting sequence;<br>Interacts with TIM21, expected to be imported through $TIM23^{SORT}$ [6].
COX7B	N/A	MTCO2-module	IMM protein;<br>N-terminal targeting sequence;<br>Expected to be imported through $TIM23^{SORT}$.
COX7C	COX8 / YLR395C	MTCO2-module	IMM protein;<br>N-terminal targeting sequence;<br>Expected to be imported through $TIM23^{SORT}$.
COX8A	N/A	MTCO2-module	IMM protein;<br>N-terminal targeting sequence;<br>Expected to be imported through $TIM23^{SORT}$.
MTCO3	COX3 / Q0275	MTCO3-module	mtDNA-encoded;<br>IMM protein;<br>Inserted via OXA1L.
COX6A1	COX13 / YGL191W	MTCO3-module	IMM protein;<br>N-terminal targeting sequence;<br>Expected to be imported through $TIM23^{SORT}$.
COX6A2	COX13 / YGL191W	MTCO3-module	IMM protein;<br>N-terminal targeting sequence;<br>Expected to be imported through $TIM23^{SORT}$.
COX6B1	COX12 / YLR038C	MTCO3-module	IMS protein;<br>No N-terminal targeting sequence;<br>Imported through the MIA pathway.

COX7A	COX7 / YMR256C	MTCO3-module	IMM protein;<br>N-terminal targeting sequence;<br>Expected to be imported through $TIM23^{SORT}$.
NDUFA4	N/A	N/A	IMM protein;<br>No N-terminal targeting sequence;<br>Unknown import route.
Assembly Factors for CIV			
TACO1	DPC29 / YGR021W	MTCO1 translation	Matrix protein;<br>No N-terminal targeting sequence;<br>Unknown import route.
LRPPRC	N/A	N/A	Matrix protein;<br>N-terminal targeting sequence;<br>Expected to be imported through $TIM23^{MOTOR}$.
FASTKD2	N/A	Post-transcriptional RNA maturation, ribosome biogenesis and translation.	Matrix protein;<br>Might contain an N-terminal targeting sequence;<br>Expected to be imported through $TIM23^{MOTOR}$.
COX10	COX10 / YPL172C	MTCO1-module;<br>Heme *a* synthesis	IMM protein;<br>Might contain an N-terminal targeting sequence;<br>Expected to be imported through $TIM23^{SORT}$ and others.
COX15	COX15 / YER141W	MTCO1-module;<br>Heme *a* synthesis	IMM protein;<br>N-terminal targeting sequence;<br>Expected to be imported through $TIM23^{SORT}$.
SURF1	SHY1 / YGR112W	MTCO1-module;<br>Insertion or stabilisation of heme a_3	IMM protein;<br>N-terminal targeting sequence;<br>Expected to be imported through $TIM23^{SORT}$.

COA6	COA6 / YMR244C-A	MTCO2-module; Copper homeostasis	IMS protein; Imported through the MIA pathway [20].
SCO1	SCO1 / YBR037C	MTCO2-module; Copper incorporation	IMM protein; N-terminal targeting sequence; Expected to be imported through $TIM23^{SORT}$.
SCO2	SCO2 / YBR024W	MTCO2-module; Copper incorporation	IMM protein; N-terminal targeting sequence; Expected to be imported through $TIM23^{SORT}$.
COX11	COX11 / YPL132W	MTCO1-module; Copper chaperone	IMM protein; Might contain an N-terminal targeting sequence; Expected to be imported through $TIM23^{SORT}$.
COX16	COX16 / YJL003W	MTCO2-module	IMM protein; No N-terminal targeting sequence; Unknown import route.
COX17	COX17 / YLL009C	MTCO1-module; Copper transfer	IMS protein; Imported through the MIA pathway in yeast [21].
COX19	COX19 / YLL018C-A	MTCO1-module; COX11 stabilisation	IMS protein; Imported through the MIA pathway.
COA3/MITRAC12	COA3 / YJL062W-A	MTCO1-module	IMM protein; N-terminal targeting sequence [22]; Possibly interacts with OXA1 [22].
COA7	N/A	N/A	IMS protein; Imported through the MIA pathway.
COX14/c12orf62	COX14 / YML129C	MTCO1-module	IMM protein; Contains mitochondrial targeting sequences [22].

CMC1	CMC1 / YKL137W	MTCO1-module	IMS protein;<br>Imported through the MIA pathway [23].
COX20/FAM36A	COX20 / YDR231C	MTCO2-module;<br>Copper metalation	IMM protein;<br>No N-terminal targeting sequence;<br>Imported through the MIA pathway [24].
PET100	PET100 / YDR079W	S3 intermediary	IMM protein;<br>No N-terminal targeting sequence [25];<br>Unknown import route.
PET117	PET117 / YER058W	S3 intermediary	Matrix protein;<br>N-terminal targeting sequence;<br>Expected to be imported through TIM23^{MOTOR}.
PNKD/MR-1S	Paralogs<br>GLO2 / YDR272W<br>GLO4 / YOR040W	S3 intermediary	IMM protein [26];<br>N-terminal targeting sequence;<br>Expected to be imported through TIM23^{SORT} [26].
COA8	N/A	N/A	IMM protein [27];<br>N-terminal targeting sequence;<br>Expected to be imported through TIM23^{SORT}.
COX18	COX18 / YGR062C	MTCO2-module	IMM protein;<br>N-terminal targeting sequence [28];<br>Expected to be imported through TIM23^{SORT}.
Structural Subunits of CV			
ATP5F1A	ATP1 / YBL099W	F1 catalytic head	Matrix protein;<br>N-terminal targeting sequence;<br>Expected to be imported through TIM23^{MOTOR}.
ATP5F1B	ATP2 / YJR121W	F1 catalytic head	Matrix protein;<br>N-terminal targeting sequence;<br>Expected to be imported through TIM23^{MOTOR}.
ATP5F1C	ATP3 / YBR039W	F1 central stalk	Matrix protein;<br>N-terminal targeting sequence;<br>Expected to be imported through TIM23^{MOTOR}.
ATP5F1D	ATP16 / YDL004W	F1 central stalk	Matrix protein;<br>N-terminal targeting sequence;<br>Expected to be imported through TIM23^{MOTOR}.

ATP5F1E	ATP15 / YPL271W	F1 central stalk	Matrix protein;<br>No N-terminal targeting sequence;<br>Expected to be imported through $TIM23^{MOTOR}$.
ATP5MC1			IMM protein;<br>N-terminal targeting sequence;<br>Expected to be imported through $TIM23^{SORT}$.
ATP5MC2	ATP9 / Q0130	Fo rotor	IMM protein;<br>N-terminal targeting sequence;<br>Expected to be imported through $TIM23^{SORT}$.
ATP5MC3			IMM protein;<br>N-terminal targeting sequence;<br>Expected to be imported through $TIM23^{SORT}$.
MT-ATP6	ATP6 / Q0085	Fo rotor	mtDNA-encoded;<br>IMM protein;<br>Imported via OXA1L.
MT-ATP8	ATP8 / Q0080	Fo rotor	mtDNA-encoded;<br>IMM protein;<br>Imported via OXA1L.
ATP5ME	ATP21 / YDR322C-A	Fo supernumera ry subunits	IMM protein;<br>No N-terminal targeting sequence;<br>Unknown import route.
ATP5MF	ATP17 / YDR377W	Fo supernumera ry subunits	IMM protein;<br>No N-terminal targeting sequence;<br>Unknown import route.
ATP5MG	ATP20 / YPR020W	Fo supernumera ry subunits	IMM protein;<br>No N-terminal targeting sequence;<br>Unknown import route.
ATP5MJ	ATP18 / YML081C-A	Fo supernumera ry subunits	IMM protein;<br>No N-terminal targeting sequence;<br>Unknown import route.
ATP5MK	ATP19 / YOL077W-A	Fo supernumera ry subunits	IMM protein;<br>No N-terminal targeting sequence;<br>Unknown import route.

ATP5PB	ATP4 / YPL078C	Fo peripheral stalk	Matrix protein;<br>N-terminal targeting sequence;<br>Expected to be imported through $TIM23^{MOTOR}$.
ATP5PD	ATP7 / YKL016C	Fo peripheral stalk	Matrix protein;<br>No N-terminal targeting sequence;<br>Unknown import route.
ATP5PF	ATP14 / YLR295C	Fo peripheral stalk	Matrix protein;<br>N-terminal targeting sequence;<br>Expected to be imported through $TIM23^{MOTOR}$.
ATP5PO	ATP5 / YDR298C	Fo peripheral stalk	Matrix protein;<br>N-terminal targeting sequence;<br>Expected to be imported through $TIM23^{MOTOR}$.
Assembly Factors for CV			
ATPAF1/ATP11	ATP11 / YNL315C	F_1-module	Matrix protein;<br>N-terminal targeting sequence;<br>Expected to be imported through $TIM23^{MOTOR}$.
ATPAF2/ATP12	ATP12 / YJL180C	F_1-module	Matrix protein;<br>N-terminal targeting sequence;<br>Expected to be imported through $TIM23^{MOTOR}$.
FMC1/C7orf55	FMC1 / YIL098C	F1-module	No N-terminal targeting sequence;<br>Unknown import route.
TMEM70	N/A	c-ring	IMM protein;<br>N-terminal targeting sequence;<br>Expected to be imported through $TIM23^{SORT}$.
TMEM242	N/A	c-ring	IMM protein;<br>No N-terminal targeting sequence;<br>Unknown import route.
ATP23	ATP23 / YNR020C	N/A	No N-terminal targeting sequence;<br>Imported through the MIA pathway in yeast [29].

References

1. Friederich, M.W., et al., *Mutations in the accessory subunit NDUFB10 result in isolated complex I deficiency and illustrate the critical role of intermembrane space import for complex I holoenzyme assembly.* Human Molecular Genetics, 2017. **26**(4): p. 702-716.
2. Jackson, T.D., et al., *The TIM22 complex mediates the import of Sideroflexins and is required for efficient mitochondrial one-carbon metabolism.* Molecular Biology of the Cell. **0**(0): p. mbc.E20-06-0390.
3. Lu, H. and X.M. Cao, *GRIM-19 is essential for maintenance of mitochondrial membrane potential.* Molecular Biology of the Cell, 2008. **19**(5): p. 1893-1902.
4. Yadava, N. and I.E. Scheffler, *Import and orientation of the MWFE protein in mitochondrial NADH-ubiquinone oxidoreductase.* Mitochondrion, 2004. **4**(1): p. 1-12.
5. Sanchez-Caballero, L., S. Guerrero-Castillo, and L. Nijtmans, *Unraveling the complexity of mitochondrial complex I assembly: A dynamic process.* Biochimica Et Biophysica Acta-Bioenergetics, 2016. **1857**(7): p. 980-990.
6. Mick, D.U., et al., *MITRAC Links Mitochondrial Protein Translocation to Respiratory-Chain Assembly and Translational Regulation.* Cell, 2012. **151**(7): p. 1528-1541.
7. Ensenauer, R., et al., *Human acyl-CoA dehydrogenase-9 plays a novel role in the mitochondrial beta-oxidation of unsaturated fatty acids.* Journal of Biological Chemistry, 2005. **280**(37): p. 32309-32316.
8. Formosa, L.E., et al., *Characterization of mitochondrial FOXRED1 in the assembly of respiratory chain complex I.* Human Molecular Genetics, 2015. **24**(10): p. 2952-2965.
9. Saada, A., et al., *Mutations in NDUFAF3 (C3ORF60), Encoding an NDUFAF4 (C6ORF66)-interacting Complex I Assembly Protein, Cause Fatal Neonatal Mitochondrial Disease.* American Journal of Human Genetics, 2009. **84**(6): p. 718-727.
10. Rendon, O.Z., et al., *The arginine methyltransferase NDUFAF7 is essential for complex I assembly and early vertebrate embryogenesis.* Human Molecular Genetics, 2014. **23**(19): p. 5159-5170.
11. Formosa, L.E., et al., *Dissecting the Roles of Mitochondrial Complex I Intermediate Assembly Complex Factors in the Biogenesis of Complex I.* Cell Reports, 2020. **31**(3): p. 19.
12. Van Vranken, J.G., et al., *Protein-mediated assembly of succinate dehydrogenase and its cofactors.* Critical Reviews in Biochemistry and Molecular Biology, 2015. **50**(2): p. 168-180.
13. Bezawork-Geleta, A., et al., *Mitochondrial matrix proteostasis is linked to hereditary paraganglioma: LON-mediated turnover of the human flavinylation factor SDH5 is regulated by its interaction with SDHA.* Faseb Journal, 2014. **28**(4): p. 1794-1804.
14. Ndi, M., et al., *Biogenesis of the bc(1) Complex of the Mitochondria! Respiratory Chain.* Journal of Molecular Biology, 2018. **430**(21): p. 3892-3905.
15. Arnold, I., et al., *Two distinct and independent mitochondrial targeting signals function in the sorting of an inner membrane protein, cytochrome c(1).* Journal of Biological Chemistry, 1998. **273**(3): p. 1469-1476.
16. Wegener, N., et al., *A Pathway of Protein Translocation in Mitochondria Mediated by the AAA-ATPase Bcsl.* Molecular Cell, 2011. **44**(2): p. 191-202.

17. Allen, J.W.A., *Cytochrome c biogenesis in mitochondria - Systems III and V.* Febs Journal, 2011. **278**(22): p. 4198-4216.
18. Folsch, H., et al., *Internal targeting signal of the BCS1 protein: a novel mechanism of import into mitochondria.* EMBO J, 1996. **15**(3): p. 479-87.
19. Priesnitz, C. and T. Becker, *Pathways to balance mitochondrial translation and protein import.* Genes & Development, 2018. **32**(19-20): p. 1285-1296.
20. Stroud, D.A., et al., *COA6 is a mitochondrial complex IV assembly factor critical for biogenesis of mtDNA-encoded COX2.* Human Molecular Genetics, 2015. **24**(19): p. 5404-5415.
21. Koch, J.R. and F.X. Schmid, *Mia40 targets cysteines in a hydrophobic environment to direct oxidative protein folding in the mitochondria.* Nature Communications, 2014. **5**: p. 10.
22. Watson, S.A. and G.P. McStay, *Functions of Cytochrome c Oxidase Assembly Factors.* International Journal of Molecular Sciences, 2020. **21**(19): p. 17.
23. Bourens, M., et al., *Role of Twin Cys-Xaa(9)-Cys Motif Cysteines in Mitochondrial Import of the Cytochrome c Oxidase Biogenesis Factor Cmc1.* Journal of Biological Chemistry, 2012. **287**(37): p. 31258-31269.
24. Nuebel, E., P. Manganas, and K. Tokatlidis, *Orphan proteins of unknown function in the mitochondrial intermembrane space proteome: New pathways and metabolic cross-talk.* Biochimica Et Biophysica Acta-Molecular Cell Research, 2016. **1863**(11): p. 2613-2623.
25. Lim, S.C., et al., *A Founder Mutation in PET100 Causes Isolated Complex IV Deficiency in Lebanese Individuals with Leigh Syndrome.* American Journal of Human Genetics, 2014. **94**(2): p. 209-222.
26. Ghezzi, D., et al., *Paroxysmal non-kinesigenic dyskinesia is caused by mutations of the MR-1 mitochondrial targeting sequence.* Hum Mol Genet, 2009. **18**(6): p. 1058-64.
27. Signes, A., et al., *APOPT1/COA8 assists COX assembly and is oppositely regulated by UPS and ROS.* Embo Molecular Medicine, 2019. **11**(1): p. 21.
28. Bourens, M. and A. Barrientos, *Human mitochondrial cytochrome c oxidase assembly factor COX18 acts transiently as a membrane insertase within the subunit 2 maturation module.* Journal of Biological Chemistry, 2017. **292**(19): p. 7774-7783.
29. Weckbecker, D., et al., *Atp23 biogenesis reveals a chaperone-like folding activity of Mia40 in the IMS of mitochondria.* Embo Journal, 2012. **31**(22): p. 4348-4358.
